# A riddle of culprit only vs multivessel or immediate vs staged revascularization in patients with non-ST elevation acute coronary syndrome: A meta-analysis

**DOI:** 10.1371/journal.pone.0310695

**Published:** 2025-03-18

**Authors:** Yudi Her Oktaviono, Jannatin Nisa Arnindita, Pandit Bagus Tri Saputra, Nabilah Azzah Putri Wairooy, Arlia Ayu Damayanti, Suryo Ardi Hutomo, Nando Reza Pratama, Makhyan Jibril Al Farabi, Faisal Yusuf Ashari

**Affiliations:** 1 Department of Cardiology and Vascular Medicine, Dr. Soetomo General Academic Hospital, Surabaya, Indonesia,; 2 Faculty of Medicine, Universitas Airlangga, Surabaya, Indonesia,; 3 Subspecialist Study Program of Cardiology and Vascular Medicine, Faculty of Medicine, Universitas Airlangga, Surabaya, Indonesia,; 4 Nuffield Department of Medicine, University of Oxford, Oxford, United Kingdom,; 5 Department of Biomedical Sciences, Faculty of Medicine, Universitas Airlangga, Surabaya, Indonesia,; 6 Faculty of Biology Medicine and Health Sciences, University of Manchester, Manchester, United Kingdom; PearResearch / Government Doon Medical College, INDIA

## Abstract

**Background:**

Percutaneous coronary intervention (PCI) is a revolutionary breakthrough in saving many lives from myocardial infarction. However, little is known about the PCI strategy in multivessel disease (MVD) Non-ST Elevation Acute Coronary Syndrome (NSTE-ACS) patients. Should complete revascularization be achieved or culprit-only is sufficient, then when the appropriate time of multivessel revascularization is, whether it is staged or immediate. Limited evidence is available on this matter compared to ST-elevation myocardial infarction (STEMI), even though NSTE-ACS patients carry poorer long-term prognoses compared to STEMI.

**Methods:**

A thorough search for appropriate studies was executed across PubMed, Embase, Medline, Science Direct, and Scopus databases until July 4th, 2023. The risk ratio (RR) underwent analysis through Review Manager 5.4.

**Results:**

Twenty-six studies with 222,350 MVD NSTE-ACS patients were included. Culprit-only revascularization was significantly related to a higher risk of non-fatal MI (RR: 1.41, 95% CI: 1.04-1.91, p = 0.03, I^2^: 65%) and all-repeat revascularization (RR 1.86, 95% CI 1.07-3.25, p = 0.03). While multistage multivessel revascularization was related to significantly higher all-cause mortality (RR: 1.73; 95% CI 1.43-2.10; p < 0.01; I^2^: 0%), TVR repeat (RR 1.38 95% CI 1.11-1.73, I2 =  18%, p = 0.004), and non-TVR repeat (RR 2.59; 95% CI 1,56-4.30; p = 0.0002; I^2^: 70%).

**Conclusion:**

Patients with MVD NSTE-ACS treated with multivessel revascularization showed more favorable results than culprit-only. One-stage multivessel revascularization resulted in fewer adverse events than multistage. Additionally, a comprehensive and methodical prospective investigation is required to validate the factors accountable for these outcomes.

## Background

Coronary artery disease (CAD) remains the leading cause of death worldwide. In the US, CAD affects > 18.2 million adults and is a leading cause of death, accounting for > 365,000 deaths annually [1]. Based on the electrocardiogram (ECG), ACS is divided into ST-segment elevation ACS and non-ST-segment elevation ACS, where non-ST segment elevation ACS is divided into NSTEMI and unstable angina [2]. Among 1 million patients admitted to the emergency room due to ACS, 70% of them suggest NSTE-ACS presentation in their ECG examination [3]. Compared to STEMI, NSTEMI patients are exposed to a greater risk of dying after hospital discharge, with 33.2% death rates in two years post-discharge compared to 16.4% in STEMI patients [4].

Revascularization in multivessel NSTE-ACS patients could reduce 24 months of mortality and long-term adverse events compared with medical management only [5]. There is still ongoing debate on how revascularization in multivessel disease should be approached. Multivessel PCI was associated with higher in-hospital mortality rates (30.9 vs 28.4%, p < 0.001) and MACCE (39.9 vs 36.5%, p < 0.001) compared to single PCI [6]. However, complete revascularization in multivessel NSTEMI patients was superior compared to culprit-only in terms of reducing major adverse events and repeat revascularization [7]. The evidence on this topic in multivessel NSTE-ACS remained scarce. This study aims to analyze the outcome of different PCI approaches, between culprit-only and multivessel PCI, or single-stage versus multistage multivessel PCI in NSTE-ACS patients.

## Methods

This meta-analysis was conducted in concordance with the Preferred Reporting Items for Systematic Reviews and Meta-Analyses (PRISMA) statement [8] (Table S1**, Supplementary files**). This study was registered in PROSPERO with an identification number (CRD42023442175).

### Search strategy

A systematic search was performed in Scopus, Medline, PubMed, ScienceDirect, and Embase on July 4th, 2023. The following search keywords were used: “((unstable angina) OR (non-ST segment acute coronary syndrome) OR (non-ST segment elevation myocardial infarction) OR (NSTEMI) OR (NSTE-ACS)) AND ((revascularization) OR (PCI) OR (percutaneous coronary intervention) OR (stenting) OR (CABG) OR (coronary artery bypass graft surgery)) AND ((MVD) OR (multivessel) OR (multivessel coronary artery disease) OR (multivessel disease)) AND ((culprit-only) OR (target vessel) OR (one-stage) OR (multistage) OR (complete)). Further details of the keyword search are explained in Table S2***, Supplementary files***.

### Study selection and inclusion criteria

Studies were searched on databases, compiled, de-duplicated, and screened by three reviewers (JA, AD, and NW). Another reviewer (PS) confirmed the propriety of screened studies in case there was a discrepancy between the first three reviewers. Inclusion criteria are studies in the English language, available in full-text, involving human subjects only, and reporting the mortality of multivessel disease (MVD) NSTE-ACS patients treated with either culprit-only vs multivessel revascularization or one-stage vs multistage multivessel revascularization. Studies involving patients with diagnoses other than NSTEMI and unstable angina were excluded.

**Fig 1:**
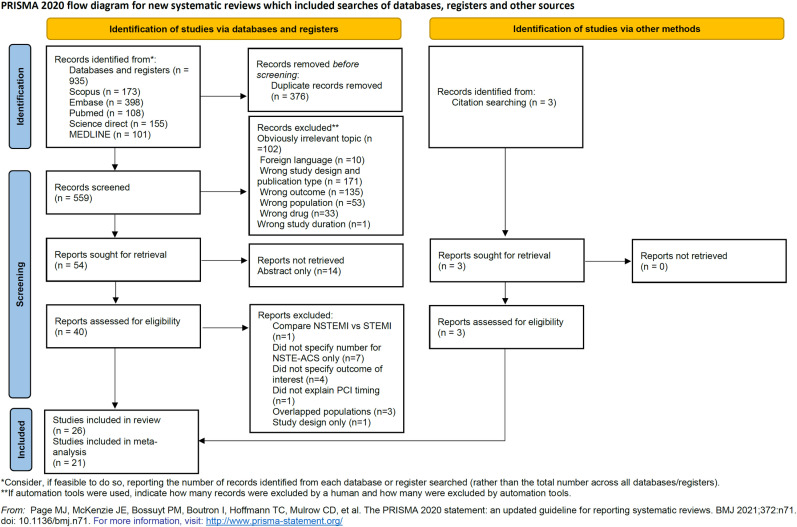
PRISMA study selection flowchart.

Studies were screened by the abstracts and then retrieved for their full text. PRISMA studies selection flowchart is displayed in Fig 1 [8]. Included studies were assessed for their bias risk using The Newcastle-Ottawa Quality Assessment Scale (NOS) for Cohort and Cross-sectional studies and the Cochrane risk-of-bias tool for randomized trials accordingly [9,10].

### Terms definition

a. Multivessel revascularization is defined as interventions performed on other lesions with different coronary artery territory in addition to culprit lesions [11].
b. Culprit-only revascularization is defined as interventions performed only on the culprit lesion, which was identified by Electrocardiography, Angiography, and regional motion abnormality on Echocardiography [12,13].
c. One-stage multivessel revascularization is defined as multivessel interventions performed in the same PCI procedure for a culprit lesion during one hospitalization, without additional PCI procedure [14].
d. Multistage multivessel revascularization is defined as multivessel interventions performed in different settings with culprit lesions [14–16].
e. Non-fatal myocardial infarction is defined as symptoms of cardiac ischemia, elevated troponin above the 99th percentile, elevated creatine kinase-MB, ECG changes such as ST segment changes, new Q waves or new left bundle branch block [14–16].
f. All repeat revascularization is defined as any repeat revascularization comprised of TLR, TVR, and non-TVR [7,16–18].
g. Target vessel revascularization (TVR) repeat is defined as any repeat revascularization performed on the same previously treated vessel [15,16,18,19].
h. Non-TVR repeat is defined as any repeat revascularization performed in different vessels from the target vessel [18].
i. Target lesion revascularization (TLR) repeat is defined as any repeat revascularization performed in the same previously treated lesion due to restenosis or re-occlusion [18].

### Data extraction and statistical analysis

Data from included studies were extracted, including authors’ names, year of publication, study designs, period of study, study center location, number of patients, age, gender, comorbidities, number of NSTE-ACS, NSTEMI and unstable angina patients, diseased and treated vessel, intervention type, in-hospital mortality, all-cause mortality, cardiac mortality, MACE, non-fatal MI, all-repeat revascularization, TVR repeat, non-TVR repeat, TLR repeat, and follow-up period. Data extraction was performed by JA, AD, and NW, then reviewed by PS in case there was a discrepancy. No missing data was encountered in the primary studies we obtained.

Statistical analysis was performed using Review Manager (version 5.4.1) by author JA. Binary outcomes were analyzed using risk ratio (RR). The pooled RR was assessed using the Mantel-Haenszel method. The heterogeneity of studies was expressed in I^2^. Random effect was used when the heterogeneity was remarkable (I^2^ =  50 – 90%) [20]. Leave-one-out sensitivity analysis was performed to evaluate the impact of each study on the heterogeneity results.

## Results

### Study selection and quality assessment

A total of 935 articles from five databases were retrieved. After excluding 376 duplicate articles, 505 articles were removed during titles and abstract screening for various reasons in Fig 1. Several studies were excluded after full-text retrieval because they did not specify the outcome of interest [21–24], did not specify the number of NSTE-ACS patients only [25–31], and overlapped populations [32–34]. Three studies were obtained from citation search [35–37]. After a full-text review, 26 studies [7,11–19,35–50] were included in the systematic review. Risk of bias assessment showed that the studies were categorized as good [7,11,12,18,19,35,36,38,45–48,50] and fair [13–17,37,39–44,49] (Table S3 ***Risk of Bias (Newcastle Ottawa Scale) and*** Table S4 ***Risk of Bias (Cochrane Risk of Bias Tools*, Supplementary files**).

### Study characteristics

This study gathered 222,350 NSTE-ACS patients from 26 included studies [7,11–19,35–50]. There were 21 studies included in the meta-analysis [7,11–13,15–19,35–38,41,42,44,45,47–50]. The included studies were conducted from 1979 – 2020. Included studies were comprised of randomized control trials [7,15,37] and cohort studies [11–14,16–19,35,36,38–50].

Most studies took place in the United States (6 studies) [11,14,42,43,46,50], followed by South Korea (2 studies) [18,35], Egypt (2 studies) [12,38], Italy (2 studies) [13,15], and the United Kingdom (2 studies) [45,48]. There were two studies involving multi-centers from several countries [36,37,49]. The characteristics of the included studies were summarized in (Table 1). NSTEMI were found in 132,550 (64.49%) and unstable angina in 72,993 patients (35.51%). Patients presented with variable comorbidities, described in (Table 2). There were 3252 and 1228 patients who received unplanned PCI and CABG, respectively [13,14,19,37,45,47,48]. Types of stents received were bare metal stent (BMS) in 17,891 patients [11,12,15,17,39,41,43,45,50], drug-eluting stents (DES) in 42,227 patients [7,11,12,15,17,39,41,43–45], and both BMS and DES in 6 patients [17] (Table 3). Balloon angioplasty was done in 31 patients [15,41]. The FFR-based approach was conducted on 30 patients [38]. Patients were followed up during their hospital stay up to 4 years post-index procedure.

**Table 1:** Characteristics of the studies.

Study	Study design	Study period	Country	Study center /database	NSTEMI (n)	Unstable angina (n)	NSTE-ACS (n)	Cardiogenic shock
Elkady, 2021	Cohort	2020	Egypt	The Kobry El-kobba Military Hospital	26	64	90	0
Alici, 2021	Cohort	2014	Türkiye	Adana Numune Training and Research Hospital	298	0	298	NA
Baumann, 2022	Cohort	2012 – 2016	Australia	CADOSA (Coronary Angiogram Database of South Australia)	3,722	0	3,722	0
Brener, 2008	Cohort	2000-2004	United States	American College of Cardiology National Cardiovascular Database Registry	40,780	70,384	111,164	1,334
Zapata, 2009	Cohort	1996 – 2006	Argentina	Instituto Cardiovascular de Rosario.	609	0	609	0
Hsieh, 2018	RCT	2005 – 2016	Taiwan	Cardiovascular Atherosclerosis and Percu-taneous TrAnsluminal INterventions (CAPTAIN)	702	0	702	28
Jarakovic, 2023	Cohort	2011 – 2017	Serbia	Institute of Cardiovascular Diseases of Vojvodina (ICVDV)	225	0	225	3
Kim, 2020	Cohort	2005 – 2015	South Korea	Korea Acute Myocardial Infarction Registry (KAMIR)	4,588	0	4,588	167
Mariani, 2001	Cohort	1997-1998	Italy	ROSAI registry	0	208	208	NA
Omer, 2021	Cohort	2009 – 2018	United States	National Cardiovascular Data Registry CathPCI Registry	25,324	0	25,324	25,324
Onuma, 2013	Cohort	2000 – 2005	United States	RESEARCH and T-SEARCH registries	NA	NA	990	NA
Pandit, 2022	Cohort	2018 – 2019	India	ABVIMS and Dr. RML Hospital, New Delhi, India	60	0	60	0
Pustjens, 2022	Cohort	2017 – 2019	Netherland	The Netherlands Heart Registration (NHR)	NA	NA	10,507	29
Rathod, 2018	Cohort	2005-2015	United Kingdom	The UK BCIS	21,587	0	21,587	0
Sadaka, 2019	Cohort	2008 – 2016	Egypt	Hospital center of Alexandria University	NA	NA	490	5
Sardella, 2016	RCT	2011 – 2013	Italy	University of Rome	527	0	527	1,177
Small, 1988	Cohort	1979-1987	United States	Mayo Clinic	0	168	168	NA
Wang, 2016	Cohort	2004 – 2008	United States	The CathPCI Registry	31,361	0	31,361	0
Yu, 2016	Cohort	2008 – 2012	China	General Hospital of Shenyang Military region, China	394	1,137	1,531	0
Shishehbor, 2007	Cohort	1995 – 2005	United States	Cleveland Clinic	NA	NA	1237	0
Lee, 2011	Cohort	2003 – 2006	South Korea	Samsung Medical Center	NA	NA	366	0
Bauer, 2013	Cohort	2005 – 2008	33 ESC countries	176 centres (Euro Heart Survey PCI-Registry)	888	1032	1920	0
Hassanin, 2015	RCT	2004	Austria, The Netherlands, Australia, New Zealand, Belgium, Norway, Canada, Spain, Denmark, Sweden, Finland, Switzerland, France, United Kingdom, Germany, United States, and Italy	ACUITY trial	NA	NA	2864	NA
Quadri, 2017	Cohort	2003 – 2014	Germany, the Netherlands, Poland, Spain, Italy, Greece, Japan, China, Canada and Brazil	BleeMACS project	1459	0	1459	NA
Correia, 2018	Cohort	2010-2013	Portugal	Braga Hospital	NA	NA	202	NA
Palmer, 2004	Cohort	2000-2001	United Kingdom	NA	NA	NA	151	0
Total					132,550	72,993	222,350	28,067

**Table 2:** Patients’ comorbidities.

Study	Diabetes Mellitus		HT		Smoking		Previous stroke		Kidney disease		Dyslipidemia		Previous MI		Previous PCI		Previous CABG		Previous HF		PAD	
	CO	MV	CO	MV	CO	MV	CO	MV	CO	MV	CO	MV	CO	MV	CO	MV	CO	MV	CO	MV	CO	MV
Ahmed, 2021		39	–	55	–	61	–	–	–	–	–	–	–	–	–	–	–	–	–	–	–	–
Alici, 2021		OS: 13 MS: 56	–	OS: 39 MS: 138	–	OS: 8 MS: 38	–	–	–	–	–	–	–	–	–	–	–	–	–	–	–	–
Baumann, 2022	579	671	1361	1111	1327	1008	147	140	50	93	–	–	327	581	239	223	–	–	–	–	–	–
Zapata, 2009	90	41	262	134	125	62	–	–	15	7	251	135	109	48	69	23	–	–	–	–	–	–
Hsieh, 2018	139	143	243	240	111	107	87	56	–	–	–	–	2	6	–	–	–	–	–	–	–	–
Jarakovic, 2023	14	52	33	146	18	85	–	–	1	7	–	–	14	32	6	28	–	–	–	–	–	–
Mariani, 2001	23	13	–	–	–	–	–	–	–	–	–	–	74	18	–	–	–	–	–	–	–	–
Omer, 2021	7959	5296	13105	8176	4015	2353	–	–	1440	1119	11609	7204	6146	3454	5542	3040	4726	1606	5083	3290	3557	2210
Onuma, 2013	70	122	159	265	–	–	–	–	–	–	204	330	197	276	123	92	–	–	–	–	–	–
Pandit, 2022	5	8	13	13	11	11	–	–	–	–	13	14	–	–	–	–	–	–	–	–	–	–
Rathod, 2018	3168	4375	6690	5920	5353	6045	395	534	527	845	5687	6526	4412	6885	1791	2959	–	–	–	–	526	563
Sadaka, 2019	118	OS: 133 MS: 17	139	OS: 149 MS; 19	28	OS: 36 MS: 7	–	–	–	–	72	OS: 80 MS: 10	16	OS: 11 MS: 2	44	OS: 42 MS: 5	–	–	–	–	–	–
Sardella, 2016	–	OS: 98 MS; 104	–	OS: 193 MS: 174	–	OS: 120 MS: 107	–	–	–	–	–	–	–	OS: 71 MS: 62	–	OS: 41 MS: 44	–	–	–	–	–	–
Small, 1988	–	–	–	–	–	–	–	–	–	–	–	–	23	12	–	–	11	6	8	2	–	–
Brener, 2008	22983	OS: 10653 MS: 1701	53316	OS: 24882 MS: 3905	19669	OS: 8522 MS: 1377	8574	OS: 3754 MS: 636	4251	OS: 1725 MS: 328	49065	OS: 23064 MS: 3491	–	–	23199	OS: 9706 MS; 1748	–	–	7133	OS: 3314 MS: 593	9006	OS: 3720 MS: 620
Wang, 2016	7657	2734	–	–	–	–	3665	1267	–	–	–	–	5556	1804	6069	2004	–	–	3035	1106	3338	1074
Yu, 2016	--	OS: 308 MS: 256		OS: 561 MS 454	–	OS: 382 MS: 341	–	OS: 68 MS: 67	–	–	–	OS: 384 MS: 454	–	OS: 172 MS: 191	–	–	–	–	–	–	–	OS: 24 MS: 19

HT: hypertension; MI; myocardial infarction; PCI: percutaneous coronary intervention; CABG: coronary-artery bypass graft; HF: heart failure; PAD: peripheral artery disease; CO: culprit-only revascularization; MV: multivessel revascularization; OS; one-stage multivessel revascularization; MS: multi-stage multivessel revascularization.

Column indicated by (-) means that data were not available on the primary studies

**Table 3:** Characteristics of revascularization.

Study	Total patients	Strategy		Timing		FFR-based	Vascular access		CABG (n)	BMS (n)	DES (n)			BMS + DES	Balloon angioplasty
		Culprit-only	Multivessel	One-stage	Multi-stage		Femoral	Radial			1^st^ gen	2^nd^ gen	Total DES		
Elkady, 2021	90	0	0	30	30	30	NA	NA	0	NA	NA	NA	NA	NA	NA
Alici, 2021	298	0	0	71	227	0	298	0	0	99	0	0	199	0	0
Baumann, 2022	3,722	491	158	0	0	0	NA	NA	0	NA	NA	NA	NA	NA	NA
Brener, 2008	111,164	72,048	33,818	28,520	5,298	0	NA	NA	671	NA	NA	NA	NA	NA	NA
Zapata, 2009	609	405	204	0	0	0	NA	NA	0	669	NA	NA	160	0	18
Hsieh, 2018	702	344	358	0	0	0	NA	NA	NA	0	127	448	575	0	0
Jarakovic, 2023	225	43	0	112	70	0	64	160	NA	99	NA	NA	119	6	0
Kim, 2020	4,588	2055	2533	0	0	0	NA	NA	NA	NA	NA	NA	NA	NA	NA
Mariani, 2001	208	158	49	0	0	0	NA	NA	3	NA	NA	NA	NA	NA	NA
Omer, 2021	25,324	15,533	9,791	0	0	0	22,403	2,716	0	NA	NA	NA	NA	NA	NA
Onuma, 2013	990	379	611	0	0	0	NA	NA	0	418,986	NA	NA	571,014	NA	NA
Pandit, 2022	60	30	30	0	0	0	NA	NA	0	0	NA	NA	60	NA	NA
Pustjens, 2022	10,507	6,272	4,235	0	0	0	NA	NA	0	NA	NA	NA	NA	NA	NA
Rathod, 2018	21,857	10,120	11,737	0	0	0	15,351	6506	42	5104	NA	NA	16,753	NA	NA
Sadaka, 2019	490	211	0	249	30	0	NA	NA	0	207	NA	NA	891	NA	NA
Sardella, 2016	527	0	0	264	263	0	83	444	0	278	0	1314	1314	NA	NA
Small, 1988	168	31	52	0	0	0	NA	NA	3	NA	NA	NA	NA	NA	NA
Wang, 2016	31,361	23,344	8,017	0	0	0	NA	NA	0	9776	NA	NA	21,585	NA	NA
Yu, 2016	1,531	0	0	859	672	0	NA	NA	0	NA	NA	NA	NA	NA	NA
Shishehbor, 2007	1237	761	479	0	0	0	NA	NA	0	1240	NA	NA	0	NA	NA
Lee, 2011	366	187	179	0	0	0	NA	NA	NA	NA	309	57	NA	NA	NA
Bauer, 2013	1920	1,186	734	0	0	0	1545	361	0	NA	NA	NA	NA	NA	0
Hassanin, 2015	2864	2,255	609	0	0	0	NA	NA	56	NA	NA	NA	2391	0	
Quadri, 2017	1459	840	619	0	0	0	665	794	NA	0	NA	NA	648	0	52
Correia, 2018	202	131	71	0	0	0	10	192	1	72	NA	NA	130	0	0
Palmer, 2004	151	57	71	0	0	0	NA	NA	2	NA	NA	NA	NA	NA	NA

#### All-Cause Mortality.

**Fig 2:**
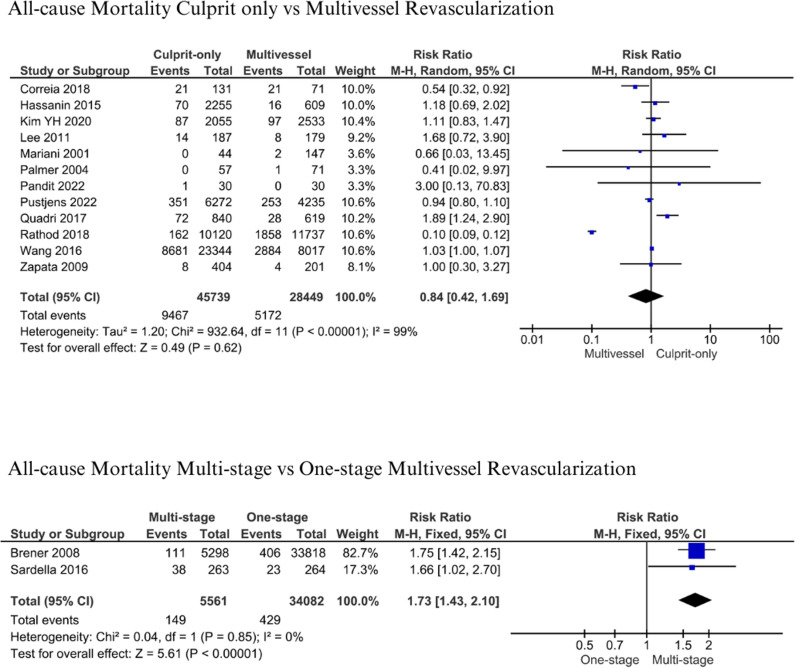
All-cause mortality for culprit-only vs. multivessel and one-stage vs multistage revascularization.

The analysis of all-cause mortality between culprit-only and multivessel revascularization included 12 studies [11,13,18,19,35,37,41,44,45,47–49], while multistage and one-stage multivessel revascularization involved two studies [14,15]. Patients were followed up six months – 4 years after the index procedure. Pooled studies gathered 45,739 with culprit-only and 28,449 patients with multivessel intervention. Our analysis yielded insignificant results and high heterogeneity (RR: 0.84; 95% CI: 0.42-1.69; p = 0.62; I^2^: 99%) (Fig 2). There were 9,467 (20.7%) vs 5,172 (18.18%) patients who died with culprit-only compared to multivessel revascularization, respectively. Therefore, there was a relatively 2.52% mortality reduction between both groups. On the other hand, analysis of all-cause mortality comparing multistage and one-stage multivessel revascularization yielded significantly higher mortality in multistage multivessel revascularization compared to one-stage with low heterogeneity (RR: 1.73; 95% CI 1.43-2.10; p < 0.01; I^2^: 0%) (Fig 2). There were 5,561 patients who had multistage and 34,082 others who had one-stage multivessel intervention. Among patients who were multivessel revascularized, 2.68% vs 1.26% of patients died with multistage vs one-stage revascularization.

#### Cardiac Mortality.

**Fig 3:**
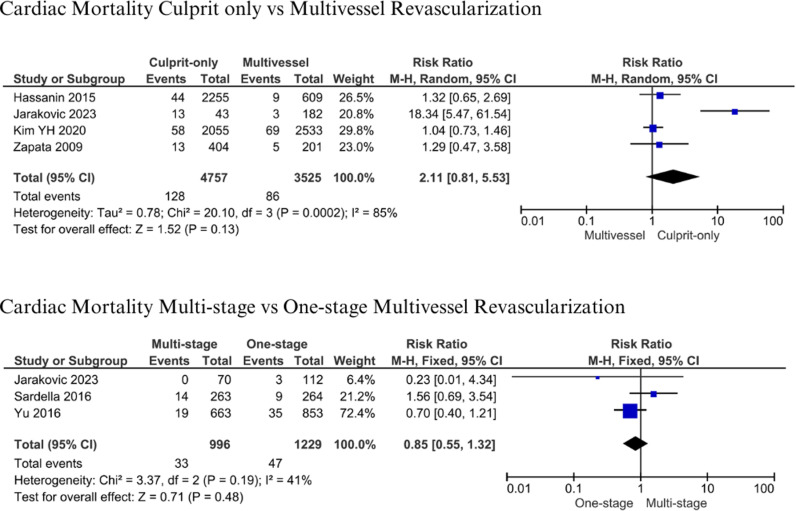
Cardiac mortality for culprit-only vs. multivessel and one-stage vs multistage revascularization.

The analysis of cardiac cause mortality of culprit only vs. multivessel revascularization included four studies [17,18,37,41]. Patients were followed up for 1 – 3 years. Included studies pooled 4,757 with culprit-only and 3,525 patients with multivessel revascularization. Studies by Zapata et al., Hassanin et al., and Jarakovic et al. showed higher cardiac mortality in culprit-only compared to multivessel revascularization [17,37,41]. Our analysis yielded no significant differences in cardiac mortality between culprit-only and multivessel revascularization (RR: 2.11; 95% CI: 0.81-5.53; p = 0.13; I^2^: 85%) (Fig 3). There were 996 patients with multistage vs 1,229 patients with one-stage multivessel revascularization. Pooled results demonstrated 128 (2.69%) vs 86 (2.44%) mortality in patients with culprit-only vs multivessel revascularization. Comparison of multistage and one-stage revascularization [15–17] revealed insignificant results (RR: 0.85; 95% CI: 0.55-1.32; p = 0.48; I^2^: 41%) during 1-year follow-up (Fig 3). Patients with one stage died 47 (3.82%) vs 33 (3.31%) compared to multistage revascularization.

#### In-hospital Mortality.

**Fig 4:**
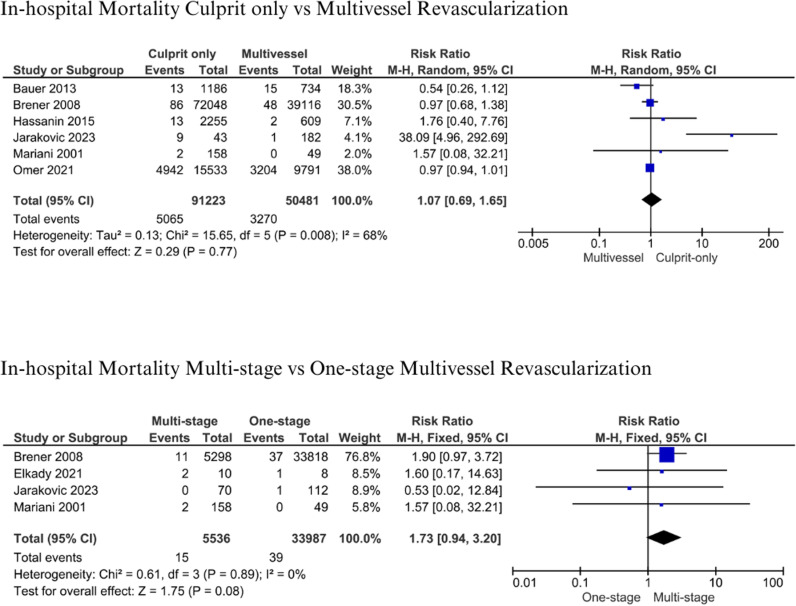
In-hospital mortality for culprit-only vs. multivessel and one-stage vs multistage revascularization.

A comparison of in-hospital mortality between culprit-only vs. multivessel revascularization [13,14,17,36,37,42] and multistage vs one-stage multivessel revascularization [13,14,17,38] was made. This analysis involved 91,223 patients from culprit-only vs 50,481 patients from multivessel revascularization. Culprit-only and multivessel revascularization demonstrated insignificant in-hospital mortality (RR: 1.07; 95% CI: 0.69-1.65; p = 0.77; I^2^: 68%) (Fig 4). Patients died in the culprit-only group at 5.55% vs 6.48% in the multivessel revascularization group. Analysis of multistage vs. one-stage multivessel revascularization involved 5,536 patients with multistage vs. 33,987 patients with one-stage multivessel intervention. It obtained higher mortality in the multistage group, although the difference is insignificant (RR: 1.73; 95% CI: 0.94-3.20; p = 0.08; I^2^: 0%) (Fig 4). There were 0.27% vs 0.11% patients who died in multistage vs one-stage multivessel revascularization, respectively.

#### Non-fatal myocardial infarction.

**Fig 5:**
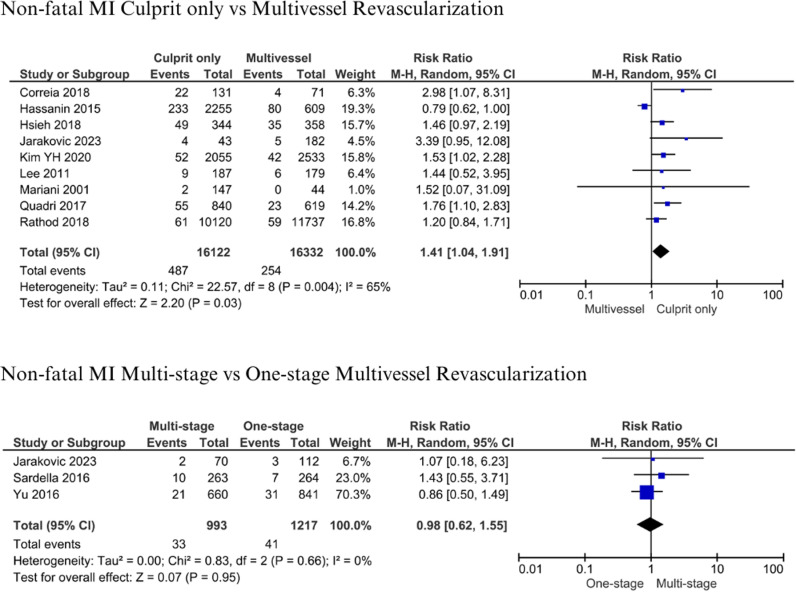
Non-fatal myocardial infarction for culprit-only vs. multivessel and one-stage vs multistage revascularization.

A total of 16,122 patients with culprit-only and 16,332 patients with multivessel revascularization from 9 studies were evaluated for non-fatal spontaneous MI [7,13,17,18,35,37,45,47,49]. Four additional studies reported non-fatal MI [19,36,44,48]. However, we did not include them in the meta-analysis due to discrepancies in the follow-up period. There were 993 patients with multistage and 1,217 with one-stage multivessel revascularization from 3 studies analyzed and evaluated for non-fatal spontaneous MI [15–17]. Patients were followed up for one year to 4 years. Culprit-only revascularization had significantly higher non-fatal spontaneous MI 487 (3.02%) vs 254 (1.56%), compared to multivessel revascularization (RR: 1.41, 95% CI: 1.04-1.91, p = 0.03, I^2^: 65%) (Fig 5). The analysis of non-fatal spontaneous MI between multistage and one-stage multivessel revascularization showed that there was no significant risk (RR 0.98, 95% CI 0.62-1.55, p = 0.95, I^2^: 99%) (Fig 5). A total of 33 (3.32%) vs 41 (3.37%) patients with multistage vs one-stage multivessel revascularization experienced non-fatal MI.

#### Major adverse cardiac events (MACE).

**Fig 6:**
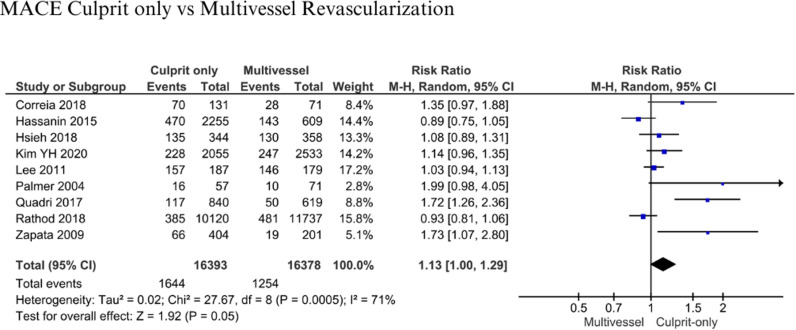
MACE for culprit-only vs multivessel revascularization.

There were 16,393 patients with culprit-only and 16,378 patients with multivessel revascularization from 9 studies. Analysis was performed for MACE difference [7,18,35,37,41,45,47–49]. Follow-up period was 1 – 3 years. The analysis showed that there was no significant risk of MACE between culprit only and multivessel revascularization (RR: 1.13, 95% CI: 1.00-1.29, p = 0.05, I^2^: 71%) (Fig 6). Patients with MACE in culprit-only were 1,644 (10.03%) compared to 1,254 (7.66%) in multivessel revascularization.

#### All Repeat Revascularization.

**Fig 7:**
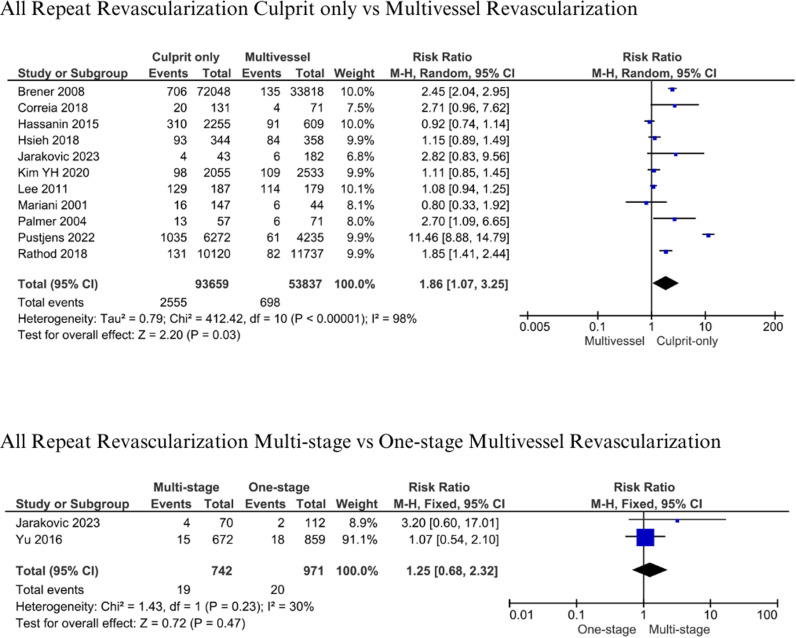
All repeat revascularization for culprit-only vs. multivessel and one-stage vs multistage revascularization.

A total of 93,659 patients with culprit-only and 53,837 with multivessel revascularization from 11 studies were evaluated in the analysis of all repeat revascularization [7,13,14,17–19,35,37,45,47,48]. In comparison, two studies were analyzed for multistage vs one-stage multivessel revascularization with 742 and 971 patients, respectively [16,17]. Follow-up period ranged from hospital stays up to 4 years. The analysis showed that there was a significant risk of all repeat revascularization between culprit only and multivessel revascularization (RR 1.86, 95% CI 1.07-3.25, p = 0.03) (Fig 7). Patients who had culprit-only revascularization had more prevalence in repeat revascularization compared to multivessel revascularization (2.73% vs 1.30%). No statistical differences were found when comparing multistage with one-stage revascularization (RR: 1.25; 95% CI: 0.68-2.32, p = 0.47, I^2^ =  30%) (Fig 7). Multistage patients had more repeat revascularization compared to one-stage multivessel revascularization (2.56% vs 2.06%).

**Fig 8:**
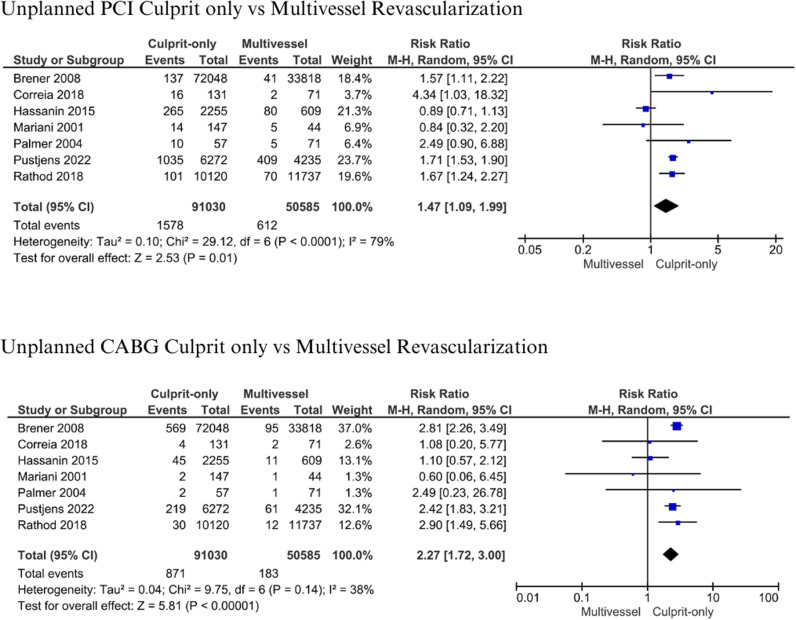
Unplanned PCI and CABG for culprit-only vs multivessel revascularization.

A total of 1,578 patients (1.73%) with culprit-only vs 612 patients (1.21%) with multivessel revascularization underwent unplanned/emergency PCI, while 871 (0.96%) vs 183 patients (0.36%) underwent unplanned/emergency CABG, in both groups, respectively [13,14,19,37,45,47,48]. Both unplanned PCI and CABG in the culprit-only group were significantly higher (RR: 1.47; 95% CI: 1.09-1.99, p = 0.01, I^2^ =  79%) and (RR: 2.27; 95% CI: 1.72-3.00, p < 0.001, I^2^ =  38%) (Fig 8).

#### TLR Repeat.

**Fig 9:**
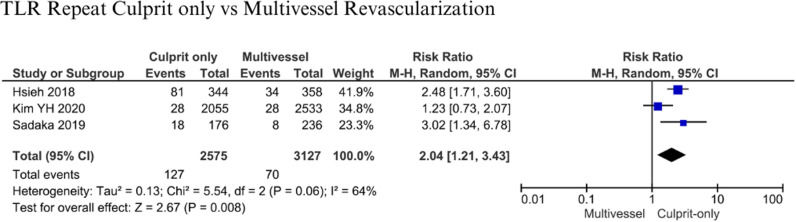
Target lesion revascularization repeat for culprit-only vs multivessel revascularization.

Three studies involved 5,702 patients in the analysis of TLR repeat [7,12,18]. Patients were followed up for six months to 3 years. Statistical analysis (RR: 2.04; 95% CI 1.21-3.43, p = 0.008; I^2^: 64%) (Fig 9) indicated that patients who had culprit-only revascularization were at about two times higher risk for TLR repeat compared to patients with multivessel revascularization.

#### TVR Repeat.

**Fig 10:**
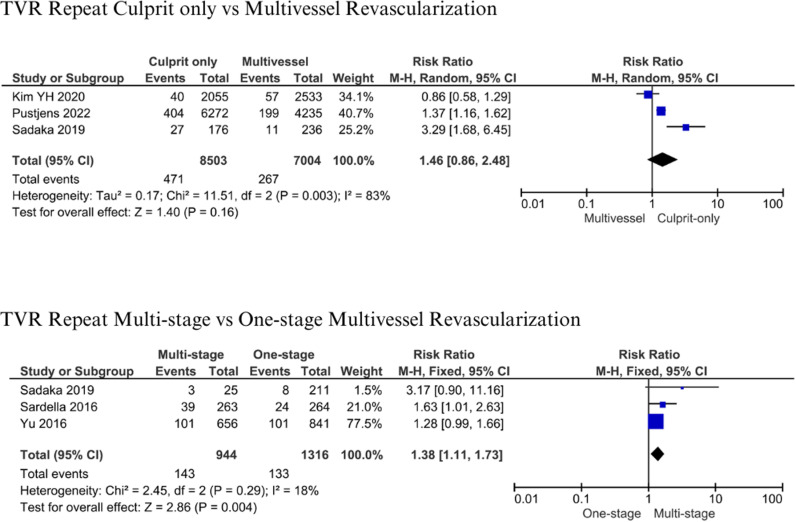
Target vessel revascularization repeat for culprit-only vs multivessel and one-stage vs multistage revascularization.

A total of 15,507 patients from 3 studies were evaluated in the analysis of TVR Repeat between culprit-only and multivessel revascularization [12,18,19]. No difference was found between culprit-only and multivessel revascularization about TVR repeat (RR: 1.46; 95% CI 0.86-2.48, p = 0.16; I^2^: 83%) (Fig 10). Three studies involving 2,260 patients were evaluated between multistage and one-stage multivessel revascularization [12,15,16]. TLR repeat was significantly 1.3 times increased by multistage revascularization compared with one-stage revascularization (RR 1.38; 95% CI 1.11-1.73; p = 0.004, I^2^: 18%) (Fig 10).

#### Non-TVR Repeat.

**Fig 11:**
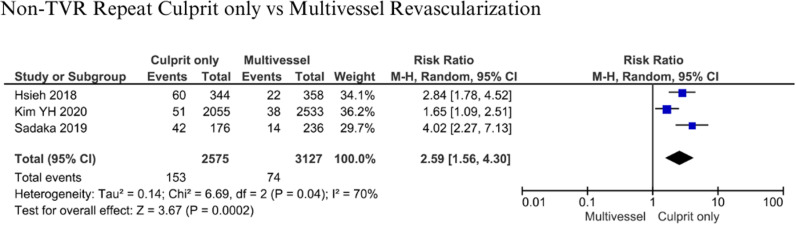
Non-target vessel revascularization repeat for culprit-only vs multivessel revascularization.

Three studies involving 5,702 patients were analyzed for non-TVR repeat [7,12,18]. The statistical analysis indicated that the patients who received culprit-only revascularization had about 2.59 times the risk for having non-TVR repeat compared to patients who received Multivessel revascularization (RR 2.59; 95% CI 1,56-4.30; p = 0.0002; I^2^: 70%) (Fig 11).

## Discussion

There was still an ongoing debate on whether lesions in MVD NSTE-ACS should be treated immediately or staged, culprit lesion only or complete, as the evidence from previous studies remained scarce. It is surprising, as half of the acute coronary syndrome patients have MVD [51,52], but the evidence is limited.

Our study indicated an insignificant difference in mortality between culprit-only and multivessel revascularization, based on all-cause, cardiac, and in-hospital mortality. It is similar to the study by Atti et al. in a meta-analysis of multivessel disease (MVD) STEMI patients comparing culprit-only and multivessel revascularization [20]. Additionally, culprit-only revascularization has significantly higher non-fatal spontaneous MI compared to multivessel revascularization. There was a 41% increase in non-fatal MI in CO compared to the MV group, in line with previous studies which reported that multivessel revascularization decreased the risk of non-fatal myocardial infarction complications [53]. Another meta-analysis also stated that the incidence of non-fatal myocardial infarction was less frequent with multivessel PCI (HR 0.64, 95% CI 0.52-0.79; p = 0.001) [54]. This occurrence is perhaps due to a pre-existing non-culprit lesion that did not undergo intervention, then progressed and caused re-infarction later after index PCI [55]. This process could be prevented by multivessel revascularization. Data from SWEDEHEART revealed that previously unstented lesions bear twice the risk of re-infarction than stented lesions [55]. Non-culprit lesions may be unstable and have similar morphology as culprit lesions [56,57]. Thus, it may benefit from stenting.

On the other hand, a significant increase in all-cause mortality was found in multistage (MS) compared to one-stage (OS) multivessel revascularization. There was a 58% increase in mortality of MS compared to OS revascularization. MS revascularization showed less favorable outcomes, probably due to prolonged procedure duration, higher contrast volume used, and longer myocardial ischemia duration [18]. Additionally, a higher incidence of MACE was also found in multistage multivessel revascularization [18], which supports the complications and contributes to the all-cause mortality incidence.

Our study showed a significant difference in the incidence of repeat revascularization between the MV and CO groups. There was an 86% increase in all repeat revascularization, 47% increase in unplanned PCI, 127% increase in unplanned CABG, 104% increase in TLR repeat, and 159% increase in non-TVR repeat with CO compared to the MV group. A study by Lee et al. revealed that MV reduced the incidence of revascularization after three years of follow-up compared to CO [35]. A study by Pustjens revealed that there was a reduced incidence of re-intervention in patients undergoing MV. These results were persistent when all revascularization electives within three months were excluded (10.2% in the MV-PCI vs. 16.2% in the CO-PCI group; p <  0.001) [19]. These results are in line with those reported in studies such as SMILE Trial by Sardella et al. (2016) [15] and by Tamburino et al. (2008) [58], van den Brand et al. (2002) [59], Bourassa (1998) [29], Ijsselmuiden et al. (2004) [60] and Nikolsky et al., (2004) [61]. Potential advantages of MV compared to CO are reduction of the myocardial area at risk and improvement of myocardial function by increasing blood flow to the peri-infarction area [47]. While culprit-only intervention could improve blood flow on the culprit lesion, other lesions remained intact and progressed over time. Residual coronary plaque lesions create a turbulent flow and reduce shear stress in the surrounding area, which induces plaque formation [62] and leads to repeat revascularization. Therefore, multivessel revascularization offers more benefits than culprit-only.

The limitation of our study is the lack of a trial study as a reference because the currently available studies remain scarce. Differences in the follow-up time between studies were present. Patients with cardiogenic shock were included in the studies. The first and second generations of drug-eluting stents were used in several studies, while the other only used newer generations. Inherent bias is commonly found in the meta-analysis.

NSTEMI patients dominated the population of this study and this is in line with data from large registry [63]. More importantly, whether NSTE-ACS presents with unstable angina or NSTEMI, they prompt similar management based on the current European Society of Cardiology (ESC) guidelines [64]. Therefore, the outcomes of our study are considered representative and relevant with entire NSTE-ACS population. Although BMS were still used in several studies despite the advancement of DES, real-world data demonstrated that BMS remained as the main modality in the intervention [65,66], especially in the developing countries [12,17,39,41]. Thus, the outcomes of this study are relevant with current practice of revascularization.

## Conclusion

This study found a more favorable outcome of multivessel compared to culprit-only revascularization. Single-staged multivessel intervention may be more beneficial than multi-staged. Careful considerations in deciding which interventions to use are important in ensuring the optimal outcome for the patients. Further studies on the current topic are encouraged.

## Supporting information

S1 table PRISMA checklist. (DOCX)

S2 table search strategy. (DOCX)

S3 table Risk of Bias (Newcastle Ottawa Scale). (DOCX)

S4 table Risk of Bias (Cochrane Risk of Bias Tools). (DOCX)

S5 table Raw data. (XLSX)

S6 Funnel plot of the analysis. (DOCX)
