# Supplementary material for: A riddle of culprit only vs multivessel or immediate vs staged revascularization in patients with non-ST elevation acute coronary syndrome: A meta-analysis
Source: PLoS One. 2025 Mar 18;20(3):e0310695. doi: 10.1371/journal.pone.0310695 (PMC11918328; doi:10.1371/journal.pone.0310695)
Supplement: S2 table — (DOCX) [file pone.0310695.s002.docx]

# S2 Search strategy

1. **Literature search keywords**

#1 ((unstable angina) OR (non-ST segment acute coronary syndrome) OR (non-ST segment elevation myocardial infarction) OR (NSTEMI) OR (NSTE-ACS))

#2 ((revascularization) OR (PCI) OR (percutaneous coronary intervention) OR (stenting) OR (CABG) OR (coronary artery bypass graft surgery))

#3 ((MVD) OR (multivessel) OR (multivessel coronary artery disease) OR (multivessel disease))

#4 ((culprit-only) OR (target vessel) OR (one-stage) OR (multi-stage) OR (complete))

#5 ((Unstable Angina [MeSH Terms]) OR (Non-ST Elevated Myocardial Infarction [MeSH Terms]))

| **Database** | **Keywords** | **Search Results** | **Search Time** |
| --- | --- | --- | --- |
| Pubmed | #1 AND #2 AND #3 AND #4 | 108 | July 4, 2023  (16:13, GMT+7) |
| Scopus | #1 AND #2 AND #3 AND #4 | 173 | July 4, 2023  (16:15, GMT+7) |
| Medline | #1 AND #2 AND #3 AND #4 | 101 | July 4, 2023  (16:19, GMT+7) |
| ScienceDirect | #3 AND #5 | 155 | July 4, 2023  (17:13, GMT+7) |
| Embase | #1 AND #2 AND #3 AND #4 | 398 | July 4, 2023  (17:25, GMT+7) |

1. **Numbered table of studies identified in the literature search**

| **Number** | **Authors** | **Include or exclude** | **Literatures** |
| --- | --- | --- | --- |
| 1 | Agra, 2021 | Excluded: did not specify outcome of interest | Agra-Bermejo, R., Cordero, A., Veloso, P. R., Álvarez, D. I., Álvarez, B. Á., Díaz, B., Rodríguez, L. A., Abou-Jokh, C., Álvarez, B. C., González-Juanatey, J. R., & García Acuña, J. M. (2021). Long term prognostic benefit of complete revascularization in elderly presenting with NSTEMI: Real world evidence. Reviews in Cardiovascular Medicine, 22(2), 475–482. https://doi.org/10.31083/j.rcm2202054 |
| 2 | Alici, 2021 | Included | Alici, G., Quisi, A., Genç, Ö., Harbalioğlu, H., Allahverdiyev, S., Yildirim, A., Urgun, Ö. D., & Gür, M. (2021). Mortality outcomes of single-staged versus multi-staged complete coronary revascularization in multivessel non-ST elevation myocardial infarction patients. Duzce Medical Journal, 23(2), 129–136. https://doi.org/10.18678/dtfd.868952 |
| 3 | Bainey, 2020 | Excluded: did not specify NSTE-ACS population only | Bainey, K. R., Alemayehu, W., Armstrong, P. W., Westerhout, C. M., Kaul, P., & Welsh, R. C. (2020). Long-Term Outcomes of Complete Revascularization With Percutaneous Coronary Intervention in Acute Coronary Syndromes. JACC: Cardiovascular Interventions, 13(13), 1557–1567. https://doi.org/10.1016/j.jcin.2020.04.034 |
| 4 | Bauer, 2013 | Included | Bauer, T., Zeymer, U., Hochadel, M., Möllmann, H., Weidinger, F., Zahn, R., Nef, H. M., Hamm, C. W., Marco, J., & Gitt, A. K. (2013). Prima-vista multi-vessel percutaneous coronary intervention in haemodynamically stable patients with acute coronary syndromes: Analysis of over 4.400 patients in the EHS-PCI registry. International Journal of Cardiology, 166(3), 596–600. https://doi.org/10.1016/j.ijcard.2011.11.024 |
| 5 | Baumann, 2020 | Excluded: did not explain PCI timing | Baumann, A. A. W., Mishra, A., Worthley, M. I., Nelson, A. J., & Psaltis, P. J. (2020). Management of multivessel coronary artery disease in patients with non-ST-elevation myocardial infarction: a complex path to precision medicine. In Therapeutic Advances in Chronic Disease (Vol. 11). SAGE Publications Ltd. https://doi.org/10.1177/2040622320938527 |
| 6 | Baumann, 2022 | Included | Baumann, A. A. W., Tavella, R., Air, T. M., Mishra, A., Montarello, N. J., Arstall, M., Zeitz, C., Worthley, M. I., Beltrame, J. F., & Psaltis, P. J. (2022). Prevalence and real-world management of NSTEMI with multivessel disease. Cardiovascular Diagnosis and Therapy, 12(1), 1–11. <https://doi.org/10.21037/cdt-21-518> |
| 7 | Biscaglia, 2021 | Excluded: abstract only | Biscaglia, S, Serenelli, M, Solé, A. et al. TCT-33 Complete Versus Culprit-Only Strategy in Older Patients With MI and Multivessel Disease: Results From a Cohort Study Based on 4 International Registries. JACC. 2021 Nov, 78 (19_Supplement_S) B13. https://doi.org/10.1016/j.jacc.2021.09.883 |
| 8 | Bourassa, 1998 | Excluded: did not specify NSTE-ACS population only | Bourassa, M. G., Yeh, W., Holubkov, R., Sopko, G., & Detre, K. M. (1998). Long-term outcome of patients with incomplete vs complete revascularization after multivessel PTCA A report from the NHLBI PTCA Registry for the investigators of the NHLBI PTCA Registry. In European Heart Journal (Vol. 19). |
| 9 | Brener, 2008 | Included | Brener, S. J., Milford-Beland, S., Roe, M. T., Bhatt, D. L., Weintraub, W. S., & Brindis, R. G. (2008). Culprit-only or multivessel revascularization in patients with acute coronary syndromes. An American College of Cardiology National Cardiovascular Database Registry report. American Heart Journal, 155(1), 140–146. https://doi.org/10.1016/j.ahj.2007.09.007 |
| 10 | Buszman, 2013 | Excluded: abstract only | Buszman, P. P., Bochenek, A., Gierlotka, M., Gasior, M., Kiesz, R. S., Milewski, K. P., Wojakowski, W., Zembala, M., Polonski, L., & Pawel, B. E. (2013). TCT-248 Immediate Stenting in Comparison with Surgical Revascularization Strategy in Patients with Non-ST Elevation Acute Coronary Syndrome and Multivessel Coronary Artery Disease - The MILESTONE Registry. Journal of the American College of Cardiology, 62(18), B81–B82. https://doi.org/10.1016/j.jacc.2013.08.983 |
| 11 | Chang, 2016 | Excluded: did not specify NSTE-ACS population only | Chang, M., Ahn, J. M., Kim, N., Lee, P. H., Roh, J. H., Yoon, S. H., Kang, S. J., Lee, S. W., Kim, Y. H., Lee, C. W., Park, S. W., Park, D. W., & Park, S. J. (2016). Complete versus incomplete revascularization in patients with multivessel coronary artery disease treated with drug-eluting stents. American Heart Journal, 179, 157–165. https://doi.org/10.1016/j.ahj.2016.06.020 |
| 12 | Correia, 2018 | Included | Correia, C., Galvão Braga, C., Martins, J., Arantes, C., Abreu, G., Quina, C., Salgado, A., Álvares Pereira, M., Costa, J., & Marques, J. (2018). Multivessel vs. culprit-only revascularization in patients with non-ST-elevation acute coronary syndromes and multivessel coronary disease. Revista Portuguesa de Cardiologia (English Edition), 37(2), 143–154. https://doi.org/10.1016/J.REPCE.2017.05.011 |
| 13 | Diletti, 2023 | Excluded: did not specify NSTE-ACS population only | Diletti, R., den Dekker, W. K., Bennett, J., Schotborgh, C. E., van der Schaaf, R., Sabaté, M., Moreno, R., Ameloot, K., van Bommel, R., Forlani, D., van Reet, B., Esposito, G., Dirksen, M. T., Ruifrok, W. P. T., Everaert, B. R. C., van Mieghem, C., Elscot, J. J., Cummins, P., Lenzen, M., … van Mieghem, N. M. (2023). Immediate versus staged complete revascularisation in patients presenting with acute coronary syndrome and multivessel coronary disease (BIOVASC): a prospective, open-label, non-inferiority, randomised trial. The Lancet, 401(10383), 1172–1182. https://doi.org/https://doi.org/10.1016/S0140-6736(23)00351-3 |
| 14 | Elkady, 2021 | Included | Elkady, A. O., Abdelghany, M., Diab, R., Ezz, A., & Elagha, A. A. (2021). Total versus staged versus functional revascularization in NSTEACS patients with multivessel disease. Egyptian Heart Journal, 73(1). https://doi.org/10.1186/s43044-021-00179-0 |
| 15 | Fagel, 2019 | Excluded: did not specify NSTE-ACS population only | Fagel, N. D., van Nooijen, F. C., Maarse, M., Slagboom, T., Herrman, J. P., van der Schaaf, R. J., Amoroso, G., Patterson, M. S., Laarman, G. J., Suttorp, M. J., & Vink, M. A. (2019). Five-year results of the complete versus culprit vessel percutaneous coronary intervention in multivessel disease using drug-eluting stents II (CORRECT II) study: a prospective, randomised controlled trial. Netherlands Heart Journal, 27(6), 310–320. https://doi.org/10.1007/s12471-019-1252-3 |
| 16 | Fernandes, 2015 | Excluded: abstract only | Acute Cardiovascular Care 2015, European Heart Journal. Acute Cardiovascular Care, Volume 4, Issue 1_suppl, 1 October 2015, Pages 5–348, https://doi.org/10.1177/2048872615599730 |
| 17 | Ferreira, 2019 | Excluded: abstract only | Ferreira, J., Monteiro, S., Baptista, R., Goncalves, F., Monteiro, P., & Goncalves, L. (n.d.). Complete versus culprit-vessel only revascularization for patients with acute coronary syndromes and multivessel disease. https://academic.oup.com/eurheartj/article-abstract/40/Supplement_1/ehz745.0197/5597093 |
| 18 | Hassanin, 2015 | Included | Hassanin, A., Brener, S. J., Lansky, A. J., Xu, K., & Stone, G. W. (2015). Prognostic impact of multivessel versus culprit vessel only percutaneous intervention for patients with multivessel coronary artery disease presenting with acute coronary syndrome. EuroIntervention, 11(3), 293–300. https://doi.org/10.4244/EIJY14M08_05 |
| 19 | Hannan, 2013 | Excluded: did not specify NSTE-ACS population only | Hannan, E. L., Samadashvili, Z., Walford, G., Jacobs, A. K., Stamato, N. J., Venditti, F. J., Holmes, D. R., Sharma, S., & King, S. B. (2013). Staged versus one-time complete revascularization with percutaneous coronary intervention for multivessel coronary artery disease patients without ST-elevation myocardial infarction. Circulation: Cardiovascular Interventions, 6(1), 12–20. https://doi.org/10.1161/CIRCINTERVENTIONS.112.974485 |
| 20 | Hawranek, 2015 | Excluded: did not specify outcome of interest | Hawranek, M., Gąsior, P., Buchta, P., Gierlotka, M., Czapla, K., Tajstra, M., Pyka, Ł., Lekston, A., Poloński, L., & Gąsior, M. (2015). Complete revascularization with percutaneous coronary intervention improves long-term outcomes in patients with multivessel coronary artery disease presenting with NSTE-ACS. Polish Heart Journal (Kardiologia Polska), 73(7), 511–519. https://doi.org/{} |
| 21 | Hawranek, 2018 | Excluded: did not specify outcome of interest | Hawranek, M., Desperak, P., Gąsior, P., Desperak, A., Lekston, A., & Gąsior, M. (2018). Early and long-term outcomes of complete revascularization with percutaneous coronary intervention in patients with multivessel coronary artery disease presenting with non-ST-segment elevation acute coronary syndromes. Postepy w Kardiologii Interwencyjnej, 14(1), 32–41. https://doi.org/10.5114/aic.2018.74353 |
| 22 | Hsieh, 2019 | Included | Hsieh, M. J., Chen, C. C., Lee, C. H., Wang, C. Y., Chang, S. H., Chen, D. Y., Yang, C. H., Tsai, M. L., Yeh, J. K., Ho, M. Y., & Hsieh, I. C. (2019). Complete and incomplete revascularization in non-ST segment myocardial infarction with multivessel disease: long-term outcomes of first- and second-generation drug-eluting stents. Heart and Vessels, 34(2), 251–258. https://doi.org/10.1007/s00380-018-1252-z |
| 23 | Huckaby, 2020 | Excluded: did not specify outcome of interest | Huckaby, L. v., Sultan, I., Mulukutla, S., Kliner, D., Gleason, T. G., Wang, Y., Thoma, F., & Kilic, A. (2020). Revascularization following non-ST elevation myocardial infarction in multivessel coronary disease. Journal of Cardiac Surgery, 35(6), 1195–1201. https://doi.org/10.1111/jocs.14539 |
| 24 | Ibrahim, 2017 | Excluded: compare NSTEMI vs STEMI | Ibrahim, H., Sharma, P. K., Cohen, D. J., Fonarow, G. C., Kaltenbach, L. A., Effron, M. B., Zettler, M. E., Peterson, E. D., & Wang, T. Y. (n.d.). Multivessel Versus Culprit Vessel-Only Percutaneous Coronary Intervention Among Patients With Acute Myocardial Infarction: Insights From the TRANSLATE-ACS Observational Study. https://doi.org/10.1161/JAHA.117 |
| 25 | Jarakovic, 2023 | Included | Jarakovic, M., Petrovic, M., Ivanisevic, D., Mihajlovic, B., Kovacevic, M., & Popov, I. (2023). Complete versus culprit only revascularization in non-ST-segment elevation myocardial infarction and multivessel coronary artery disease. Srpski Arhiv Za Celokupno Lekarstvo, 00, 2–2. https://doi.org/10.2298/sarh220210002j |
| 26 | Jia, 2019 | Excluded: abstract only | Jia, S., Liu, Y., Yao, Y., Yang, Y. J., Gao, R. L., Xu, B., & Yuan, J. Q. (n.d.). Long-term outcomes of culprit lesion only versus multi-vessel one-stage intervention in non-ST elevation acute coronary syndrome patients undergoing percutaneous coronary interventioo. https://academic.oup.com/eurheartj/article/40/Supplement_1/ehz745.0980/5595919 |
| 27 | Kim YH, 2020 | Included | Kim, Y. H., Her, A. Y., Jeong, M. H., Kim, B. K., Hong, S. J., Kim, S., Ahn, C. M., Kim, J. S., Ko, Y. G., Choi, D., Hong, M. K., & Jang, Y. (2020). Culprit-only versus multivessel or complete versus incomplete revascularization in patients with non-ST-segment elevation myocardial infarction and multivessel disease who underwent successful percutaneous coronary intervention using newer-generation drug-eluting stents. Atherosclerosis, 301, 54–64. https://doi.org/10.1016/j.atherosclerosis.2020.04.002 |
| 28 | Kim MC, 2020 | Excluded: overlapped data | Kim, M. C., Hyun, J. Y., Ahn, Y., Bae, S., Hyun, D. Y., Cho, K. H., Sim, D. S., Hong, Y. J., Kim, J. H., Jeong, M. H., Kim, H. S., Gwon, H. C., Seong, I. W., Hwang, K. K., Chae, S. C., Hur, S. H., Cha, K. S., & Oh, S. K. (2020). Optimal revascularization strategy in non–st-segment–elevation myocardial infarction with multivessel coronary artery disease: Culprit-only versus one-stage versus multistage revascularization. Journal of the American Heart Association, 9(15). https://doi.org/10.1161/JAHA.120.016575 |
| 29 | Kim, 2019 | Excluded: abstract only | "M Kim, Y Ahn, M H Jeong, D S Sim, Y J Hong, J H Kim, T H Ahn, K B Seung, H S Kim, H C Gwon, S C Chae, S H Hur, K S Cha, P3127, Optimal revascularization strategy in non-ST-segment elevation myocardial infarction with multivessel coronary artery disease: staged vs. one-time vs. culprit-only revascularization, European Heart Journal, Volume 40, Issue Supplement_1, October 2019, ehz745.0202, https://doi.org/10.1093/eurheartj/ehz745.0202" |
| 30 | Kim, 2017 | Excluded: abstract only | Kim, H. O., Kim, W., Kim, S.-J., Woo, J. S., Chung, H., & Kim, J. M. (2017). TCTAP A-011 Comparison of Timing of Non-culprit Lesion Percutaneous Coronary Intervention in Myocardial Infarction Patients. Journal of the American College of Cardiology, 69(16), S6. https://doi.org/10.1016/j.jacc.2017.03.037 |
| 31 | Kim MC, 2011 | Excluded: overlapped data | Kim, M. C., Jeong, M. H., Ahn, Y., Kim, J. H., Chae, S. C., Kim, Y. J., Hur, S. H., Seong, I. W., Hong, T. J., Choi, D. H., Cho, M. C., Kim, C. J., Seung, K. B., Chung, W. S., Jang, Y. S., Cho, S. Y., Rha, S. W., Bae, J. H., Cho, J. G., & Park, S. J. (2011). What is optimal revascularization strategy in patients with multivessel coronary artery disease in non-ST-elevation myocardial infarction? Multivessel or culprit-only revascularization. International Journal of Cardiology, 153(2), 148–153. https://doi.org/10.1016/j.ijcard.2010.08.044 |
| 32 | Lee, 2011 | Included | Lee, H. J., Song, Y. bin, Hahn, J. Y., Kim, S. M., Yang, J. H., Choi, J. H., Choi, S. H., Choi, J. H., Lee, S. H., & Gwon, H. C. (2011). Multivessel vs single-vessel revascularization in patients with non-ST-segment elevation acute coronary syndrome and multivessel disease in the drug-eluting stent era. Clinical Cardiology, 34(3), 160–165. https://doi.org/10.1002/clc.20858 |
| 33 | Mariani, 2001 | Included | Mariani, G., de Servi, S., Dellavalle, A., Repetto, S., Chierchia, S., Urbano, M. D. ’, Repetto, A., & Klersy, C. (2001). Complete or Incomplete Percutaneous Coronary Revascularization in Patients With Unstable Angina in Stent Era: Are Early and One-Year Results Different? on behalf of the ROSAI Study Group. |
| 34 | Mehran, 2021 | Excluded: abstract only | Mehran, R., Spirito, A., Cao, D., Pivato, C. A., Sartori, S., Zhang, Z., Nicolas, J., Chiarito, M., Dangas, G., Owen, R., Pocock, S., Valgimigli, M., Biscaglia, S., Serenelli, M., Solé, A. A., Sanchis, J., Giannini, F., Gallo, F., Scala, A., … Campo, G. (n.d.). TCT-32 Safety and Efficacy of Biodegradable Polymer Biolimus-Eluting Stents in Patients With Non-ST-Elevation Acute Coronary Syndrome: A Pooled Analysis of GLASSY and TWILIGHT Complete Versus Culprit-Only Strategy in Older Patients With MI and Multivessel Disease: Results From a Cohort Study Based on 4 International Registries. |
| 35 | Moore, 2017 | Excluded: abstract only | Moore, P., Chandra, N., Nadra, I., Robinson, S., Eric Fretz, Ding, L., Hardiman, S., Fung, A., Aymong, E., Chan, A., Hodge, S., Wood, D., della Siega, A., & Iqbal, B. (2017). TCT-444 The prognostic significance of complete revascularization across all indications for percutaneous coronary intervention: an analysis of 28,632 patients with multivessel disease from the British Columbia Cardiac Registry. Journal of the American College of Cardiology, 70(18), B182. https://doi.org/10.1016/j.jacc.2017.09.551 |
| 36 | Omer, 2021 | Included | Omer, M. A., Brilakis, E. S., Kennedy, K. F., Alkhouli, M., Elgendy, I. Y., Chan, P. S., & Spertus, J. A. (2021). Multivessel Versus Culprit-Vessel Percutaneous Coronary Intervention in Patients With Non–ST-Segment Elevation Myocardial Infarction and Cardiogenic Shock. JACC: Cardiovascular Interventions, 14(10), 1067–1078. https://doi.org/10.1016/j.jcin.2021.02.021 |
| 37 | Onuma, 2013 | Included | Onuma, Y., Muramatsu, T., Girasis, C., Kukreja, N., Garcia-Garcia, H. M., Daemen, J., Gonzalo, N., Piazza, N., Einthoven, J., van Domburg, R., & Serruys, P. W. (2013). Single-vessel or multivessel PCI in patients with multivessel disease presenting with non-ST-elevation acute coronary syndromes. EuroIntervention, 9(8), 916–922. https://doi.org/10.4244/EIJV9I8A154 |
| 38 | Palmer, 2004 | Included | N.D. Palmer, M. J. P. C. M. D. R. R. M. R. A. P. M. (2008). Effect of Completeness of Revascularization on Clinical Outcome in Patients with Multivessel Disease Presenting with Unstable An. Journal of Invasive Cardiology, 16(4). https://www.hmpgloballearningnetwork.com/site/jic/articles/effect-completeness-revascularization-clinical-outcome-patients-multivessel-disease |
| 39 | Pandit, 2022 | Included | Pandit, N., Rahatekar, P., Rekwal, L., Kuber, D., Nath, R. K., & Aggarwal, P. (2022). Target Vessel Versus Complete Revascularization in Non-ST Elevation Myocardial Infarction Without Cardiogenic Shock. Cureus. https://doi.org/10.7759/cureus.23139 |
| 40 | Pustjens, 2019 | Excluded: study design only | Pustjens, T. F. S., Streukens, B., Vainer, J., Gho, B., Ruiters, A. W., Stein, M., Ilhan, M., Veenstra, L., Theunissen, R., Bekkers, S. C. A. M., van’t Hof, A. W. J., & Rasoul, S. (2020). Design and rationale of ischaemia-driven complete revascularisation versus usual care in patients with non-ST-elevation myocardial infarction and multivessel coronary disease: the South Limburg Myocardial Infarction (SLIM) trial. Netherlands Heart Journal, 28(2), 75–80. https://doi.org/10.1007/s12471-019-01332-w |
| 41 | Pustjens, 2022 | Included | Pustjens, T. F. S., Timmermans, M. J. C., Rasoul, S., & van ‘t Hof, A. W. J. (2022). Multivessel versus Culprit-Only Percutaneous Coronary Intervention in Patients with Non-ST-Elevation Acute Coronary Syndrome. Journal of Clinical Medicine, 11(20). https://doi.org/10.3390/jcm11206144 |
| 42 | Quadri, 2016 | Excluded: abstract only | Monday 29 August 2016, European Heart Journal, Volume 37, Issue suppl_1, August 2016, Pages 599–983, https://doi.org/10.1093/eurheartj/ehw433 |
| 43 | Quadri, 2017 | Included | Quadri, G., Fabrizio D’ascenzo, ;, Moretti, C., Maurizio D’amico, ;, Raposeiras-Roubín, S., Abu-Assi, E., Paulo, J., Henriques, S., Saucedo, J., Ramón González-Juanatey, J., Wilton, S. B., Kikkert, J., Nuñez-Gil, I., Ariza-Sole, A., Song, ; Xiantao, Alexopoulos, D., Liebetrau, C., Kawaji, T., Huczek, Z., … Gaita, F. (2017). Complete or incomplete coronary revascularisation in patients with myocardial infarction and multivessel disease: a propensity score analysis from the “real-life” BleeMACS (Bleeding complications in a Multicenter registry of patients discharged with diagnosis of Acute Coronary Syndrome) registry. EuroIntervention, 13, 407–414. https://doi.org/10.4244/EIJ-D-16_00350 |
| 44 | Rathod, 2017 | Excluded: abstract only | Rathod, K., Koganti, S., Jain, A., Knight, C., Mathur, A., Sirker, A., O’Mahony, C., Wragg, A., & Jones, D. (2017). TCT-5 Complete versus Culprit only lesion intervention in ACS Patients with multi-vessel disease: Incidence and outcomes from The London Heart Attack Group. Journal of the American College of Cardiology, 70(18), B2–B3. https://doi.org/10.1016/j.jacc.2017.09.044 |
| 45 | Rathod, 2018a | Excluded: abstract only | Rathod. (2018). Culprit lesion versus multi-vessel intervention in patients with cardiogenic shock complicating myocardial infarction: Incidence and outcomes from The London Heart Attack Group. European Heart Journal: Acute Cardiovascular Care, 7(1_suppl), 4–361. https://doi.org/10.1177/2048872617751067 |
| 46 | Rathod, 2018b | Included | Rathod, K. S., Koganti, S., Jain, A. K., Astroulakis, Z., Lim, P., Rakhit, R., Kalra, S. S., Dalby, M. C., O’Mahony, C., Malik, I. S., Knight, C. J., Mathur, A., Redwood, S., Sirker, A., MacCarthy, P. A., Smith, E. J., Wragg, A., & Jones, D. A. (2018). Complete Versus Culprit-Only Lesion Intervention in Patients With Acute Coronary Syndromes. *Journal of the American College of Cardiology*, *72*(17), 1989–1999. https://doi.org/10.1016/j.jacc.2018.07.089 |
| 47 | Sadaka, 2019 | Included | A Sadaka, M., W Ayad, S., Saleb, E., & Zaki, A. (2019). Outcomes of different patterns of percutaneous revascularization for non-ST-segment elevation acute coronary syndrome patients with multivessel coronary artery disease. *Journal of Integrative Cardiology*, *5*(1). https://doi.org/10.15761/jic.1000268 |
| 48 | Sardella, 2016 | Included | Sardella, G., Lucisano, L., Garbo, R., Pennacchi, M., Cavallo, E., Stio, R. E., Calcagno, S., Ugo, F., Boccuzzi, G., Fedele, F., & Mancone, M. (2016). Single-Staged Compared With Multi-Staged PCI in Multivessel NSTEMI Patients: The SMILE Trial. *Journal of the American College of Cardiology*, *67*(3), 264–272. https://doi.org/10.1016/j.jacc.2015.10.082 |
| 49 | Shishehbor, 2007 | Included | Shishehbor, M. H., Lauer, M. S., Singh, I. M., Chew, D. P., Karha, J., Brener, S. J., Moliterno, D. J., Ellis, S. G., Topol, E. J., & Bhatt, D. L. (2007). In Unstable Angina or Non-ST-Segment Acute Coronary Syndrome, Should Patients With Multivessel Coronary Artery Disease Undergo Multivessel or Culprit-Only Stenting? *Journal of the American College of Cardiology*, *49*(8), 849–854. https://doi.org/10.1016/j.jacc.2006.10.054 |
| 50 | Small, 1988 | Included | Small, R. S., Holmes, D. R., Vlietstra, R. E., Reeder, G. S., Bresnahan, J. F., & Bresnahan, D. R. (1988). *Comparison of Complete and Incomplete Revascularization by Coronary Angioplasty for Unstable Angina*. |
| 51 | Vogel, 2017 | Excluded: abstract only | Vogel, B., Baber, U., Sartori, S., Chandrasekhar, J., Farhan, S., Faggioni, M., Sorrentino, S., Kini, A., Weintraub, W., Rao, S., Kapadia, S., Weiss, S., Strauss, C., Toma, C., Muhlestein, J., DeFranco, A., Effron, M., Keller, S., Baker, B., … Mehran, R. (2017). MULTIVESSEL VERSUS CULPRIT-ONLY PCI IN PATIENTS WITH NON-ST SEGMENT ELEVATION MYOCARDIAL INFARCTION AND MULTIVESSEL DISEASE: RESULTS FROM THE PROMETHEUS STUDY. *Journal of the American College of Cardiology*, *69*(11), 1148. https://doi.org/10.1016/s0735-1097(17)34537-0 |
| 52 | Vergara, 2021 | Excluded: did not specify NSTE-ACS population only | Vergara, R., Vignini, E., Ciabatti, M., Migliorini, A., Valenti, R., & Antoniucci, D. (2021). Long-Term Mortality Comparison of Patients With Acute Myocardial Infarction Complicated by Cardiogenic Shock and Treated With Culprit-Only or Multivessel Percutaneous Coronary Intervention. *Cardiovascular Revascularization Medicine*, *22*, 10–15. https://doi.org/10.1016/j.carrev.2020.06.021 |
| 53 | Wang, 2011 | Excluded: abstract only | Wang, T., Kaltenbach, L. A., Bhatt, D. L., Rao, S. v., Roe, M. T., Resnic, F. S., Cavender, M. A., Messenger, J. C., & Peterson, E. D. (2011). LONG-TERM MORTALITY ASSOCIATED WITH MULTIVESSEL VERSUS CULPRIT VESSEL ONLY PERCUTANEOUS CORONARY INTERVENTION FOR PATIENTS WITH ACUTE MYOCARDIAL INFARCTION: INSIGHTS FROM THE NATIONAL CARDIOVASCULAR CATHPCI DATA REGISTRY. *Journal of the American College of Cardiology*, *57*(14), E914. https://doi.org/10.1016/s0735-1097(11)60914-5 |
| 54 | Wang, 2016 | Included | Wang, T. Y., McCoy, L. A., Bhatt, D. L., Rao, S. v., Roe, M. T., Resnic, F. S., Cavender, M. A., Messenger, J. C., & Peterson, E. D. (2016). Multivessel vs culprit-only percutaneous coronary intervention among patients 65 years or older with acute myocardial infarction. *American Heart Journal*, *172*, 9–18. https://doi.org/10.1016/j.ahj.2015.10.017 |
| 55 | Yu, 2016a | Excluded: overlapped data | Yu, X. F., Li, Y., Wang, Q. C., Wang, X. Z., Liang, M., Zhao, X., Xu, K., & Han, Y. L. (2016). Staged versus “one-time” multivessel intervention in elderly patients with non-ST-elevation acute coronary syndrome. *Journal of Geriatric Cardiology*, *13*(9), 760–767. https://doi.org/10.11909/j.issn.1671-5411.2016.09.004 |
| 56 | Yu, 2016b | Included | Yu, X., Li, Y., Wang, Q., Liang, M., Xu, K., & Han, Y. (2016). One-time versus staged multivessel intervention in intermediate to very high-risk patients with non-ST-segment elevation acute coronary syndromes. *Korean Circulation Journal*, *46*(6), 774–783. https://doi.org/10.4070/kcj.2016.46.6.774 |
| 57 | Zapata, 2009 | Included | [Zapata, G. O., Lasave, L. I., Kozak, F., Damonte, A., Meiriño, A., Rossi, M., Carbó, S., Pollice, A., Paolasso, E., & Picabea, E. (2009). Culprit-only or multivessel percutaneous coronary stenting in patients with non-ST-segment elevation acute coronary syndromes: One-year follow-up. Journal of Interventional Cardiology, 22(4), 329–335. https://doi.org/10.1111/j.1540-8183.2009.00477.x](https://doi.org/10.1111/j.1540-8183.2009.00477.x) |
| 58 | Holmes, 1989 | Excluded: wrong study design dan publication type | Holmes DR, Vlietstra RE. Balloon angioplasty in acute and chronic coronary artery disease. *JAMA* 1989; 261: 2109–15. |
| 59 | Ando, 2017 | Excluded: wrong study design dan publication type | Ando, T., Takagi, H., & Grines, C. L. (2017). Complete versus incomplete revascularization with drug-eluting stents for multi-vessel disease in stable, unstable angina or non-ST-segment elevation myocardial infarction: A meta-analysis. *Journal of Interventional Cardiology*, *30*(4), 309–317. https://doi.org/10.1111/joic.12390 |
| 60 | Malik, 2020 | Excluded: wrong study design dan publication type | Malik, A. H., Athar, A., Zaid, S., Yandrapalli, S., Shetty, S., Malik, S., Aronow, W. S., & Ahmad, H. (2020). Culprit-Only vs Multi-Vessel Percutaneous Coronary Intervention in Patients with NSTE-ACS - A Meta-Analysis of Randomized Controlled Trials. *Cardiovascular Revascularization Medicine : Including Molecular Interventions*, *21*(6), 804–806. |
| 61 | Henriques, 2016 | Excluded: wrong study design dan publication type | Henriques, J. P. S., & Claessen, B. E. (2016). A SMILE and a Frown: One-Stage or Multistage PCI in NSTEMI Patients With Multivessel Disease. *Journal of the American College of Cardiology*, *67*(3), 273–274. https://doi.org/10.1016/j.jacc.2015.10.064 |
| 62 | Saito, 2019 | Excluded: wrong study design dan publication type | Saito, Y., & Kobayashi, Y. (2019). Percutaneous coronary intervention strategies in patients with acute myocardial infarction and multivessel disease: Completeness, timing, lesion assessment, and patient status. *Journal of Cardiology*, *74*(2), 95–101. https://doi.org/10.1016/j.jjcc.2019.04.001 |
| 63 | Hill, 2004 | Excluded: wrong study design dan publication type | Hill, R., Bagust, A., Bakhai, A., Dickson, R., Dündar, Y., Haycox, A., Mujica Mota, R., Reaney, A., Roberts, D., Williamson, P., & Walley, T. (2004). Coronary artery stents: a rapid systematic review and economic evaluation. *Health Technology Assessment (Winchester, England)*, *8*(35), iii–iv, 1–242. https://doi.org/10.3310/hta8350 |
| 64 | Moretti, 2015 | Excluded: wrong outcome | Moretti, C., Meynet, I., D’Ascenzo, F., Omedè, P., Barbero, U., Quadri, G., Ballocca, F., Zoccai, G. B., & Gaita, F. (2015). Sixty-day readmission rate after percutaneous coronary intervention: predictors and impact on long-term outcomes. *European Heart Journal. Quality of Care & Clinical Outcomes*, *1*(2), 79–84. https://doi.org/10.1093/ehjqcco/qcv001 |
| 65 | Wanha, 2017 | Excluded: wrong outcome | Wanha, W., Kawecki, D., Roleder, T., Pluta, A., Marcinkiewicz, K., Morawiec, B., Kret, M., Pawlowski, T., Smolka, G., Ochala, A., & Wojakowski, W. (2017). Gender differences and bleeding complications after PCI on first and second generation DES. *Scandinavian Cardiovascular Journal : SCJ*, *51*(1), 53–60. https://doi.org/10.1080/14017431.2016.1219044 |
| 66 | Silber, 2005 | Excluded: wrong study design dan publication type | Silber, S., Albertsson, P., Avilés, F. F., Camici, P. G., Colombo, A., Hamm, C., Jørgensen, E., Marco, J., Nordrehaug, J.-E., Ruzyllo, W., Urban, P., Stone, G. W., Wijns, W., & Task Force for Percutaneous Coronary Interventions of the European Society of Cardiology. (2005). Guidelines for percutaneous coronary interventions. The Task Force for Percutaneous Coronary Interventions of the European Society of Cardiology. *European Heart Journal*, *26*(8), 804–847. https://doi.org/10.1093/eurheartj/ehi138 |
| 67 | Almendro, 2021 | Excluded: wrong outcome | Almendro-Delia, M., Seoane García, T., Villar Calle, P., García González, N., Lorenzo López, B., Cortés, F. J., García Del Río, M., Ruiz García, M. D. P., Hidalgo Urbano, R. J., & García-Rubira, J. C. (2021). Prevalence and clinical significance of totally occluded infarct-related arteries in patients with non-ST-segment elevation acute coronary syndromes. *International Journal of Cardiology*, *324*, 1–7. https://doi.org/10.1016/j.ijcard.2020.09.040 |
| 68 | Ugo, 2006 | Excluded: foreign language | Ugo, F., Solinas, E., & Ardissino, D. (2006). [Multivessel percutaneous coronary interventions. When incomplete revascularization may represent a therapeutic option]. *Giornale Italiano Di Cardiologia (2006)*, *7*(4 Suppl 1), 36S-46S. |
| 69 | Cartier, 1999 | Excluded: wrong drug | [Cartier, R. (1999). Systematic off-pump coronary artery revascularization: experience of 275 cases. The Annals of Thoracic Surgery, 68(4), 1494–1497. https://doi.org/10.1016/s0003-4975(99)00948-0](https://doi.org/10.1016/s0003-4975(99)00948-0) |
| 70 | Li, 2023 | Excluded: wrong population | Li, Z., Zhou, Z., Guo, L., Zhong, L., Xiao, J., Meng, S., Wang, Y., Ding, H., Zhang, B., Zhu, H., Zhou, X., & Huang, R. (2023). Effect of complete percutaneous revascularization on improving long-term outcomes of patients with chronic total occlusion and multi-vessel disease. *Chinese Medical Journal*, *136*(8), 959–966. https://doi.org/10.1097/CM9.0000000000002653 |
| 71 | Vaitkus, 1995 | Excluded: wrong study design dan publication type | [Vaitkus, P. T. (1995). The continuing evolution of percutaneous transluminal coronary angioplasty in the treatment of coronary artery disease. Coronary Artery Disease, 6(5), 429–439. https://doi.org/10.1097/00019501-199505000-00011](https://doi.org/10.1097/00019501-199505000-00011) |
| 72 | Kim, 2021 | Excluded: wrong outcome | [Kim, Y. H., Her, A.-Y., Jeong, M. H., Kim, B.-K., Hong, S.-J., Lee, S.-J., Ahn, C.-M., Kim, J.-S., Ko, Y.-G., Choi, D., Hong, M.-K., & Jang, Y. (2021). Outcomes of Different Reperfusion Strategies of Multivessel Disease Undergoing Newer-Generation Drug-Eluting Stent Implantation in Patients with Non-ST-Elevation Myocardial Infarction and Chronic Kidney Disease. Journal of Clinical Medicine, 10(20). https://doi.org/10.3390/jcm10204629](https://doi.org/10.3390/jcm10204629) |
| 73 | Fukotomi, 2019 | Excluded: wrong outcome | Fukutomi, M., Toriumi, S., Ogoyama, Y., Oba, Y., Takahashi, M., Funayama, H., & Kario, K. (2019). Outcome of staged percutaneous coronary intervention within two weeks from admission in patients with ST-segment elevation myocardial infarction with multivessel disease. *Catheterization and Cardiovascular Interventions : Official Journal of the Society for Cardiac Angiography & Interventions*, *93*(5), E262–E268. https://doi.org/10.1002/ccd.27896 |
| 74 | Lee, 2020 | Excluded: wrong outcome | Lee, Y.-J., Hong, S.-J., Kim, B.-K., Shin, S., Suh, Y., Kim, S., Ahn, C.-M., Kim, J.-S., Ko, Y.-G., Choi, D., Hong, M.-K., & Jang, Y. (2020). Long-term outcomes after percutaneous coronary intervention relative to bypass surgery in diabetic patients with multivessel coronary artery disease according to clinical presentation. *Coronary Artery Disease*, *31*(2), 174–183. https://doi.org/10.1097/MCA.0000000000000767 |
| 75 | Teplitsky, 2003 | Excluded: wrong outcome | Teplitsky, I., Assali, A., Golovchiner, G., Shor, N., Weiss, A., Battler, A., & Kornowski, R. (2003). Acute and intermediate-term results of percutaneous coronary stenting in octogenarian patients. *International Journal of Cardiovascular Interventions*, *5*(4), 195–199. https://doi.org/10.1080/14628840310018167 |
| 76 | Lallemant, 1992 | Excluded: foreign language | Lallemant, R., Bauters, C., Leroy, F., Lablanche, J. M., & Bertrand, M. E. (1992). [Coronary angioplasty of multivessel coronary diseases. Apropos of 1664 procedures. Immediate results and results following 6 months]. *Archives Des Maladies Du Coeur et Des Vaisseaux*, *85*(6), 815–822. |
| 77 | Voudris, 2004 | Excluded: wrong drug | Voudris, V. A., Skoularigis, J. S., Dimitriou, Y. K., Grapsa, G. N., Malakos, J. S., Pavlides, G. S., Manginas, A. N., & Cokkinos, D. V. (2004). Diabetes mellitus and unstable coronary artery disease: improved clinical outcome of coronary artery stenting in an era of glycoprotein IIb/IIIa inhibitors and lipid-lowering therapy. *Coronary Artery Disease*, *15*(6), 353–359. https://doi.org/10.1097/00019501-200409000-00009 |
| 78 | Rodriguezm 2007 | Excluded: wrong outcome | Rodriguez, A. E., Maree, A. O., Mieres, J., Berrocal, D., Grinfeld, L., Fernandez-Pereira, C., Curotto, V., Rodriguez-Granillo, A., O’Neill, W., & Palacios, I. F. (2007). Late loss of early benefit from drug-eluting stents when compared with bare-metal stents and coronary artery bypass surgery: 3 years follow-up of the ERACI III registry. *European Heart Journal*, *28*(17), 2118–2125. https://doi.org/10.1093/eurheartj/ehm297 |
| 79 | Breeman, 2022 | Excluded: wrong drug | Breeman, A., Mercado, N., Lenzen, M., van den Brand, M. M. J., Harrington, R. A., Califf, R. M., Topol, E. J., Simoons, M. L., Boersma, E., & PURSUIT Investigators. (2002). Characteristics, treatment and outcome of patients with non-ST-elevation acute coronary syndromes and multivessel coronary artery disease: observations from PURSUIT (platelet glycoprotein IIb/IIIa in unstable angina: receptor suppression using integrelin therapy). *Cardiology*, *98*(4), 195–201. https://doi.org/10.1159/000067321 |
| 80 | Pomozova, 2019 | Excluded: foreign language | Pomozova, T. P., Lykov, Y. V, Komarova, I. S., Dyatlov, N. V, & Zhelnov, V. V. (2019). [Clinical and laboratory features of primary acute myocardial infarction in patients with obstructive and non-obstructive coronary atherosclerosis]. *Kardiologiia*, *59*(10S), 41–51. https://doi.org/10.18087/cardio.2640 |
| 81 | Breeman, 2001 | Excluded: wrong outcome | Breeman, A., Boersma, E., van den Brand, M. J., van Herwerden, L., & Serruys, P. W. (2001). Completeness of revascularisation by percutaneous coronary intervention. *Netherlands Heart Journal : Monthly Journal of the Netherlands Society of Cardiology and the Netherlands Heart Foundation*, *9*(1), 3–9. |
| 82 | Tanaka, 2002 | Excluded: irrelevant topic | Tanaka, H., Narisawa, T., Mori, N., Masuda, M., Kishi, D., Suzuki, T., & Takaba, T. (2002). The left internal thoracic artery and radial artery composite graft in off-pump coronary artery bypass grafting. *Annals of Thoracic and Cardiovascular Surgery : Official Journal of the Association of Thoracic and Cardiovascular Surgeons of Asia*, *8*(4), 204–208. |
| 83 | Lettieri, 2005 | Excluded: foreign language | Lettieri, C., Buffoli, F., Romano, M., Aroldi, M., Baccaglioni, N., Tomasi, L., Rosiello, R., Agostini, F., Kuwornu, H., Pepi, P., Izzo, A., & Zanini, R. (2005). [Percutaneous coronary revascularization in patients over eighty: acute and long-term results]. *Italian Heart Journal. Supplement : Official Journal of the Italian Federation of Cardiology*, *6*(9), 588–598. |
| 84 | Ribichini, 2007 | Excluded: irrelevant topic | Ribichini, F., Tomai, F., Paloscia, L., Di Sciascio, G., Carosio, G., Romano, M., Verna, E., Galli, M., Tamburino, C., De Cesare, N., Pirisi, R., Piscione, F., Lanteri, G., Ferrero, V., Vassanelli, C., & DESIRE investigators. (2007). Steroid-eluting stents in patients with acute coronary syndrome: the dexamethasone eluting stent Italian registry. *Heart (British Cardiac Society)*, *93*(5), 598–600. https://doi.org/10.1136/hrt.2006.098467 |
| 85 | Kaul, 1999 | Excluded: wrong outcome | Kaul, T. K., Fields, B. L., Riggins, L. S., Wyatt, D. A., & Jones, C. R. (1999). Reinterventions for recurrent ischemic heart disease following a successful first re-do myocardial revascularization: predictors, indications and results. *Cardiovascular Surgery (London, England)*, *7*(3), 363–368. https://doi.org/10.1016/s0967-2109(98)00158-6Kaul |
| 86 | Cecchi, 2008 | Excluded: irrelevant topic | Cecchi, E., Liotta, A. A., Gori, A. M., Valente, S., Giglioli, C., Lazzeri, C., Sofi, F., Gensini, G. F., Abbate, R., & Mannini, L. (2008). Comparison of Hemorheological Variables in ST-Elevation Myocardial Infarction Versus Those in Non-ST-Elevation Myocardial Infarction or Unstable Angina Pectoris. *The American Journal of Cardiology*, *102*(2), 125–128. https://doi.org/10.1016/j.amjcard.2008.03.026 |
| 87 | Murakami, 1992 | Excluded: wrong outcome | [Murakami, T., Kino, K., Kioka, Y., Arai, S., Kurozumi, K., Nakayama, Y., Indoh, S., Senoo, Y., & Teramoto, S. (1992). Multivessel coronary revascularization with bilateral internal thoracic artery grafts. Surgery Today, 22(1), 10–14. https://doi.org/10.1007/BF00326118](https://doi.org/10.1007/BF00326118) |
| 88 | AGEMA, 2004 | Excluded: wrong outcome | AGEMA, W. (2004). Current PTCA practice and clinical outcomes in The Netherlands: the real world in the pre-drug-eluting stent era. *European Heart Journal*, *25*(13), 1163–1170. https://doi.org/10.1016/j.ehj.2004.05.006 |
| 89 | Bouchard, 1998 | Excluded: wrong drug | Bouchard, D. (1998). Off-pump revascularization of multivessel coronary artery disease has a decreased myocardial infarction rate. *European Journal of Cardio-Thoracic Surgery*, *14*, 20–24. https://doi.org/10.1016/S1010-7940(98)00099-2 |
| 90 | Casas, 1985 | Excluded: wrong outcome | Casas, L., Locicero, J., Sanders, J. H., & Michaelis, L. L. (1985). *Complete Surgical Revascularization: The lTeatment of Choice in Young Patients with Multivessel Coronary Disease*. |
| 91 | Sheiban, 2000 | Excluded: wrong outcome | Sheiban, I., Leonardo, F., Rosano, G. M., Pagnotta, P., Marsico, F., Montorfano, M., Di Mario, C., Trevi, G., & Colombo, A. (2000). Predictors of long-term clinical outcome in patients undergoing multiple vessel stenting for coronary artery disease. *Italian Heart Journal : Official Journal of the Italian Federation of Cardiology*, *1*(7), 480–486. |
| 92 | Wańha, 2016 | Excluded: irrelevant topic | Wańha, W., Kawecki, D., Roleder, T., Pluta, A., Marcinkiewicz, K., Dola, J., Morawiec, B., Krzych, Ł., Pawłowski, T., Smolka, G., Ochała, A., Nowalany-Kozielska, E., Tendera, M., & Wojakowski, W. (2016). Impact of anaemia on long-term outcomes in patients treated with first- and second-generation drug-eluting stents; Katowice-Zabrze Registry. *Kardiologia Polska*, *74*(6), 561–569. https://doi.org/10.5603/KP.a2015.0217 |
| 93 | Ben, 2013 | Excluded: wrong outcome | Ben, Ahmed H., I. Hamdi, T. Elkateb, Hassan F. Ben, A. Mokaddem, Ameur Y. Ben, and M. R. Boujnah. 2013. “[Benefits of Coronary Revascularization in Septuagenarian Patients with Acute Coronary Syndrome].” *La Tunisie Medicale* 91 (8-9). https://pubmed.ncbi.nlm.nih.gov/24227513/. |
| 94 | Frutos, 1991 | Excluded: wrong outcome | Frutos, Rangel E., García R. García, Valadez E. Fernández, Sedano J. Zúñiga, Bazavilvazo S. Verduzco, Zamorano R. Siordia, and García y. Otero Jm. 1991. “[Coronary Angioplasty of Multiple Vessels and Lesions in Unstable Functional Class-IV Angina].” *Archivos Del Instituto de Cardiologia de Mexico* 61 (4). https://pubmed.ncbi.nlm.nih.gov/1953209/. |
| 95 | Breisblatt, 1991 | Excluded: wrong outcome | Breisblatt, W. M., Ruffner, R. J., Uretsky, B. F., & Reddy, P. S. (1991). Same-Day Angioplasty and Diagnostic Catheterization: Safe and Effective but Riskier in Unstable Angina. *Angiology*, *42*(8), 607–613. https://doi.org/10.1177/000331979104200802 |
| 96 | Le Feuvre, 2010 | Excluded: irrelevant topic | Le Feuvre, C., Healy-Brucker, A., Helft, G., Monségu, J., Varenne, O., Spaulding, C., Collet, J. P., Beygui, F., Barthélémy, O., Choussat, R., Montalescot, G., & Metzger, J. P. (2010). Long-term follow-up of patients with sirolimus-eluting stents for treatment of bare-metal in-stent restenosis. *International Journal of Cardiology*, *140*(2), 219–225. https://doi.org/10.1016/j.ijcard.2008.11.029 |
| 97 | Scott, 1994 | Excluded: wrong outcome | Scott, N. A., Kelsey, S. F., Detre, K., Cowley, M., King, S. B., & The NHLBI PTCA Registry Investigators. (1994). Percutaneous transluminal coronary angioplasty in African-American patients (The National Heart, Lung, and Blood Institute 1985–1986 Percutaneous Transluminal Coronary Angioplasty Registry). *The American Journal of Cardiology*, *73*(16), 1141–1146. https://doi.org/10.1016/0002-9149(94)90171-6 |
| 98 | Daemen, 2006 | Excluded: irrelevant topic | Daemen, J., Ong, A. T. L., Stefanini, G. G., Tsuchida, K., Spindler, H., Sianos, G., de Jaegere, P. P. T., van Domburg, R. T., & Serruys, P. W. (2006). Three-Year Clinical Follow-Up of the Unrestricted Use of Sirolimus-Eluting Stents as Part of the Rapamycin-Eluting Stent Evaluated at Rotterdam Cardiology Hospital (RESEARCH) Registry. *The American Journal of Cardiology*, *98*(7), 895–901. https://doi.org/10.1016/j.amjcard.2006.04.031 |
| 99 | Lied, 2022 | Excluded: wrong outcome | Lied, A., T. H. Morstøl, and H. Vik-Mo. 2002. “[Coronary Angioplasty in Elderly Patients].” *Tidsskrift for Den Norske Laegeforening: Tidsskrift for Praktisk Medicin, Ny Raekke* 122 (13). https://pubmed.ncbi.nlm.nih.gov/12098919/. |
| 100 | Reddy, 1998 | Excluded: wrong outcome | Reddy, N. K., Raju, P. R., Kapoor, S., Reddy, R. P., Rao, M. S., Kumar, D. N., Sastry, B. K., Prasad, S. G., & Raju, B. S. (1998). Clinical evaluation of new balloon mountable coil stent: in-hospital and follow-up results. *Indian Heart Journal*, *50*(1), 40–44. |
| 101 | Xenogiannis, 2019 | Excluded: wrong outcome | Xenogiannis, I., Tajti, P., Burke, M. N., Chavez, I., Gössl, M., Mooney, M., Poulose, A., Sorajja, P., Traverse, J., Wang, Y., & Brilakis, E. S. (2019). Coronary revascularization and use of hemodynamic support in acute coronary syndromes. *Hellenic Journal of Cardiology*, *60*(3), 165–170. https://doi.org/10.1016/j.hjc.2019.01.010 |
| 102 | Jang, 2015 | Excluded: wrong study design dan publication type | [Jang, Jae-Sik, Han-Young Jin, Jeong-Sook Seo, Tae-Hyun Yang, Dae-Kyeong Kim, Dong-Soo Kim, Kyoung-Im Cho, Bo-Hyun Kim, Yong Hyun Park, and Hyung-Gon Je. 2015. “Meta-Analysis of Multivessel versus Culprit-Only Percutaneous Coronary Intervention in Patients with Non-ST-Segment Elevation Acute Coronary Syndrome and Multivessel Coronary Disease.” The American Journal of Cardiology 115 (8): 1027–32.](http://paperpile.com/b/KSp2Lw/Q5yO) |
| 103 | Yeoh, 2017 | Excluded: wrong outcome | [Yeoh, Julian, Matias B. Yudi, Nick Andrianopoulos, Bryan P. Yan, David J. Clark, Stephen J. Duffy, Angela Brennan, et al. 2017. “Evolution of Australian Percutaneous Coronary Intervention (from the Melbourne Interventional Group [MIG] Registry).” The American Journal of Cardiology 120 (1): 47–54.](http://paperpile.com/b/KSp2Lw/R1fq) |
| 104 | [Feistritzer, 2020](https://paperpile.com/c/KSp2Lw/v70r) | Excluded: wrong study design dan publication type | [Feistritzer, Hans-Josef, Alexander Jobs, Suzanne de Waha-Thiele, Ingo Eitel, Anne Freund, Mohamed Abdel-Wahab, Steffen Desch, and Holger Thiele. 2020. “Multivessel versus Culprit-Only PCI in STEMI Patients with Multivessel Disease: Meta-Analysis of Randomized Controlled Trials.” Clinical Research in Cardiology: Official Journal of the German Cardiac Society 109 (11): 1381–91.](http://paperpile.com/b/KSp2Lw/v70r) |
| 105 | [Zimarino, 2013](https://paperpile.com/c/KSp2Lw/RYXo) | Excluded: wrong study design dan publication type | [Zimarino, Marco, Nick Curzen, Vincenzo Cicchitti, and Raffaele De Caterina. 2013. “The Adequacy of Myocardial Revascularization in Patients with Multivessel Coronary Artery Disease.” International Journal of Cardiology 168 (3): 1748–57.](http://paperpile.com/b/KSp2Lw/RYXo) |
| 106 | Sandoval, 2015 | Excluded: wrong study design dan publication type | [Sandoval, Yader, Emmanouil S. Brilakis, Mariana Canoniero, Demetris Yannopoulos, and Santiago Garcia. 2015. “Complete versus Incomplete Coronary Revascularization of Patients with Multivessel Coronary Artery Disease.” Current Treatment Options in Cardiovascular Medicine 17 (3): 366.](http://paperpile.com/b/KSp2Lw/C0Cp) |
| 107 | Lawton, 2022 | Excluded: wrong study design dan publication type | Lawton, J. S., Tamis-Holland, J. E., Bangalore, S., Bates, E. R., Beckie, T. M., Bischoff, J. M., Bittl, J. A., Cohen, M. G., DiMaio, J. M., Don, C. W., Fremes, S. E., Gaudino, M. F., Goldberger, Z. D., Grant, M. C., Jaswal, J. B., Kurlansky, P. A., Mehran, R., Metkus, T. S., Nnacheta, L. C., … Zwischenberger, B. A. (2022). 2021 ACC/AHA/SCAI Guideline for Coronary Artery Revascularization: Executive Summary: A Report of the American College of Cardiology/American Heart Association Joint Committee on Clinical Practice Guidelines. *Circulation*, *145*(3). https://doi.org/10.1161/CIR.0000000000001039 |
| 108 | Chacko, 2020 | Excluded: wrong study design dan publication type | Chacko, L., P. Howard, J., Rajkumar, C., Nowbar, A. N., Kane, C., Mahdi, D., Foley, M., Shun-Shin, M., Cole, G., Sen, S., Al-Lamee, R., Francis, D. P., & Ahmad, Y. (2020). Effects of Percutaneous Coronary Intervention on Death and Myocardial Infarction Stratified by Stable and Unstable Coronary Artery Disease. *Circulation: Cardiovascular Quality and Outcomes*, *13*(2). https://doi.org/10.1161/CIRCOUTCOMES.119.006363 |
| 109 | Benussi, 2019 | Excluded: wrong outcome | [Benussi, Bernardo, Giuseppe Gatti, Florida Gripshi, Federico Biondi, Aldostefano Porcari, Danilo Ruggiero, Manuel Belgrano, Elisabetta Rauber, Gianfranco Sinagra, and Aniello Pappalardo. 2019. “Clinical Validation of a Coronary Surgery Technique That Minimizes Aortic Manipulation.” The Annals of Thoracic Surgery 107 (4): 1166–73.](http://paperpile.com/b/KSp2Lw/W8CI) |
| 110 | Di Bacco, 2019 | Excluded: wrong population | [Di Bacco, Lorenzo, Alberto Repossini, Maurizio Tespili, Claudio Muneretto, and Gianluigi Bisleri. 2019. “Long-Term Follow-up of Total Arterial versus Conventional and Hybrid Myocardial Revascularization: A Propensity Score Matched Analysis.” Cardiovascular Revascularization Medicine: Including Molecular Interventions 20 (1): 22–28.](http://paperpile.com/b/KSp2Lw/GXsl) |
| 111 | Gaba, 2021 | Excluded: wrong study design dan publication type | [Gaba, Prakriti, Bernard J. Gersh, Ziad A. Ali, Jeffrey W. Moses, and Gregg W. Stone. 2021. “Complete versus Incomplete Coronary Revascularization: Definitions, Assessment and Outcomes.” Nature Reviews. Cardiology 18 (3): 155–68.](http://paperpile.com/b/KSp2Lw/sXcS) |
| 112 | Tung, 2014 | Excluded: wrong outcome | Tung, Y.-C. and Hsiao, P.-G. and Hsu, L.-A. and Kuo, C.-T. and Chang, C.-J. 2014.,” Comparison between exclusive and selective drug-eluting stent strategies in treating patients with multivessel coronary artery disease(Article)” *Acta Cardiologica Sinica*. 30 (3): 181-189 |
| 113 | Nishonov, 2023 | Excluded: foreign language | [Nishonov, A. B., R. S. Tarasov, S. V. Ivanov, and L. S. Barbarash. 2023. “Outcomes of Coronary Artery Bypass Grafting and Percutaneous Coronary Intervention in High-Risk Non-ST-Segment Elevation Acute Coronary Syndromes.” Complex Issues of Cardiovascular Diseases 12 (1): 151–59.](http://paperpile.com/b/KSp2Lw/mhAb) |
| 114 | Tovar, 2020 | Excluded: wrong outcome | [Tovar Forero, Maria Natalia, Thomas Zanchin, Kaneshka Masdjedi, Laurens J. C. van Zandvoort, Isabella Kardys, Felix Zijlstra, Jonas Häner, et al. 2020. “Incidence and Predictors of Outcomes after a First Definite Coronary Stent Thrombosis.” EuroIntervention: Journal of EuroPCR in Collaboration with the Working Group on Interventional Cardiology of the European Society of Cardiology 16 (4): e344–50.](http://paperpile.com/b/KSp2Lw/Zg6f) |
| 115 | Watanabe, 2022 | Excluded: wrong drug | [Watanabe, Yusuke, Tsutomu Fujita, Hirosato Doi, Tetsuya Tobaru, Shuichiro Takanashi, Yoshihisa Kinoshita, Yasuhide Okawa, et al. 2022. “Prospective Multicenter Registry of Hybrid Coronary Artery Revascularization Combined with Non-Saphenous Vein Graft Surgical Bypass and Percutaneous Coronary Intervention Using Everolimus Eluting Metallic Stents (PRIDE-METAL Study).” Cardiovascular Intervention and Therapeutics 37 (2): 304–11.](http://paperpile.com/b/KSp2Lw/X2xm) |
| 116 | Peteiro, 2020 | Excluded: irrelevant topic | [Peteiro, Jesus, and Alberto Bouzas-Mosquera. 2020. “Is There a Role for Ischemia Detection after an Acute Myocardial Infarction?” World Journal of Cardiology 12 (1): 1–6.](http://paperpile.com/b/KSp2Lw/EZhO) |
| 117 | Bellamoli, 2020 | Excluded: irrelevant topic | [Bellamoli, Michele, Federico Marin, Luca Maritan, Daniele Prati, Enrico Tadiello, Gabriele Pesarini, Giacomo Mugnai, Flavio Luciano Ribichini, and Michele Pighi. 2020. “New-Onset Extreme Right Axis Deviation in Acute Myocardial Infarction: Clinical Characteristics and Outcomes.” Journal of Electrocardiology 60 (May):60–66.](http://paperpile.com/b/KSp2Lw/6JCY) |
| 118 | [Berezhnoi, 2019](http://paperpile.com/b/KSp2Lw/U3h6) | Excluded: wrong population | [Berezhnoi, Kirill, Leonid Kokov, and Alexandr Vanyukov. 2019. “Effects of Complete Revascularization on Long-Term Treatment Outcomes in Patients with Multivessel Coronary Artery Disease over 80 Years of Age Admitted for Acute Coronary Syndrome.” Cardiovascular Diagnosis and Therapy 9 (4): 301–9.](http://paperpile.com/b/KSp2Lw/U3h6) |
| 119 | [Rigatelli, 2021](http://paperpile.com/b/KSp2Lw/zx8e) | Excluded: wrong drug | [Rigatelli, Gianluca, Marco Zuin, Filippo Gianese, Dario Adami, Fabio dell’Avvocata, Stefano Barison, Alberto Mazza, Claudio Picariello, and Loris Roncon. 2021. “Ultrathin Biodegradable-Polymer Orsiro Drug-Eluting Stent Performance in Real Practice Challenging Settings.” Cardiovascular Revascularization Medicine: Including Molecular Interventions 30 (September):12–17.](http://paperpile.com/b/KSp2Lw/zx8e) |
| 120 | Scarsini, 2020 | Excluded: irrelevant topic | [Scarsini, Roberto, Dimitrios Terentes-Printzios, Giovanni Luigi De Maria, Flavio Ribichini, and Adrian Banning. 2020. “Why, When and How Should Clinicians Use Physiology in Patients with Acute Coronary Syndromes?” Interventional Cardiology (London, England) 15 (April):e05.](http://paperpile.com/b/KSp2Lw/eNEX) |
| 121 | Gu, 2020 | Excluded: wrong study design dan publication type | [Gu, Dachuan, Jianyu Qu, Heng Zhang, and Zhe Zheng. 2020. “Revascularization for Coronary Artery Disease: Principle and Challenges.” Advances in Experimental Medicine and Biology 1177:75–100.](http://paperpile.com/b/KSp2Lw/cUcH) |
| 122 | Khan, 2020 | Excluded: wrong study design dan publication type | [Khan, Mohammad Saud, Abdur Rahman Khan, Abdullah Irfan Khan, Michael Seo, Farah Yasmin, Muhammad Shariq Usman, Abdelmoniem Moustafa, Christopher H. Schmid, Ankur Kalra, and Sohail Ikram. 2020. “Comparison of Revascularization Strategies in Patients with Acute Coronary Syndrome and Multivessel Coronary Disease: A Systematic Review and Network Meta-Analysis.” Catheterization and Cardiovascular Interventions: Official Journal of the Society for Cardiac Angiography & Interventions 96 (4): E447–54.](http://paperpile.com/b/KSp2Lw/Yh5F) |
| 123 | [Hsieh, 2013](http://paperpile.com/b/KSp2Lw/rQ4E) | Excluded: wrong population | [Hsieh, Victar, and Shamir R. Mehta. 2013. “How Should We Treat Multi-Vessel Disease in STEMI Patients?” Current Treatment Options in Cardiovascular Medicine 15 (1): 129–36.](http://paperpile.com/b/KSp2Lw/rQ4E) |
| 124 | [Elgendy, 2020](http://paperpile.com/b/KSp2Lw/B7yJ) | Excluded: wrong study design dan publication type | [Elgendy, Islam Y., Dhruv Mahtta, and David Paniagua. 2020. “Multivessel PCI for Acute Myocardial Infarction: Where Do We Stand after the COMPLETE Trial?” Current Cardiology Reports 22 (9): 97.](http://paperpile.com/b/KSp2Lw/B7yJ) |
| 125 | [Pavasini, 2022](http://paperpile.com/b/KSp2Lw/mLv6) | Excluded: wrong study design dan publication type | [Pavasini, Rita, Federico Sanguettoli, Luca Zanarelli, Maria Angela Deserio, Nicola Bianchi, Gioele Fabbri, Matteo Tebaldi, Simone Biscaglia, and Gianluca Campo. 2022. “Unsolved Questions in the Revascularization of Older Myocardial Infarction Patients with Multivessel Disease.” Reviews in Cardiovascular Medicine 23 (10): 344.](http://paperpile.com/b/KSp2Lw/mLv6) |
| 126 | Park, 2018 | Excluded: wrong drug | [Park, Keun Ho, Myung Ho Jeong, Young Joon Hong, Youngkeun Ahn, Hyun Kuk Kim, Young Yub Koh, Doo Il Kim, et al. 2018. “Effectiveness and Safety of Biolimus A9TM-Eluting stEnt in Patients with AcUTe Coronary sYndrome; A Multicenter, Observational Study (BEAUTY Study).” Yonsei Medical Journal 59 (1): 72–79.](http://paperpile.com/b/KSp2Lw/WEqC) |
| 127 | [Werner, 2020](http://paperpile.com/b/KSp2Lw/v8YZ) | Excluded: wrong study design dan publication type | [Werner, Nikos, and Franz-Josef Neumann. 2021. “Entwicklung kardiovaskuläre Medizin 2020.” Der Kardiologe 15 (2): 92–100.](http://paperpile.com/b/KSp2Lw/v8YZ) |
| 128 | Hwang, 2013 | Excluded: irrelevant topic | [Hwang, In Kyeom, Yun Kyung Kim, Seung-Woon Rha, Ji Eun Ra, Bong Soo Seo, Ji Kyoung Lee, Jin Oh Na, et al. 2013. “Impact of Insulin Resistance on 1-Year Clinical Outcomes in Non-Diabetic Patients Undergoing Percutaneous Coronary Intervention with Drug-Eluting Stents.” Journal of Cardiology 61 (2): 113–16.](http://paperpile.com/b/KSp2Lw/Ztmj) |
| 129 | Javaid, 2007 | Excluded: irrelevant topic | [Javaid, Aamir, Ashesh N. Buch, Daniel H. Steinberg, Tina Pinto Slottow, Probal Roy, Augusto D. Pichard, Lowell F. Satler, et al. 2007. “Does Creatine Kinase-MB (CK-MB) Isoenzyme Elevation Following Percutaneous Coronary Intervention with Drug-Eluting Stents Impact Late Clinical Outcome?” Catheterization and Cardiovascular Interventions: Official Journal of the Society for Cardiac Angiography & Interventions 70 (6): 826–31.](http://paperpile.com/b/KSp2Lw/DUPj) |
| 130 | Przewłocki, 2000 | Excluded: wrong outcome | Przewłocki, T. and Pieniazek, P. and Tracz, W. and Ryniewicz, W. and Olszowska, M. and Kostkiewicz, M. and Podolec, P. and Pasowicz, M. and Sokołowski, A. 2000. “Long-term results of coronary balloon angioplasty in various age groups” *Przegla̧d lekarski*. 57 (5): 266-273 |
| 131 | [De Bruyne, 2012](http://paperpile.com/b/KSp2Lw/ZCgK) | Excluded: wrong study design dan publication type | [De Bruyne, Bernard. 2012. “Multivessel Disease.” Circulation 125 (21): 2557–59.](http://paperpile.com/b/KSp2Lw/ZCgK) |
| 132 | Da̧bek, 2007 | Excluded: wrong study design dan publication type | Da̧bek, J. and Jakubowski, D. and Ga̧sior, Z. 2007. “Acute coronary syndromes in patients over 80 years old” *Polski Merkuriusz Lekarski*. 22 (132) : 514-518 |
| 133 | Davidavicius, 2005 | Excluded: wrong population | Davidavicius, Giedrius, Frank Van Praet, Samer Mansour, Filip Casselman, Jozef Bartunek, Ivan Degrieck, Francis Wellens, et al. 2005. “Hybrid Revascularization Strategy.” *Circulation* 112 (9_supplement). https://doi.org/10.1161/circulationaha.104.524264. |
| 134 | Kovacic, 2012 | Excluded: wrong drug | [Kovacic, Jason C., Paul Lee, Rucha Karajgikar, Usman Baber, Birju Narechania, Javed Suleman, Pedro R. Moreno, Samin K. Sharma, and Annapoorna S. Kini. 2012. “Safety of Temporary and Permanent Suspension of Antiplatelet Therapy after Drug Eluting Stent Implantation in Contemporary ‘Real-World’ Practice.” Journal of Interventional Cardiology 25 (5): 482–92.](http://paperpile.com/b/KSp2Lw/pTf0) |
| 135 | Huang, R.L, 2007 | Excluded: wrong study design dan publication type | Huang, R.L. 2007. “Multivessel or culprit-only stenting in patients with unstable angina or NSTEMI: Commentary” *Journal of Clinical Outcomes Management*. 14 (4): 183-184 |
| 136 | Ugo, 2006 | Excluded: wrong study design dan publication type | Ugo, F. and Solinas, E. and Ardissino, D. 2006. “Multivessel coronary revascularization. When incomplete revascularization may represent a therapeutic option” *Giornale Italiano di Cardiologia*. 7 (4): 36S-46S |
| 137 | Lallemant, 1992 | Excluded: irrelevant topic | Lallemant, R. and Bauters, C. and Leroy, F. and Lablanche, J.M. and Bertrand, M.E.1992. “Coronary angioplasty of multivessel disease: Immediate results and at 6 months in 1664 procedures” *Archives des Maladies du Coeur et des Vaisseaux*. 85 (6): 815-822 |
| 138 | Chacko, 2020 | Excluded: wrong study design dan publication type | Chacko, L., Howard, J. P., Rajkumar, C., Nowbar, A. N., Kane, C., Mahdi, D., Foley, M., Shun-Shin, M., Cole, G., Sen, S., Al-Lamee, R., Francis, D. P., & Ahmad, Y. (2020). Effects of percutaneous coronary intervention on death and myocardial infarction stratified by stable and unstable coronary artery disease: A meta-analysis of randomized controlled trials. *Circulation: Cardiovascular Quality and Outcomes*. https://doi.org/10.1161/CIRCOUTCOMES.119.006363 |
| 139 | Mahmud, 2018 | Excluded: wrong study design dan publication type | Mahmud, E., & Ben-Yehuda, O. (2018). Percutaneous Coronary Intervention in Acute Coronary Syndrome. *Journal of the American College of Cardiology*, *72*(17), 2000–2002. https://doi.org/10.1016/j.jacc.2018.08.2129 |
| 140 | Maillard, 2017 | Excluded: irrelevant topic | [Maillard, L., Tavildari, A., Barra, N., Billé, J., Joly, P., Peycher, P., Silvestri, M., & Vochelet, F. (2017). Immediate and 1-year follow-up with the novel nanosurface modified COBRA PzF stent. Archives of Cardiovascular Diseases, 110(12), 682–688. https://doi.org/10.1016/j.acvd.2017.04.010](https://doi.org/10.1016/j.acvd.2017.04.010) |
| 141 | Ochala, 2004 | Excluded: wrong outcome | Ochala, A. and Smolka, G.A. and Wojakowski, W. and Dudek, D. and Dziewierz, A. and Krolikowski, Z. and Gasior, Z. and Tendera, M. 2004. “The function of the left ventricle after complete multivessel one-stage percutaneous coronary intervention in patients with acute myocardial infarction” J*ournal of Invasive Cardiology.*16 (12): 699-702 |
| 142 | De Backer, 2019 | Excluded: wrong population | De Backer, O., Lønborg, J., Helqvist, S., Warnøe, J., Kløvgaard, L., Holmvang, L., Pedersen, F., Tilsted, H.-H., Raungaard, B., Jørgensen, E., Køber, L., Høfsten, D. E., Kelbæk, H., & Engstrøm, T. (2019). Characterisation of lesions undergoing ischaemia-driven revascularisation after complete revascularisation versus culprit lesion only in patients with STEMI and multivessel disease: a DANAMI-3-PRIMULTI substudy. *EuroIntervention*, *15*(2), 172–179. https://doi.org/10.4244/EIJ-D-18-00766 |
| 143 | Khan, 2020 | Excluded: wrong study design dan publication type | Khan, M. S., Khan, A. R., Khan, A. I., Seo, M., Yasmin, F., Usman, M. S., Moustafa, A., Schmid, C. H., Kalra, A., & Ikram, S. (2020). Comparison of revascularization strategies in patients with acute coronary syndrome and multivessel coronary disease: A systematic review and network meta‐analysis. *Catheterization and Cardiovascular Interventions*, *96*(4). https://doi.org/10.1002/ccd.28855 |
| 144 | Trabattoni, 2019 | Excluded: wrong outcome | Trabattoni, Daniela and Fabbiocchi, Franco and Montorsi, Piero and Galli, Stefano and Ravagnani, Paolo and Calligaris, Giuseppe and Teruzzi, Giovanni and Grancini, Luca and Troiano, Sarah and Ferrari, Cristina and Bartorelli, Antonio L. 2019. “A Long-Term Single-Center Registry of 6893 Patients Undergoing Elective Percutaneous Coronary Intervention With the Xience Everolimus-Eluting Stent.” *The Journal of invasive cardiology*. 31 (5): 146-151 |
| 145 | Biscaglia, 2020 | Excluded: wrong outcome | Biscaglia, S., Guiducci, V., Santarelli, A., Amat Santos, I., Fernandez-Aviles, F., Lanzilotti, V., Varbella, F., Fileti, L., Moreno, R., Giannini, F., Colaiori, I., Menozzi, M., Redondo, A., Ruozzi, M., Gutiérrez Ibañes, E., Díez Gil, J. L., Maietti, E., Biondi Zoccai, G., Escaned, J., … Campo, G. (2020). Physiology-guided revascularization versus optimal medical therapy of nonculprit lesions in elderly patients with myocardial infarction: Rationale and design of the FIRE trial. *American Heart Journal*, *229*, 100–109. https://doi.org/10.1016/j.ahj.2020.08.007 |
| 146 | Marzocchi, 1999 | Excluded: irrelevant topic | Marzocchi, A and Ortolani, P and Piovaccari, G and Marrozzini, C and Nobile, G and Palmerini, T and Marinucci, L and Saia, F and Bacchi Reggiani, M L and Branzi, A and Magnani, B. 1999. “Coronary stenting for unstable angina: predictors of 30-day and long-term clinical outcome.” *Coronary artery disease*. 10 (2) : 81-88 |
| 147 | Mahadevan, 2019 | Excluded: wrong study design dan publication type | Mahadevan, K., & Strange, J. W. (2019). Case-Based Review of the Current Global Evidence Supporting Impella-Facilitated Complex and Complete Revascularization. *JACC: Case Reports*, *1*(5), 869–872. https://doi.org/10.1016/j.jaccas.2019.11.024 |
| 148 | Nishida, 1999 | Excluded: wrong study design dan publication type | Nishida, H and Tomizawa, Y and Endo, M and Koyanagi, H. 1999. “[Complete arterial revascularization in emergency CABG].” *Kyobu geka. The Japanese journal of thoracic surgery*. 52 (8): 688-692 |
| 149 | Vereshchagin, 2018 | Excluded: irrelevant topic | Vereshchagin, I E and Ganyukov, V I and Tarasov, R S and Kochergin, N A and Shukevich, D L and Barbarash, O L. 2018. “ [Transcutaneous coronary intervention with the use of extracorporeal membrane oxygenation in patients with acute coronary syndrome].” *Angiologiia i sosudistaia khirurgiia = Angiology and vascular surgery*. 24 (4): 151-156 |
| 150 | Vandormael, 1991 | Excluded: wrong outcome | Vandormael, M., Deligonul, U., Taussig, S., & Kern, M. J. (1991). Predictors of long-term cardiac survival in patients with multivessel coronary artery disease undergoing percutaneous transluminal coronary angioplasty. *The American Journal of Cardiology*, *67*(1), 1–6. https://doi.org/10.1016/0002-9149(91)90089-4 |
| 151 | Liu, 1995 | Excluded: wrong outcome | Liu, M and Gao, W and Huo, Y. 1995. “[Percutanous transluminal coronary angioplasty for unstable angina].” *Zhonghua nei ke za zhi*. 34 (30): 169-172 |
| 152 | Casella, 2003 | Excluded: irrelevant topic | Casella, Gianni and Prati, Francesco. 2003. “Stenting small coronary arteries: The Multi-Link PIXEL Multicenter Italian Registry.” *The Journal of invasive cardiology*. 15 (7): 371-376 |
| 153 | el Gaylani, 1996 | Excluded: wrong outcome | el Gaylani, N and McAdam, B F and White, U and Gearty, G F and Walsh, M J and Crean, P A. 1996. “Immediate and follow-up results of coronary angioplasty--lessons for the future.” *Irish medical journal*. 89 (2): 60-61 |
| 154 | Le Feuvre, 2006 | Excluded: irrelevant topic | Le Feuvre, C., Montalescot, G., Rosey, G., Collet, J. P., Beygui, F., Choussat, R., Gelft, G., Monségu, J., Ohanessian, A., Spaulding, C., Drobinski, G., & Metzger, J. P. (2006). Predictive factors of cardiac events after implantation of sirolimus-eluting stents for treatment of in-stent restenosis. *International Journal of Cardiology*, *109*(2), 207–212. https://doi.org/10.1016/j.ijcard.2005.06.004 |
| 155 | Serota, 1991 | Excluded: wrong outcome | Serota, H., Deligonul, U., Lee, W.-H., Aguirre, F., Kern, M. J., Taussig, S. A., & Vandormael, M. G. (1991). Predictors of cardiac survival after percutaneous transluminal coronary angioplasty in patients with severe left ventricular dysfunction. *The American Journal of Cardiology*, *67*(5), 367–372. https://doi.org/10.1016/0002-9149(91)90043-K |
| 156 | Kiesz, 1998 | Excluded: wrong drug | Kiesz, R. S., Rozek, M. M., Mego, D. M., Patel, V., Ebersole, D. G., & J. Chilton, R. (1998). Acute directional coronary atherectomy prior to stenting in complex coronary lesions: ADAPTS study. *Catheterization and Cardiovascular Diagnosis*, *45*(2), 105–112. |
| 157 | Seabra-Gomes, 2001 | Excluded: irrelevant topic | Seabra-Gomes, R and Farto eAbreu, P and Marques, A L and Pereira, H and da Cunha, J A and Carvalho, H and Ribeiro, V G and Garcia, L M and Mourão, L and dos Santos, L P and Providência, L and Figueiredo, L and Almeida, M and Cavaco, D. 2001. “Use of stents for small coronary arteries. Results of the Multi-Link 2.5 Portuguese Registry.” *Revista portuguesa de cardiologia : orgao oficial da Sociedade Portuguesa de Cardiologia = Portuguese journal of cardiology : an official journal of the Portuguese Society of Cardiology* 20 (9): 819-837 |
| 158 | Albanesi, 1997 | Excluded: irrelevant topic | Albanesi Filho, F M and Castier, M B and da Silva, T T and Boghossian, S H and Ginefra, P. 1997. “Obstructive ischemic involvement of the anterior descending coronary artery in asymmetrical septal form of hypertrophic cardiomyopathy.” *Arquivos brasileiros de cardiologia*. 69 (5): 309-315 |
| 159 | Krishnan, 1994 | Excluded: wrong drug | Krishnan, R and Lu, J and Dae, M W and Botvinick, E H. 1994. “Does myocardial perfusion scintigraphy demonstrate clinical usefulness in patients with markedly positive exercise tests? An assessment of the method in a high-risk subset.” *American heart journal*. **127 (4): 804-816** |
| 160 | Windecker, 2006 | Excluded: wrong study design dan publication type | Windecker, S. (2006). Acute Coronary Syndromes. In *Clinical Critical Care Medicine* (pp. 301–318). Elsevier. https://linkinghub.elsevier.com/retrieve/pii/B9780323028448500354 |
| 161 | Tomey, 2014 | Excluded: foreign language | Tomey, M. I., Narula, J., & Kovacic, J. C. (2014). Advances in the Understanding of Plaque Composition and Treatment Options. *Journal of the American College of Cardiology*, *63*(16), 1604–1616. https://doi.org/10.1016/j.jacc.2014.01.042 |
| 162 | NA, 2006 | Excluded: wrong study design dan publication type | Innovation in Intervention: i2 Summit 2006. (2006). *Journal of the American College of Cardiology*, *47*(4), B1–B56. https://doi.org/10.1016/j.jacc.2006.01.023 |
| 163 | Athanasiou, 2004 | Excluded: irrelevant topic | Athanasiou, T., Al-Ruzzeh, S., Kumar, P., Crossman, M.-C., Amrani, M., Pepper, J. R., Del Stanbridge, R., Casula, R., & Glenville, B. (2004). Off-pump myocardial revascularization is associated with less incidence of stroke in elderly patients. *The Annals of Thoracic Surgery*, *77*(2), 745–753. https://doi.org/10.1016/j.athoracsur.2003.07.002 |
| 164 | AbuRahma, 2022 | Excluded: wrong population | AbuRahma, A. F., Avgerinos, E. D., Chang, R. W., Darling, R. C., Duncan, A. A., Forbes, T. L., Malas, M. B., Perler, B. A., Powell, R. J., Rockman, C. B., & Zhou, W. (2022). The Society for Vascular Surgery implementation document for management of extracranial cerebrovascular disease. *Journal of Vascular Surgery*, *75*(1), 26S-98S. https://doi.org/10.1016/j.jvs.2021.04.074 |
| 165 | Zueco, 2005 | Excluded: irrelevant topic | Zueco Gil, J. (2005). Influence of Clinical and Anatomical Factors on the Outcome of Percutaneous Coronary Interventions. *Revista Española de Cardiología (English Edition)*, *58*(4), 430–441. https://doi.org/10.1016/S1885-5857(06)60671-3 |
| 166 | Roubin, 1990 | Excluded: wrong study design dan publication type | Roubin, G. S. (1990). Status of percutaneous transluminal coronary angioplasty. *Current Problems in Cardiology*, *15*(12), 726–804. https://doi.org/10.1016/0146-2806(90)90003-9 |
| 167 | Power, 2014 | Excluded: wrong study design dan publication type | Power, B. (2014). Acute cardiac syndromes, investigations and interventions. In *Oh’s Intensive Care Manual* (pp. 167-190.e4). Elsevier. https://linkinghub.elsevier.com/retrieve/pii/B9780702047626000205 |
| 168 | Shimony, 2011 | Excluded: wrong study design dan publication type | [Shimony, Avi, Lawrence Joseph, Salvatore Mottillo, and Mark J. Eisenberg. 2011. “Coronary Artery Perforation During Percutaneous Coronary Intervention: A Systematic Review and Meta-Analysis.” The Canadian Journal of Cardiology 27 (6): 843–50.](http://paperpile.com/b/KSp2Lw/Wg9L) |
| 169 | Sahebkar, 2016 | Excluded: wrong drug | Sahebkar, Amirhossein and Giorgini, Paolo and Ludovici, Valeria and Pedone, Claudio and Ferretti, Gianna and Bacchetti, Tiziana and Grassi, Davide and Di Giosia, Paolo and Ferri, Claudio. 2016. “Impact of statin therapy on plasma resistin and visfatin concentrations: A systematic review and meta-analysis of controlled clinical trials” *Pharmacological Research*. 111: 827-837 |
| 170 | Gaudino, 2019 | Excluded: wrong outcome | Gaudino, M. F. L., Spadaccio, C., & Taggart, D. P. (2019). State-of-the-Art Coronary Artery Bypass Grafting. *Interventional Cardiology Clinics*, *8*(2), 173–198. https://doi.org/10.1016/j.iccl.2018.11.007 |
| 171 | NA, 2001 | Excluded: wrong study design dan publication type | [ACCIS2001 angiography & interventional cardiology. (2001). Journal of the American College of Cardiology, 37(2), A1–A86. https://doi.org/10.1016/S0735-1097(01)80001-2](https://doi.org/10.1016/S0735-1097(01)80001-2) |
| 172 | NA, 2006 | Excluded: wrong study design dan publication type | Special Topics. (2006). *Journal of the American College of Cardiology*, *47*(4), A251–A271. https://doi.org/10.1016/j.jacc.2006.01.016 |
| 173 | King, 1998 | Excluded: wrong study design dan publication type | King, S. B. (1998). The Development of Interventional Cardiology. *Journal of the American College of Cardiology*, *31*(4), 64B-88B. https://doi.org/10.1016/S0735-1097(97)00558-5 |
| 174 | Aulakh, 2007 | Excluded: irrelevant topic | [Aulakh, A. K., & Anand, S. S. (2007). Sex and Gender Subgroup Analyses of Randomized Trials. Women’s Health Issues, 17(6), 342–350. https://doi.org/10.1016/j.whi.2007.04.002](https://doi.org/10.1016/j.whi.2007.04.002) |
| 175 | Chokshi, 1987 | Excluded: wrong outcome | Chokshi, S. K., Meyers, S., & Abi-Mansour, P. (1987). Percutaneous transluminal coronary angioplasty: Ten year’s experience. *Progress in Cardiovascular Diseases*, *30*(3), 147–210. https://doi.org/10.1016/0033-0620(87)90012-0 |
| 176 | Ang, 2023 | Excluded: irrelevant topic | Ang, S. P., Chia, J. E., Jaiswal, V., Bandyopadhyay, D., Iglesias, J., Mohan, G. V. K., Gautam, S., Win, T., Kumar, T., Iqbal, A., Chia, T. H., & Aronow, W. (2023). Subclinical Hypothyroidism and Clinical Outcomes After Percutaneous Coronary Intervention: A Meta-Analysis. *Current Problems in Cardiology*, *48*(8), 101719. https://doi.org/10.1016/j.cpcardiol.2023.101719 |
| 177 | Sun, 2014 | Excluded: irrelevant topic | Sun, Z., & Xu, L. (2014). Computational fluid dynamics in coronary artery disease. *Computerized Medical Imaging and Graphics*, *38*(8), 651–663. https://doi.org/10.1016/j.compmedimag.2014.09.002 |
| 178 | Kern, 2004 | Excluded: wrong study design dan publication type | Kern, M. J. (2004). BASIC CORONARY BALLOON ANGIOPLASTY AND STENTING. In *Interventional Cardiac Catheterization Handbook* (pp. 11–71). Elsevier. https://linkinghub.elsevier.com/retrieve/pii/B9780323022385500085 |
| 179 | Kalavrouziotis, 2017 | Excluded: irrelevant topic | Kalavrouziotis, D., Rodés-Cabau, J., & Mohammadi, S. (2017). Moving Beyond SHOCK: New Paradigms in the Management of Acute Myocardial Infarction Complicated by Cardiogenic Shock. *Canadian Journal of Cardiology*, *33*(1), 36–43. https://doi.org/10.1016/j.cjca.2016.10.018 |
| 180 | Von Birgelen, 2018 | Excluded: irrelevant topic | Von Birgelen, C., Kok, M. M., Sattar, N., Zocca, P., Doelman, C., Kant, G. D., Löwik, M. M., Van Der Heijden, L. C., Sen, H., Van Houwelingen, K. G., Stoel, M. G., Louwerenburg, J. (Hans) W., Hartmann, M., De Man, F. H. A. F., Linssen, G. C. M., Doggen, C. J. M., & Tandjung, K. (2018). “Silent” Diabetes and Clinical Outcome After Treatment With Contemporary Drug-Eluting Stents. *JACC: Cardiovascular Interventions*, *11*(5), 448–459. https://doi.org/10.1016/j.jcin.2017.10.038 |
| 181 | Waksman, 2004 | Excluded: wrong study design dan publication type | Waksman, R. (2004). Abstracts. *Cardiovascular Radiation Medicine*, *5*(4), 177–202. https://doi.org/10.1016/j.carrev.2005.03.003 |
| 182 | NA, 2009 | Excluded: wrong study design dan publication type | [Angiography and Interventional Cardiology (i2 Summit). (2009). Journal of the American College of Cardiology, 53(10), A1–A99. https://doi.org/10.1016/j.jacc.2009.01.013](https://doi.org/10.1016/j.jacc.2009.01.013) |
| 183 | NA, 2005 | Excluded: wrong study design dan publication type | Angiography and interventional cardiology. (2005). *Journal of the American College of Cardiology*, *45*(3), A21–A90. https://doi.org/10.1016/j.jacc.2004.12.035 |
| 184 | Berk, 1999 | Excluded: wrong study design dan publication type | Berk, B. C. (1999). Abstracts of original contributions: Young investigators awards competition. *Journal of the American College of Cardiology*, *33*(2), 1A-320A. https://doi.org/10.1016/S0735-1097(99)00029-7 |
| 185 | Karha, 2007 | Excluded: irrelevant topic | Karha, J., Lincoff, A. M., & Ellis, S. G. (2007). Mechanical Approaches to Percutaneous Coronary Intervention. In *Cardiovascular Therapeutics* (pp. 121–134). Elsevier. https://linkinghub.elsevier.com/retrieve/pii/B9781416033585500127 |
| 186 | Bocchino, 2020 | Excluded: wrong study design dan publication type | [“Invasive versus Conservative Management in Spontaneous Coronary Artery Dissection: A Meta-Analysis and Meta-Regression Study.” 2021. Hellenic Journal of Cardiology: HJC = Hellenike Kardiologike Epitheorese 62 (4): 297–303.](http://paperpile.com/b/KSp2Lw/ZEcg) |
| 187 | Briasoulis, 2015 | Excluded: irrelevant topic | Briasoulis, A., Afonso, L., Palla, M., Sharma, S., Panaich, S., Papageorgiou, N., & Tousoulis, D. (2015). Culprit-vessel versus complete revascularization during primary angioplasty in ST-elevation myocardial infarction: An updated meta-analysis. *International Journal of Cardiology*, *178*, 171–174. https://doi.org/10.1016/j.ijcard.2014.10.109 |
| 188 | Ariyaratne, 2014 | Excluded: wrong study design dan publication type | Ariyaratne, T. V., Ademi, Z., Yap, C.-H., Billah, B., Rosenfeldt, F., Yan, B. P., & Reid, C. M. (2014). Prolonged effectiveness of coronary artery bypass surgery versus drug-eluting stents in diabetics with multi-vessel disease: An updated systematic review and meta-analysis. *International Journal of Cardiology*, *176*(2), 346–353. |
| 189 | NA, 2007 | Excluded: wrong study design dan publication type | Clinical Outcomes of PCI (E-poster 228-285). (2007). *The American Journal of Cardiology*, *100*(8), S96–S114. https://doi.org/10.1016/j.amjcard.2007.09.043 |
| 190 | NA, 2019 | Excluded: wrong study design dan publication type | Abstracts of the 14th Annual Scientific Meeting of the Society of Cardiovascular Computed Tomography. (2019). *Journal of Cardiovascular Computed Tomography*, *13*(3), S8–S93. https://doi.org/10.1016/j.jcct.2019.06.004 |
| 191 | Bavishi, 2017 | Excluded: wrong study design dan publication type | Bavishi, C., Sardar, P., Chatterjee, S., Khan, A. R., Shah, A., Ather, S., Lemos, P. A., Moreno, P., & Stone, G. W. (2017). Intravascular ultrasound–guided vs angiography-guided drug-eluting stent implantation in complex coronary lesions: Meta-analysis of randomized trials. *American Heart Journal*, *185*, 26–34. https://doi.org/10.1016/j.ahj.2016.10.008 |
| 192 | Bergelson, 1997 | Excluded: irrelevant topic | Bergelson, B. A., Fishman, R. F., & Tommaso, C. L. (1997). Abrupt vessel closure: Changing importance, management, and consequences. *American Heart Journal*, *134*(3), 362–381. https://doi.org/10.1016/S0002-8703(97)70069-3 |
| 193 | Kim, 2023 | Excluded: irrelevant topic | Kim, H., Shahbal, H., Parpia, S., Averbuch, T., Van Spall, H. G. C., Thabane, L., & Ma, J. (2023). Trials using composite outcomes neglect the presence of competing risks: a methodological survey of cardiovascular studies. *Journal of Clinical Epidemiology*, *160*, 1–13. https://doi.org/10.1016/j.jclinepi.2023.05.015 |
| 194 | NA, 2007 | Excluded: wrong study design dan publication type | Vascular Disease, Hypertension and Prevention. (2007). *Journal of the American College of Cardiology*, *49*(9), A315–A419. https://doi.org/10.1016/j.jacc.2007.01.043 |
| 195 | Serruys, 2021 | Excluded: wrong study design dan publication type | Serruys, P. W., Hara, H., Garg, S., Kawashima, H., Nørgaard, B. L., Dweck, M. R., Bax, J. J., Knuuti, J., Nieman, K., Leipsic, J. A., Mushtaq, S., Andreini, D., & Onuma, Y. (2021). Coronary Computed Tomographic Angiography for Complete Assessment of Coronary Artery Disease. *Journal of the American College of Cardiology*, *78*(7), 713–736. https://doi.org/10.1016/j.jacc.2021.06.019 |
| 196 | Formica, 2016 | Excluded: wrong study design dan publication type | Formica, F., Bamodu, O. A., Mariani, S., & Paolini, G. (2016). Post-valvular surgery multi-vessel coronary artery spasm — A literature review. *IJC Heart & Vasculature*, *10*, 32–38. https://doi.org/10.1016/j.ijcha.2015.10.010 |
| 197 | Kushner, 2013 | Excluded: wrong study design dan publication type | Kushner, F. G., & Bates, E. R. (2013). ST-Segment Elevation Myocardial Infarction. In *Cardiovascular Therapeutics: A Companion to Braunwald’s Heart Disease* (pp. 178–213). Elsevier. https://linkinghub.elsevier.com/retrieve/pii/B9781455701018000102 |
| 198 | Bavishi, 2016 | Excluded: wrong study design dan publication type | Bavishi, C., Panwar, S. R., Dangas, G. D., Barman, N., Hasan, C. M., Baber, U., Kini, A. S., & Sharma, S. K. (2016). Meta-Analysis of Radial Versus Femoral Access for Percutaneous Coronary Interventions in Non–ST-Segment Elevation Acute Coronary Syndrome. *The American Journal of Cardiology*, *117*(2), 172–178. https://doi.org/10.1016/j.amjcard.2015.10.039 |
| 199 | Kern, 2018 | Excluded: wrong study design dan publication type | Kern, M. J. (2018). The Basics of Percutaneous Coronary Interventions. In *The Interventional Cardiac Catheterization Handbook* (pp. 1–50). Elsevier. https://linkinghub.elsevier.com/retrieve/pii/B9780323476713000016 |
| 200 | Reeder, 2000 | Excluded: wrong study design dan publication type | Reeder, G. S., & Gersh, B. J. (2000). Modern Management of Acute Myocardial Infarction. *Current Problems in Cardiology*, *25*(10), 677–782. https://doi.org/10.1067/mcd.2000.109091 |
| 201 | Mohd Faizal, 2021 | Excluded: wrong study design dan publication type | Mohd Faizal, A. S., Thevarajah, T. M., Khor, S. M., & Chang, S.-W. (2021). A review of risk prediction models in cardiovascular disease: conventional approach vs. artificial intelligent approach. *Computer Methods and Programs in Biomedicine*, *207*, 106190. https://doi.org/10.1016/j.cmpb.2021.106190 |
| 202 | NA, 2021 | Excluded: wrong study design dan publication type | The International Symposium on Endovascular Therapy (ISET) 2021. (2021). *Journal of Vascular and Interventional Radiology*, *32*(5), e1–e32. https://doi.org/10.1016/j.jvir.2021.03.539 |
| 203 | Hu, 2021 | Excluded: wrong study design dan publication type | Hu, M. J., Li, X. S., Jin, C., & Yang, Y. J. (2021). Does multivessel revascularization fit all patients with STEMI and multivessel coronary artery disease? A systematic review and meta-analysis. In *IJC Heart and Vasculature* (Vol. 35). Elsevier Ireland Ltd. https://doi.org/10.1016/j.ijcha.2021.100813 |
| 204 | Wernly, 2004 | Excluded: wrong study design dan publication type | Wernly, J. A. (2004). Ischemia, reperfusion, and the role of surgery in the treatment of cardiogenic shock secondary to acute myocardial infarction: an interpretative review. *Journal of Surgical Research*, *117*(1), 6–21. https://doi.org/10.1016/j.jss.2003.12.024 |
| 205 | Hamaya, 2021 | Excluded: wrong study design dan publication type | Hamaya, R., Chang, Y. T., Chewcharat, A., Chiu, N., Yonetsu, T., Kakuta, T., & Papatheodorou, S. (2021). Comparison of invasive treatment strategies in patients with non–ST elevation acute coronary syndrome: A systematic review and meta-analysis. *JTCVS Open*, *8*, 323–335. https://doi.org/10.1016/j.xjon.2021.08.028 |
| 206 | Bavishi, 2017 | Excluded: irrelevant topic | Bavishi, C., Trivedi, V., Singh, M., Katz, E., Messerli, F. H., & Bangalore, S. (2017). Duration of Dual Antiplatelet Therapy in Patients with an Acute Coronary Syndrome Undergoing Percutaneous Coronary Intervention. *The American Journal of Medicine*, *130*(11), 1325.e1-1325.e12. https://doi.org/10.1016/j.amjmed.2017.05.029 |
| 207 | Suzuki, 2007 | Excluded: wrong outcome | Suzuki, N., Kozuma, K., Kyono, H., Ueno, Y., Nagaoka, K., Watari, Y., Endo, G., Terakura, M., Shiga, J., & Isshiki, T. (2007). Angiographic and clinical characteristics associated with the removable plaque components by means of thrombectomy catheters in patients with myocardial infarction. *Cardiovascular Revascularization Medicine*, *8*(4), 236–242. https://doi.org/10.1016/j.carrev.2007.03.006 |
| 208 | Verschuren, 2013 | Excluded: irrelevant topic | Verschuren, J. J. W., Boden, H., Wessels, J. A. M., Van Der Hoeven, B. L., Trompet, S., Heijmans, B. T., Putter, H., Guchelaar, H.-J., Schalij, M. J., & Jukema, J. W. (2013). Value of platelet pharmacogenetics in common clinical practice of patients with ST-segment elevation myocardial infarction. *International Journal of Cardiology*, *167*(6), 2882–2888. https://doi.org/10.1016/j.ijcard.2012.07.020 |
| 209 | Wholey, 2004 | Excluded: wrong study design dan publication type | Wholey, M. H., Wholey, M. H., & Eles, G. (2004). Endovascular Procedures for Carotid Artery Occlusive Disease. *Techniques in Vascular and Interventional Radiology*, *7*(4), 168–186. https://doi.org/10.1053/j.tvir.2005.03.010 |
| 210 | Pons, 2022 | Excluded: irrelevant topic | Pons, A., Whalley, G., Sneddon, K., Williams, M., & Coffey, S. (2022). Predictors of quality of life after revascularization for ischemic heart disease: A systematic review. *Health Sciences Review*, *2*, 100017. https://doi.org/10.1016/j.hsr.2022.100017 |
| 211 | García, 2001 | Excluded: foreign language | García Díaz, F., Pérez Márquez, M., Molina Gay, J., Sánchez Olmedo, J. I., Frías Ochoa, J., & Pérez Alé, M. (2001). El infarto de miocardio en el diabético: implicaciones clínicas, pronósticas y terapéuticas en la era trombolítico-intervencionista. *Medicina Intensiva*, *25*(8), 311–320. https://doi.org/10.1016/S0210-5691(01)79711-2 |
| 212 | Kern, 2013 | Excluded: wrong study design dan publication type | Kern, M. J. (2013). Basics of Percutaneous Coronary Interventions. In *Interventional Cardiac Catheterization Handbook* (pp. 1–37). Elsevier. |
| 213 | Hu, 2022 | Excluded: wrong population | Hu, M.-J., Yang, Y.-J., & Yang, J.-G. (2022). Immediate Versus Staged Multivessel PCI Strategies in Patients with ST-Segment Elevation Myocardial Infarction and Multivessel Disease: A Systematic Review and Meta-Analysis. *The American Journal of the Medical Sciences*, *363*(2), 161–173. https://doi.org/10.1016/j.amjms.2021.06.017 |
| 214 | Rahman, 2018 | Excluded: irrelevant topic | Rahman, H., Khan, S. U., Lone, A. N., & Kaluski, E. (2018). Revascularization strategies in cardiogenic shock complicating acute myocardial infarction: A systematic review and meta-analysis. *Cardiovascular Revascularization Medicine*, *19*(6), 647–654. https://doi.org/10.1016/j.carrev.2018.06.004 |
| 215 | Zhou, 2019 | Excluded: wrong study design dan publication type | Zhou, P., Zhu, P., Nie, Z., & Zheng, S. (2019). Is the era of bilateral internal thoracic artery grafting coming for diabetic patients? An updated meta-analysis. *The Journal of Thoracic and Cardiovascular Surgery*, *158*(6), 1559-1570.e2. |
| 216 | NA, 2013 | Excluded: wrong study design dan publication type | A Review of JACC Journal Articles on the Topic of Interventional Cardiology: 2011–2012. (2013). *Journal of the American College of Cardiology*, *62*(18), e245–e437. https://doi.org/10.1016/j.jacc.2013.09.004 |
| 217 | Hojs, 2012 | Excluded: irrelevant topic | Hojs, R., Bevc, S., & Ekart, R. (2012). Biomarkers in Hemodialysis Patients. In *Advances in Clinical Chemistry* (Vol. 57, pp. 29–56). Elsevier. https://linkinghub.elsevier.com/retrieve/pii/B9780123943842000024 |
| 218 | Mettler, 2012 | Excluded: wrong study design dan publication type | Mettler, F. A., & Guiberteau, M. J. (2012). Cardiovascular System. In *Essentials of Nuclear Medicine Imaging* (pp. 131–193). Elsevier. https://linkinghub.elsevier.com/retrieve/pii/B9781455701049000056 |
| 219 | Potpara, 2023 | Excluded: irrelevant topic | Potpara, T., Angiolillo, D. J., Bikdeli, B., Capodanno, D., Cole, O., Yataco, A. C., Dan, G.-A., Harrison, S., Iaccarino, J. M., Moores, L. K., Ntaios, G., & Lip, G. Y. H. (2023). Antithrombotic Therapy in Arterial Thrombosis and Thromboembolism in COVID-19. *CHEST*, *164*(6), 1531–1550. https://doi.org/10.1016/j.chest.2023.06.032 |
| 220 | Moretti, 2015 | Excluded: wrong study design dan publication type | Moretti, C., D’Ascenzo, F., Quadri, G., Omedè, P., Montefusco, A., Taha, S., Cerrato, E., Colaci, C., Chen, S.-L., Biondi-Zoccai, G., & Gaita, F. (2015). Management of multivessel coronary disease in STEMI patients: A systematic review and meta-analysis. *International Journal of Cardiology*, *179*, 552–557. https://doi.org/10.1016/j.ijcard.2014.10.035 |
| 221 | Nagaraja, 2017 | Excluded: irrelevant topic | Nagaraja, V., Mamas, M., Mahmoudi, M., Rogers, C., & Curzen, N. (2017). Change in angiogram-derived management strategy of patients with chest pain when some FFR data are available: How consistent is the effect? *Cardiovascular Revascularization Medicine*, *18*(5), 320–327. https://doi.org/10.1016/j.carrev.2017.01.014 |
| 222 | Nezami, 2021 | Excluded: wrong study design dan publication type | Nezami, F. R., Athanasiou, L. S., & Edelman, E. R. (2021). Endovascular drug-delivery and drug-elution systems. In *Biomechanics of Coronary Atherosclerotic Plaque* (pp. 595–631). Elsevier. https://linkinghub.elsevier.com/retrieve/pii/B9780128171950000287 |
| 223 | NA, 2001 | Excluded: irrelevant topic | Hypertension, vascular disease, and prevention. (2001). *Journal of the American College of Cardiology*, *37*(2), A220–A304. https://doi.org/10.1016/S0735-1097(01)80004-8 |
| 224 | Yalçınkaya, 2016 | Excluded: irrelevant topic | Yalçınkaya, A., Lafçı, G., Diken, A. İ., Aksoy, E., Çiçek, Ö. F., Lafçı, A., Korkmaz, K., & Çağlı, K. (2016). Early Mortality and Long-term Survival after Repair of Post-infarction Ventricular Septal Rupture: An Institutional Report of Experience. *Heart, Lung and Circulation*, *25*(4), 384–391. https://doi.org/10.1016/j.hlc.2015.08.016 |
| 225 | von Birgelen, 2018 | Excluded: wrong drug | von Birgelen, C., Zocca, P., Buiten, R. A., Jessurun, G. A. J., Schotborgh, C. E., Roguin, A., Danse, P. W., Benit, E., Aminian, A., van Houwelingen, K. G., Anthonio, R. L., Stoel, M. G., Somi, S., Hartmann, M., Linssen, G. C. M., Doggen, C. J. M., & Kok, M. M. (2018). Thin composite wire strut, durable polymer-coated (Resolute Onyx) versus ultrathin cobalt–chromium strut, bioresorbable polymer-coated (Orsiro) drug-eluting stents in allcomers with coronary artery disease (BIONYX): an international, single-blind, randomised non-inferiority trial. *The Lancet*, *392*(10154), 1235–1245. https://doi.org/10.1016/S0140-6736(18)32001-4 |
| 226 | Kovalic, 2018 | Excluded: irrelevant topic | Kovalic, A. J., & Satapathy, S. K. (2018). The Role of Nonalcoholic Fatty Liver Disease on Cardiovascular Manifestations and Outcomes. *Clinics in Liver Disease*, *22*(1), 141–174. https://doi.org/10.1016/j.cld.2017.08.011 |
| 227 | Doulamis, 2021 | Excluded: wrong population | [Doulamis, Ilias P., Aspasia Tzani, Andreas Tzoumas, Dimitrios C. Iliopoulos, Polydoros N. Kampaktsis, and Alexandros Briasoulis. 2021. “Percutaneous Coronary Intervention With Drug Eluting Stents Versus Coronary Artery Bypass Graft Surgery in Patients With Advanced Chronic Kidney Disease: A Systematic Review and Meta-Analysis.” Seminars in Thoracic and Cardiovascular Surgery 33 (4): 958–69.](http://paperpile.com/b/KSp2Lw/5Eqm) |
| 228 | Mettler, 2019 | Excluded: wrong study design dan publication type | Mettler, F. A., & Guiberteau, M. J. (2019). Cardiovascular System. In *Essentials of Nuclear Medicine and Molecular Imaging* (pp. 116–174). Elsevier. https://linkinghub.elsevier.com/retrieve/pii/B9780323483193000055 |
| 229 | Ullah, 2021 | Excluded: wrong study design dan publication type | Ullah, W., Zahid, S., Nadeem, N., Gowda, S., Munir, S., Saleem, S., Alraies, M. C., Alam, M., & Fischman, D. L. (2021). Meta-Analysis Comparing Culprit-Only Versus Complete Multivessel Percutaneous Coronary Intervention in Patients With ST-Elevation Myocardial Infarction. *The American Journal of Cardiology*, *139*, 34–39. https://doi.org/10.1016/j.amjcard.2020.10.009 |
| 230 | Chen, 2021 | Excluded: wrong population | Chen, X., Zhang, X., Yan, Y., & Wang, G. (2021). The Better Option of Revascularization in Complex Coronary Artery Disease Patients Complicate With Chronic Kidney Disease: A Review and Meta-Analysis. *Current Problems in Cardiology*, *46*(9), 100886. https://doi.org/10.1016/j.cpcardiol.2021.100886 |
| 231 | Trikalinos, 2009 | Excluded: wrong study design dan publication type | Trikalinos, T. A., Alsheikh-Ali, A. A., Tatsioni, A., Nallamothu, B. K., & Kent, D. M. (2009). Percutaneous coronary interventions for non-acute coronary artery disease: a quantitative 20-year synopsis and a network meta-analysis. *The Lancet*, *373*(9667), 911–918. https://doi.org/10.1016/S0140-6736(09)60319-6 |
| 232 | Bavry, 2008 | Excluded: wrong outcome | Bavry, A. A., & Bhatt, D. L. (2008). Appropriate use of drug-eluting stents: balancing the reduction in restenosis with the concern of late thrombosis. *The Lancet*, *371*(9630), 2134–2143. https://doi.org/10.1016/S0140-6736(08)60922-8 |
| 233 | NA, 2000 | Excluded: wrong study design dan publication type | 2000 Transcatheter cardiovascular therapeutics. (2000). *The American Journal of Cardiology*, *86*(8), 1i–38i. https://doi.org/10.1016/S0002-9149(00)80013-2 |
| 234 | NA, 2006 | Excluded: wrong study design dan publication type | Myocardial Ischemia and Infarction. (2006). *Journal of the American College of Cardiology*, *47*(4), A158–A236. https://doi.org/10.1016/j.jacc.2006.01.014 |
| 235 | Bates, 2007 | Excluded: wrong study design dan publication type | Bates, E. R., & Kushner, F. G. (2007). ST-Elevation Myocardial Infarction. In *Cardiovascular Therapeutics* (pp. 246–289). Elsevier. https://linkinghub.elsevier.com/retrieve/pii/B9781416033585500176 |
| 236 | NA, 2016 | Excluded: wrong study design dan publication type | PO03-01 to PO03-220. (2016). *Heart Rhythm*, *13*(5), S251–S339. https://doi.org/10.1016/j.hrthm.2016.03.029 |
| 237 | Shabetai, 1987 | Excluded: wrong study design dan publication type | Shabetai, R., & Sahn, D. J. (1987). Abstracts of papers to be presented at the 36th Annual Scientific Session of the American College of Cardiology, New Orleans, Louisiana, March 8–12, 1987. *Journal of the American College of Cardiology*, *9*(2), 1A-255A. https://doi.org/10.1016/S0735-1097(87)80368-6 |
| 238 | NA, 2000 | Excluded: wrong study design dan publication type | Subject index. (2000). *Journal of the American College of Cardiology*, *35*(2), A603–A703. https://doi.org/10.1016/S0735-1097(00)80011-X |
| 239 | Cousins, 2013 | Excluded: irrelevant topic | Cousins, C., Miller, D. L., Bernardi, G., Rehani, M. M., Schofield, P., Vañó, E., Einstein, A. J., Geiger, B., Heintz, P., Padovani, R., & Sim, K.-H. (2013). ICRP Publication 120: Radiological Protection in Cardiology. *Annals of the ICRP*, *42*(1), 1–125. https://doi.org/10.1016/j.icrp.2012.09.001 |
| 240 | NA, 2001 | Excluded: irrelevant topic | Cardiac arrhythmias. (2001). *Journal of the American College of Cardiology*, *37*(2), A87–A141. https://doi.org/10.1016/S0735-1097(01)80002-4 |
| 241 | Kaur, 2012 | Excluded: irrelevant topic | Kaur, P., Ghariwala, V., Yeo, K. S., Tan, H. Z., Tan, J. C. S., Armugam, A., Strong, P. N., & Jeyaseelan, K. (2012). Biochemistry of Envenomation. In *Advances in Clinical Chemistry* (Vol. 57, pp. 187–252). Elsevier. https://linkinghub.elsevier.com/retrieve/pii/B9780123943842000073 |
| 242 | Lerch, 2008 | Excluded: wrong study design dan publication type | Lerch, M. M., & Malecka-Panas, E. (2008). Joint Meeting of the European Pancreatic Club (EPC) and the International Association of Pancreatology (IAP). *Pancreatology*, *8*(3), 285–400. https://doi.org/10.1159/000134280 |
| 243 | Daly, 2017 | Excluded: wrong study design dan publication type | Daly, M., Melton, I., & Crozier, I. (2017). Implantable Cardiac Rhythm and Hemodynamic Monitors. In *Clinical Cardiac Pacing, Defibrillation and Resynchronization Therapy* (pp. 602–628). Elsevier. https://linkinghub.elsevier.com/retrieve/pii/B9780323378048000250 |
| 244 | Gossage, 1994 | Excluded: wrong study duration | Gossage, J. R. (1994). Acute Myocardial Infarction. *Chest*, *106*(6), 1851–1866. https://doi.org/10.1378/chest.106.6.1851 |
| 245 | Urban, 1989 | Excluded: irrelevant topic | Urban, P., Sigwart, U., Golf, S., Kaufmann, U., Sadeghi, H., & Kappenberger, L. (1989). Intravascular stenting for stenosis of aortocoronary venous bypass grafts. *Journal of the American College of Cardiology*, *13*(5), 1085–1091. https://doi.org/10.1016/0735-1097(89)90265-9 |
| 246 | NA, 1996 | Excluded: wrong study design dan publication type | Abstracts 1199–615A. (1996). *Journal of Urology*, *155*(5S). https://doi.org/10.1016/S0022-5347(96)80135-2 |
| 247 | NA, 2021 | Excluded: wrong study design dan publication type | Unmoderated Posters. (2014). *Urology*, *84*(4), S171–S387. https://doi.org/10.1016/S0090-4295(14)01020-6 |
| 248 | NA, 2012 | Excluded: wrong study design dan publication type | Electronic Posters. (2012). *Urology*, *80*(3), S189–S345. https://doi.org/10.1016/S0090-4295(12)00882-5 |
| 249 | NA, 2015 | Excluded: wrong study design dan publication type | MINI ORAL SESSIONS. (2015). *Journal of Thoracic Oncology*, *10*(9), S261–S406. https://doi.org/10.1016/S1556-0864(16)30011-9 |
| 250 | NA, 2008 | Excluded: irrelevant topic | Abstracts for the International Investigative Dermatology 2008. (2008). *Journal of Dermatological Science*, *50*(2), e1–e285. https://doi.org/10.1016/j.jdermsci.2008.03.001 |
| 251 | NA, 2017 | Excluded: wrong study design dan publication type | Abstracts of the 12th Annual Scientific Meeting of the Society of Cardiovascular Computed Tomography. (2017). *Journal of Cardiovascular Computed Tomography*, *11*(4), S1–S82. https://doi.org/10.1016/j.jcct.2017.05.005 |
| 252 | NA, 1984 | Excluded: wrong study design dan publication type | Abstracts of Papers to be Presented at the 33rd Annual Scientific Session of the American College of Cardiology Dallas, Texas, March 25–29, 1984. (1984). *Journal of the American College of Cardiology*, *3*(2), 469–624. https://doi.org/10.1016/S0735-1097(84)80001-7 |
| 253 | Turi, 2008 | Excluded: wrong study design dan publication type | Turi, Z. G. (2008). Valvular Heart Disease in Critical Care. In *Critical Care Medicine* (pp. 677–707). Elsevier. https://linkinghub.elsevier.com/retrieve/pii/B9780323048415500352 |
| 254 | NA, 2015 | Excluded: wrong study design dan publication type | Abstracts from the 19th Annual North American Neuromodulation Society Meeting (NANS) Las Vegas, NV, USA December 10-13, 2015. (2016). *Neuromodulation: Technology at the Neural Interface*, *19*(3), e1–e158. https://doi.org/10.1111/ner.12428 |
| 255 | NA, 2005 | Excluded: wrong study design dan publication type | Abstract pages for DDW 2005. (2005). *Gastroenterology*, *128*(4), A1–A821. https://doi.org/10.1053/j.gastro.2005.04.003 |
| 256 | NA, 2004 | Excluded: wrong study design dan publication type | [AGA Abstracts S1448–M1084. (2004). Gastroenterology, 126(4), A220–A293. https://doi.org/10.1016/S0016-5085(04)80010-6](https://doi.org/10.1016/S0016-5085(04)80010-6) |
| 257 | Serruys, 2021 | Excluded: wrong study design dan publication type | Serruys, P. W., Hara, H., Garg, S., Kawashima, H., Nørgaard, B. L., Dweck, M. R., Bax, J. J., Knuuti, J., Nieman, K., Leipsic, J. A., Mushtaq, S., Andreini, D., & Onuma, Y. (2021). Coronary Computed Tomographic Angiography for Complete Assessment of Coronary Artery Disease. *Journal of the American College of Cardiology*, *78*(7), 713–736. https://doi.org/10.1016/j.jacc.2021.06.019 |
| 258 | NA, 2010 | Excluded: irrelevant topic | 42nd European Pancreatic Club (EPC) Meeting. (2010). *Pancreatology*, *10*(2–3), 259–400. https://doi.org/10.1159/000314328 |
| 259 | NA, 2016 | Excluded: wrong study design dan publication type | Poster presentations. (2016). *European Geriatric Medicine*, *7*, S29–S259. https://doi.org/10.1016/S1878-7649(16)30149-8 |
| 260 | NA, 1989 | Excluded: wrong study design dan publication type | Abstracts of papers to be presented at the 38th Annual Scientific Session of the American College of Cardiology, Anaheim, California, March 19–23, 1989. (1989). *Journal of the American College of Cardiology*, *13*(2), 1A-254A. https://doi.org/10.1016/S0735-1097(89)80001-4 |
| 261 | NA, 1996 | Excluded: wrong study design dan publication type | Abstracts 800–1198. (1996). *Journal of Urology*, *155*(5S). https://doi.org/10.1016/S0022-5347(96)80134-0 |
| 262 | NA, 2014 | Excluded: wrong study design dan publication type | International Symposium on Endovascular Therapy (ISET) 2014. (2014). *Journal of Vascular and Interventional Radiology*, *25*(1), e1–e24. https://doi.org/10.1016/j.jvir.2013.11.028 |
| 263 | Fordyce, 2018 | Excluded: irrelevant topic | Fordyce, C. B., & Douglas, P. S. (2018). Putting It All Together. In *Chronic Coronary Artery Disease* (pp. 204–225). Elsevier. https://linkinghub.elsevier.com/retrieve/pii/B9780323428804000157 |
| 264 | NA, 2014 | Excluded: wrong study design dan publication type | Moderated Poster Sessions. (2014). *Urology*, *84*(4), S1–S146. https://doi.org/10.1016/S0090-4295(14)01017- |
| 265 | Turi, 2022 | Excluded: wrong study design dan publication type | Turi, Z. G. (2022). The 40th Anniversary of Percutaneous Balloon Valvuloplasty for Mitral Stenosis: Current Status. *Structural Heart*, *6*(5), 100087. https://doi.org/10.1016/j.shj.2022.100087 |
| 266 | NA, 2019 | Excluded: wrong study design dan publication type | [Index. (2019). In Comprehensive Biotechnology (pp. 565–655). Elsevier. https://linkinghub.elsevier.com/retrieve/pii/B9780444640468180012](https://linkinghub.elsevier.com/retrieve/pii/B9780444640468180012) |
| 267 | NA, 2005 | Excluded: wrong study design dan publication type | Abstracts Presented for the Thirty-Sixth Society of Gynecologic Oncologist. (2005). *Gynecologic Oncology*, *96*(3), 909–1020. https://doi.org/10.1016/j.ygyno.2005.01.006 |
| 268 | NA, 2004 | Excluded: wrong study design dan publication type | SSAT abstracts 192–M2127. (2004). *Gastroenterology*, *126*(4), A768–A815. https://doi.org/10.1016/S0016-5085(04)80018-0 |
| 269 | NA, 2005 | Excluded: foreign language | Deutsche Gesellschaft fur Klinische Neurophysiologie und Funktionelle Bildgebunge. V. (DGKN). (2005). *Clinical Neurophysiology*, *116*(9), e25–e132. https://doi.org/10.1016/j.clinph.2005.05.002 |
| 270 | NA, 2018 | Excluded: wrong study design dan publication type | Abstracts of the 13th Annual Scientific Meeting of the Society of Cardiovascular Computed Tomography. (2018). *Journal of Cardiovascular Computed Tomography*, *12*(3), S1–S74. https://doi.org/10.1016/j.jcct.2018.05.019 |
| 271 | NA, 2016 | Excluded: wrong study design dan publication type | [PO02-01 to PO02-219. (2016). Heart Rhythm, 13(5), S166–S250. https://doi.org/10.1016/j.hrthm.2016.03.028](https://doi.org/10.1016/j.hrthm.2016.03.028) |
| 272 | NA, 2015 | Excluded: wrong study design dan publication type | Abstracts of the 10th Annual Scientific Meeting of the Society of Cardiovascular Computed Tomography. (2015). *Journal of Cardiovascular Computed Tomography*, *9*(4), S1–S96. https://doi.org/10.1016/j.jcct.2015.05.006 |
| 273 | Ruskin, 1993 | Excluded: wrong study design dan publication type | Ruskin, J. N., & Fuster, V. (1993). Abstracts of original contributions: 42nd Annual Scientific Session. *Journal of the American College of Cardiology*, *21*(2), 17A-488A. https://doi.org/10.1016/S0735-1097(10)80309-2 |
| 274 | NA, 2012 | Excluded: wrong study design dan publication type | Moderated Poster Sessions. (2012). *Urology*, *80*(3), S36–S177. https://doi.org/10.1016/S0090-4295(12)00880-1 |
| 275 | NA, 2001 | Excluded: wrong study design dan publication type | Abstracts of the 49th annual scientific meeting of the cardiac society of Australia and New Zealand. (2001). *Heart, Lung and Circulation*, *10*(3), A55–A152. https://doi.org/10.1046/j.1444-2892.2001.00002.x |
| 276 | NA, 2000 | Excluded: wrong study design dan publication type | Abstract. (2000). *Pathology - Research and Practice*, *196*(6), 345–458. https://doi.org/10.1016/S0344-0338(00)80103-X |
| 277 | NA, 2018 | Excluded: wrong study design dan publication type | Full Issue PDF. (2018). *JACC: Cardiovascular Imaging*, *11*(10), e917–e1097. https://doi.org/10.1016/S1936-878X(18)30805-2 |
| 278 | NA, 2016 | Excluded: wrong study design dan publication type | Abstracts. (2006). *HPB*, *8*, 3–255. https://doi.org/10.1080/16515320600855338 |
| 279 | NA, 2015 | Excluded: irrelevant topic | Cardiac arrhythmias. (2005). *Journal of the American College of Cardiology*, *45*(3), A91–A128. https://doi.org/10.1016/j.jacc.2004.12.036 |
| 280 | NA, 1995 | Excluded: wrong publication type | [“Monday 3 July 1995 Abstracts Mo1-Mo188.” Journal of Molecular and Cellular Cardiology 27, no. 6 (June 1995): A77–298. https://doi.org/10.1016/S0022-2828(05)82398-3.](https://doi.org/10.1016/S0022-2828(05)82398-3) |
| 281 | Matthew, 2020 | Excluded: irrelevant topic | [“D-PO05-015 to D-PO05-244.” Heart Rhythm 17, no. 5 (May 2020): S477–570. https://doi.org/10.1016/j.hrthm.2020.04.010.](https://doi.org/10.1016/j.hrthm.2020.04.010) |
| 282 | Giraldez, 2009 | Excluded: irrelevant topic | [“Posters.” Annals of Oncology 20 (June 2009): vii29–114. https://doi.org/10.1093/annonc/mdp284.](https://doi.org/10.1093/annonc/mdp284) |
| 283 | Guido, 1992 | Excluded: irrelevant topic | [“Author Index.” Journal of Vascular and Interventional Radiology 3, no. 1 (February 1992): 150–94. https://doi.org/10.1016/S1051-0443(92)72208-8.](https://doi.org/10.1016/S1051-0443(92)72208-8) |
| 284 | NA, 2007 | Excluded: irrelevant topic | [“DGP-Abstracts.” Pathology - Research and Practice 203, no. 5 (May 2007): 251–419. https://doi.org/10.1016/j.prp.2007.03.004.](https://doi.org/10.1016/j.prp.2007.03.004) |
| 285 | Elscot, 2023 | Excluded: wrong outcome | Elscot JJ, Kakar H, Scarparo P, Dekker WK den, Bennett J, Schotborgh CE, et al. Timing of complete multivessel revascularization in patients presenting with non-ST-elevation acute coronary syndrome [Internet]. medRxiv. 2023. Available from: http://medrxiv.org/lookup/doi/10.1101/2023.05.29.23290502 |
| 286 | Yamamoto, 2023 | Excluded: wrong outcome | [Yamamoto K, Shiomi H, Morimoto T, Miyazawa A, Watanabe H, Natsuaki M, et al. Target lesion revascularization after intravascular ultrasound-guided percutaneous coronary intervention. Circ Cardiovasc Interv. 2023 May;16(5):e012922.](http://paperpile.com/b/79k7lV/8beS) |
| 287 | Rossi, 2023 | Excluded: wrong publication type | [Rossi S, Perfetti M, Mantini C, Cicchitti V, Zimarino M. P303 ecmo–protected high risk pci in unusual clinical and anatomical scenario. Eur Heart J Suppl. 2023 May 18;25(Supplement_D):D159–D159.](http://paperpile.com/b/79k7lV/SzGU) |
| 288 | Kadir, 2023 | Excluded: wrong publication type | [Kadir AA, Ong YY, How NK, Lu HT. TCTAP C-093 complex calcified left main and LAD percutaneous coronary intervention after CABG. J Am Coll Cardiol. 2023 Apr;81(16):S241–3.](http://paperpile.com/b/79k7lV/n7Kr) |
| 289 | Saito, 2023 | Excluded: wrong study design | [Saito Y, Kobayashi Y. Complete revascularization in acute myocardial infarction: a clinical review. Cardiovasc Interv Ther. 2023 Apr;38(2):177–86.](http://paperpile.com/b/79k7lV/4qwx) |
| 290 | Korjian, 2023 | Excluded: irrelevant topic | [Korjian S, Bahit MC, Daaboul Y, Chi G, Karabay AK, Gabriel S, et al. The economic and humanistic burden of multivessel disease in acute myocardial infarction: A systematic review. J Am Coll Cardiol. 2023 Mar;81(8):1317.](http://paperpile.com/b/79k7lV/aFoS) |
| 291 | Stoler, 2023 | Excluded: irrelevant topic | [Stoler R, Wood F, Hawa Z, Batchelor W, Stein B, Yeh R, et al. CRT-100.52 primary clinical outcomes of the bioabsorbable polymer-coated, everolimus-eluting synergy megatron Stent in the treatment of large coronary vessels. JACC Cardiovasc Interv. 2023 Feb;16(4):S24–5.](http://paperpile.com/b/79k7lV/IH8b) |
| 292 | Shin, 2023 | Excluded: irrelevant topic | [Shin ES, Jun EJ, Han JK, Kong MG, Kang J, Zheng C, et al. Sex-related impact on clinical outcomes of patients treated with drug-eluting stents according to clinical presentation: Patient-level pooled analysis from the GRAND-DES registry. Cardiol J. 2023;30(1):105–16.](http://paperpile.com/b/79k7lV/pFSW) |
| 293 | Shin, 2023 | Excluded: wrong drug | [Shin ES, Jun EJ, Kim S, Kim B, Kim TH, Sohn CB, et al. Clinical impact of drug-coated balloon-based percutaneous coronary intervention in patients with multivessel coronary artery disease. JACC Cardiovasc Interv. 2023 Feb 13;16(3):292–9.](http://paperpile.com/b/79k7lV/O6kf) |
| 294 | Lee, 2023 | Excluded: irrelevant topic | [Lee JM, Kim HK, Park KH, Choo EH, Kim CJ, Lee SH, et al. Fractional flow reserve versus angiography-guided strategy in acute myocardial infarction with multivessel disease: a randomized trial. Eur Heart J. 2023 Feb 7;44(6):473–84.](http://paperpile.com/b/79k7lV/kJGQ) |
| 295 | Garot, 2023 | Excluded: wrong outcome | [Garot P, Brunel P, Dibie A, Morelle JF, Abdellaoui M, Levy R, et al. Comparison of outcomes in patients with or without ARC-HBR criteria undergoing PCI with polymer-free biolimus coated stents: The BioFreedom France study. Catheter Cardiovasc Interv. 2023 Jan;101(1):60–71.](http://paperpile.com/b/79k7lV/5EYh) |
| 296 | Vergara-Uzcategui, 2023 | Excluded: wrong drug | [Vergara-Uzcategui CE, Moreno VH, Hennessey B, Sánchez-Del-Hoyo R, Donis JH, Gonzalez-Rojas J, et al. Duration and clinical outcomes of dual antiplatelet therapy following percutaneous coronary intervention for acute coronary syndrome: A multicentre “real-world practice” registry-based study. Front Cardiovasc Med. 2023 Apr 6;10:1158466.](http://paperpile.com/b/79k7lV/yaJY) |
| 297 | Liu, 2023 | Excluded: irrelevant topic | [Liu JD, Gong R, Xu JS, Zhang SY, Wu YQ. Clinical characteristics and outcomes of Chinese patients with premature acute coronary syndrome. Int Heart J. 2023 Mar 31;64(2):128–36.](http://paperpile.com/b/79k7lV/xOMa) |
| 298 | Zhang, 2023 | Excluded: wrong population | [Zhang J, Yao M, Jia X, Feng H, Fu J, Tang W, et al. The efficacy and safety of quantitative flow ratio-guided complete revascularization in patients with ST-segment elevation myocardial infarction and multivessel disease: A pilot randomized controlled trial. Cardiol J. 2023;30(2):178–87.](http://paperpile.com/b/79k7lV/kPzp) |
| 299 | Nishonov, 2023 | Excluded: foreign language | [Nishonov, A. B., R. S. Tarasov, S. V. Ivanov, and L. S. Barbarash. “Outcomes of Coronary Artery Bypass Grafting and Percutaneous Coronary Intervention in High-Risk Non-ST-Segment Elevation Acute Coronary Syndromes.” Complex Issues of Cardiovascular Diseases 12, no. 1 (March 31, 2023): 151–59. https://doi.org/10.17802/2306-1278-2023-12-1-151-159.](https://doi.org/10.17802/2306-1278-2023-12-1-151-159) |
| 300 | Plassmeier, 2023 | Excluded: irrelevant topic | Plassmeier F, Tauber J, Naito S, Reiter B, Sill B, Reichenspurner H, et al. Bilateral skeletonized internal mammary artery in insulin-dependent diabetic patients. In: The Thoracic and Cardiovascular Surgeon [Internet]. Georg Thieme Verlag KG; 2023. Available from: http://www.thieme-connect.de/DOI/DOI?10.1055/s-0043-1761711 |
| 301 | Chen, 2022 | Excluded: wrong outcome | [Chen X, Wu H, Li L, Zhao X, Zhang C, Wang WE. The prognostic utility of GRACE risk score in predictive adverse cardiovascular outcomes in patients with NSTEMI and multivessel disease. BMC Cardiovasc Disord. 2022 Dec 26;22(1):568.](http://paperpile.com/b/79k7lV/5h05) |
| 302 | Bruno, 2022 | Excluded: wrong outcome | Bruno F, Marengo G, Filippo OD, Wanha W, Leonardi S, Rubin SR, et al. 471 impact of complete revascularization on development of heart failure in patients with acute coronary syndrome and multivessel disease. Eur Heart J Suppl [Internet]. 2022 Dec 15;24(Supplement_K). Available from: https://academic.oup.com/eurheartjsupp/article/doi/10.1093/eurheartjsupp/suac121.300/6912049 |
| 303 | Ahn, 2022 | Excluded: wrong outcome | [Ahn JM, Kang DY, Yun SC, Ho Hur S, Park HJ, Tresukosol D, et al. Everolimus-eluting stents or bypass surgery for multivessel coronary artery disease: Extended follow-up outcomes of multicenter randomized controlled BEST trial. Circulation. 2022 Nov 22;146(21):1581–90.](http://paperpile.com/b/79k7lV/CUIh) |
| 304 | Gadre, 2022 | Excluded: wrong outcome | [Gadre AS, Sharma YP, Shanmugarajan A. An observational study to assess the feasibility, risks and benefits of robotic PCI(percutaneous coronary intervention) and the clinical profile and outcomes of patients undergoing robotic-PCI in a tertiary care centre in North India’. Indian Heart J. 2022 Nov;74:S63–4.](http://paperpile.com/b/79k7lV/w9GE) |
| 305 | Gornik, 2022 | Excluded: wrong drug | Gornik HL, Wood M, Naderi S, Leon K, Gibson CM, Chi GC, et al. Abstract 14370: Significant heterogeneity in antiplatelet regimen for spontaneous coronary artery dissection: A report of the ISCAD Registry. Circulation [Internet]. 2022 Nov 8;146(Suppl_1). Available from: https://www.ahajournals.org/doi/10.1161/circ.146.suppl_1.14370 |
| 306 | Sanetra, 2022 | Excluded: wrong study design | [Sanetra K, Buszman PP, Jankowska-Sanetra J, Cisowski M, Fil W, Gorycki B, et al. One-stage hybrid coronary revascularization for the treatment of multivessel coronary artery disease- Periprocedural and long-term results from the “HYBRID-COR” feasibility study. Front Cardiovasc Med. 2022 Oct 19;9:1016255.](http://paperpile.com/b/79k7lV/292f) |
| 307 | Chyrchel, 2022 | Excluded: wrong outcome | [Chyrchel M, Gallina T, Januszek R, Szafrański O, Gębska M, Surdacki A. The reduction of left ventricle ejection fraction after multi-vessel PCI during acute myocardial infarction as a predictor of major adverse cardiac events in long-term follow-up. Int J Environ Res Public Health. 2022 Oct 13;19(20):13160.](http://paperpile.com/b/79k7lV/eVun) |
| 308 | Pavasini, 2022 | Excluded: wrong study design | [Pavasini R, Sanguettoli F, Zanarelli L, Deserio MA, Bianchi N, Fabbri G, et al. Unsolved questions in the revascularization of older myocardial infarction patients with multivessel disease. Rev Cardiovasc Med. 2022 Oct;23(10):344.](http://paperpile.com/b/79k7lV/z7od) |
| 309 | Elscot, 2022 | Excluded: wrong publication type | [Elscot J, Scarparo P, Kakar H, Forero MT, Cummins P, Dekker W den, et al. TCT-10 revascularization strategies in patients presenting with non–ST-segment elevation acute coronary syndromes and multivessel coronary disease. J Am Coll Cardiol. 2022 Sep;80(12):B4–5.](http://paperpile.com/b/79k7lV/vzVD) |
| 310 | Brust, 2022 | Excluded: wrong publication type | [Brust K, Śmiech K, Bujak K, Roleder T, Gąsior M. TCT-106 the treatment of acute coronary syndromes related to multivessel coronary artery disease: Data from the PL-ACS registry. J Am Coll Cardiol. 2022 Sep;80(12):B43–4.](http://paperpile.com/b/79k7lV/T4X1) |
| 311 | Stoler, 2022 | Excluded: wrong outcome | [Stoler R, Wood F, Hawa Z, Rudick S, Divanji P, Yeh R, et al. TCT-159 percutaneous coronary intervention of large coronary vessels with the bioabsorbable polymer-coated, everolimus-eluting SYNERGY 4.5/5.0 mm Stent: Primary outcomes of the EVOLVE large vessel study. J Am Coll Cardiol. 2022 Sep;80(12):B64–5.](http://paperpile.com/b/79k7lV/jDl2) |
| 312 | Marengo, 2022 | Excluded: wrong outcome | Marengo G, D’Ascenzo F, De Filippo O, Bruno F. Incidence of heart failure after acute coronary syndrome in multivessel patients a subanalysis of the coralys registry. Eur Heart J [Internet]. 2022 Oct 3;43(Supplement_2). Available from: https://academic.oup.com/eurheartj/article/doi/10.1093/eurheartj/ehac544.2009/6745559 |
| 313 | Ekstroem, 2022 | Excluded: wrong outcome | Ekstroem K, Loenborg J, Nepper-Cristensen L, Holmvang L, Joshi FR, Iversen AZ, et al. Misclassification rate of the angiographically identified culprit lesion in NSTEMI. Eur Heart J [Internet]. 2022 Oct 3;43(Supplement_2). Available from: https://academic.oup.com/eurheartj/article/doi/10.1093/eurheartj/ehac544.1202/6745048 |
| 314 | Ahmed, 2022 | Excluded: wrong outcome | Ahmed TAN, Othman AAA, Demitry SR, El-Maghraby KM. Impact of residual coronary lesions on outcomes of myocardial infarction patients with multi-vessel disease. Eur Heart J [Internet]. 2022 Oct 3;43(Supplement_2). Available from: https://academic.oup.com/eurheartj/article/doi/10.1093/eurheartj/ehac544.1404/6745163 |
| 315 | Brust, 2022 | Excluded: wrong outcome | Brust K, Smiech K, Bujak K, Roleder T, Gasior M. The characteristic of acute coronary syndromes of patients with multivessel coronary artery disease in centers with and without cardiac surgery on-site – data from PL-ACS registry. Eur Heart J [Internet]. 2022 Oct 3;43(Supplement_2). Available from: https://academic.oup.com/eurheartj/article/doi/10.1093/eurheartj/ehac544.1407/6745160 |
| 316 | Geisler, 2022 | Excluded: wrong study design | [Geisler T, Branch K, Nikol S. Clinical experience with dual pathway inhibition therapy: case series and mini review. Eur Heart J Case Rep. 2022 Jul;6(7):ytac201.](http://paperpile.com/b/79k7lV/4UUW) |
| 317 | Telayna, 2022 | Excluded: irrelevant topic | [Telayna JM Jr, Costantini R, Krause S, Telayna JM. Does diabetes matter in chronic total coronary occlusion? Cardiovasc Revasc Med. 2022 Jul;40:41.](http://paperpile.com/b/79k7lV/5S5S) |
| 318 | Santos, 2022 | Excluded: irrelevant topic | [Abstracts of the heart failure 2022 and the world congress on acute heart failure, 21 - 24 may 2022, Madrid, Spain. Eur J Heart Fail. 2022 Jul;24 Suppl 2(S2):3–282.](http://paperpile.com/b/79k7lV/zTgs) |
| 319 | Nozari, 2022 | Excluded: wrong population | [Nozari Y, Mojtaba Ghorashi S, Alidoust M, Hamideh Mortazavi S, Jalali A, Omidi N, et al. In-hospital and 1-year outcomes of repeated percutaneous coronary intervention for in-stent restenosis with acute coronary syndrome presentation. Crit Pathw Cardiol. 2022 Jun 1;21(2):87–92.](http://paperpile.com/b/79k7lV/T14P) |
| 320 | Fouladvand, 2022 | Excluded: wrong population | [Fouladvand F, Iardino E, Cortese B, Di Palma G. TCTAP C-035 complex evaluation in multivessel coronary artery disease with common trunk critical involvement and complete revascularization during hospitalization. J Am Coll Cardiol. 2022 Apr;79(15):S128.](http://paperpile.com/b/79k7lV/b84Z) |
| 321 | Watanabe, 2022 | Excluded: wrong outcome | [Watanabe Y, Fujita T, Doi H, Tobaru T, Takanashi S, Kinoshita Y, et al. Prospective multicenter registry of hybrid coronary artery revascularization combined with non-saphenous vein graft surgical bypass and percutaneous coronary intervention using everolimus eluting metallic stents (PRIDE-METAL study). Cardiovasc Interv Ther. 2022 Apr;37(2):304–11.](http://paperpile.com/b/79k7lV/PIaq) |
| 322 | Telayna, 2022 | Excluded: wrong outcome | [Telayna JM Jr, Costantini R, Krause S, Telayna JM. CRT-100.65 does diabetes matter in chronic total coronary occlusion? JACC Cardiovasc Interv. 2022 Feb;15(4):S17.](http://paperpile.com/b/79k7lV/PRiq) |
| 323 | De Filippo, 2022 | Excluded: wrong outcome | [De Filippo O, Gallone G, D’Ascenzo F, Leone AM, Mancone M, Quadri G, et al. Predictors of fractional flow reserve/instantaneous wave-free ratio discordance: impact of tailored diagnostic cut-offs on clinical outcomes of deferred lesions. J Cardiovasc Med . 2022 Feb 1;23(2):106–15.](http://paperpile.com/b/79k7lV/kip8) |
| 324 | Siddiqui, 2022 | Excluded: wrong outcome | [Siddiqui AJ, Omerovic E, Holzmann MJ, Böhm F. Association of coronary angiographic lesions and mortality in patients over 80 years with NSTEMI. Open Heart. 2022 Jan;9(1):e001811.](http://paperpile.com/b/79k7lV/Oynz) |
| 325 | Lawton, 2022 | Excluded: wrong study design | [Writing Committee Members, Lawton JS, Tamis-Holland JE, Bangalore S, Bates ER, Beckie TM, et al. 2021 ACC/AHA/SCAI guideline for coronary artery revascularization: A report of the American college of cardiology/American heart association joint committee on clinical practice guidelines. J Am Coll Cardiol. 2022 Jan 18;79(2):e21–129.](http://paperpile.com/b/79k7lV/RfB6) |
| 326 | Lawton, 2022 | Excluded: wrong study design | [Lawton JS, Tamis-Holland JE, Bangalore S, Bates ER, Beckie TM, Bischoff JM, et al. 2021 ACC/AHA/SCAI guideline for coronary artery revascularization: A report of the American college of cardiology/American heart association joint committee on clinical practice guidelines. Circulation. 2022 Jan 18;145(3):e18–114.](http://paperpile.com/b/79k7lV/Gdyw) |
| 327 | Lawton, 2022 | Excluded: wrong study design | [Lawton, Jennifer S., Jacqueline E. Tamis-Holland, Sripal Bangalore, Eric R. Bates, Theresa M. Beckie, James M. Bischoff, John A. Bittl, et al. “2021 ACC/AHA/SCAI Guideline for Coronary Artery Revascularization: Executive Summary: A Report of the American College of Cardiology/American Heart Association Joint Committee on Clinical Practice Guidelines.” Circulation 145, no. 3 (January 18, 2022). https://doi.org/10.1161/CIR.0000000000001039.](https://doi.org/10.1161/CIR.0000000000001039) |
| 328 | Bahloul, 2022 | Excluded: wrong outcome | [Bahloul A, Ghorbel CH, Hammami R, Charfeddine S, Abid L, Kammoun S. Factors associated with major adverse cardiac events in very elderly patients with acute coronary syndrome. Arch Cardiovasc Dis Suppl. 2022 Jan;14(1):23–4.](http://paperpile.com/b/79k7lV/4iEH) |
| 329 | Anass, 2022 | Excluded: wrong publication type | [“Twenty-Third PanAfrican Course on Interventional Cardiology SMC-PAFCIC 2022.” Accessed September 24, 2024. https://cvja.co.za/onlinejournal/vol33/PAFCIC-Abstracts-2022/2/.](https://cvja.co.za/onlinejournal/vol33/PAFCIC-Abstracts-2022/2/) |
| 330 | Lawton, 2022 | Excluded: wrong study design | [Lawton, Jennifer S., Jacqueline E. Tamis-Holland, Sripal Bangalore, Eric R. Bates, Theresa M. Beckie, James M. Bischoff, John A. Bittl, et al. “2021 ACC/AHA/SCAI Guideline for Coronary Artery Revascularization: A Report of the American College of Cardiology/American Heart Association Joint Committee on Clinical Practice Guidelines.” Circulation 145, no. 3 (January 18, 2022). https://doi.org/10.1161/CIR.0000000000001038.](https://doi.org/10.1161/CIR.0000000000001038) |
| 331 | Borse, 2021 | Excluded: wrong outcome | [Borse AG, Roy S, Mitra A, Mitra KK. STEMI equivalent ECG changes in NSTEMI. Indian Heart J. 2021 Dec;73:S62.](http://paperpile.com/b/79k7lV/J5cO) |
| 332 | Goel, 2021 | Excluded: irrelevant topic | [Goel PK, Khanna R, Sahu AK. Procedural and short-term clinical outcomes post-PCI using newer generation ultrathin sirolimus-eluting stent with biodegradable polymer coating in real-world population. Indian Heart J. 2021 Dec;73:S57–8.](http://paperpile.com/b/79k7lV/fVp5) |
| 333 | Coughlan, 2021 | Excluded: wrong outcome | [Coughlan JJ, Aytekin A, Ndrepepa G, Schüpke S, Bernlochner I, Mayer K, et al. Twelve-month clinical outcomes in patients with acute coronary syndrome undergoing complex percutaneous coronary intervention: insights from the ISAR-REACT 5 trial. European Heart Journal Acute Cardiovascular Care [Internet]. 2021 Dec 18 [cited 2024 Sep 18];10(10):1117–24. Available from: https://academic.oup.com/ehjacc/article/10/10/1117/6361010](https://academic.oup.com/ehjacc/article/10/10/1117/6361010) |
| 334 | Mubarak, 2021 | Excluded: wrong publication type | [“Selected Abstracts from the PICS Society Symposium 2021 Aria Convention Center Las Vegas, NV September 1–4, 2021.” Pediatric Cardiology 42, no. 8 (December 2021): 1890–1946. https://doi.org/10.1007/s00246-021-02742-7.](https://doi.org/10.1007/s00246-021-02742-7) |
| 335 | Pierri, 2021 | Excluded: irrelevant topic | [Pierri A, De Luca A, Restivo L, Bologna A, Poletti A, Belgrano MG, et al. 324 Unilateral pulmonary artery agenesis with controlateral embolism: a double vascular trouble. European Heart Journal Supplements [Internet]. 2021 Dec 8 [cited 2024 Sep 18];23(Supplement_G):suab133.013. Available from: https://academic.oup.com/eurheartjsupp/article/doi/10.1093/eurheartj/suab133.013/6456894](https://academic.oup.com/eurheartjsupp/article/doi/10.1093/eurheartj/suab133.013/6456894) |
| 336 | Ielapi, 2021 | Excluded: wrong outcome | Ielapi, J., Rosa, S. de, Deietti, G., Critelli, C., Panuccio, G., Cacia, M. A., Luca, E. de, Strangio, A., Sorrentino, S., Polimeni, A., Sabatino, J., Pilò, A., Spaccarotella, C., Mongiardo, A., & Indolfi, C. (2021). 774 Young adults with acute coronary syndrome: still a long road ahead. *European Heart Journal Supplements*, *23*(Supplement_G), suab134.024. https://doi.org/10.1093/eurheartj/suab134.024 |
| 337 | Buccheri, 2021 | Excluded: irrelevant topic | [Buccheri, Dario, Renzo Lombardo, Daniele Vinci, and Arian Frasheri. “A ‘Parachute Effect’ Mockingly Sealing a Spontaneous Coronary Artery Dissection: What We Have Learnt from Intravascular Imaging.” Future Cardiology 17 (June 25, 2020). https://doi.org/10.2217/fca-2019-0068.](https://doi.org/10.2217/fca-2019-0068) |
| 338 | Kim, 2021 | Excluded: wrong population | [Kim BJ, Park JI, Nam JH, Lee JH, Lee CH, Son JW, et al. Clinical impact of intravascular ultrasound guidance in patients of ST-segment elevation myocardial infarction undergoing percutaneous coronary intervention with drug eluting stent. European Heart Journal [Internet]. 2021 Oct 12 [cited 2024 Sep 18];42(Supplement_1):ehab724.2084. Available from: https://academic.oup.com/eurheartj/article/doi/10.1093/eurheartj/ehab724.2084/6392041](https://academic.oup.com/eurheartj/article/doi/10.1093/eurheartj/ehab724.2084/6392041) |
| 339 | Zhao, 2021 | Excluded: wrong population | [Zhao X, Liu LC, Zhou ZP, Sheng SZX, Li LJN, Zhou ZJY, et al. Thrombosis and major bleeding risk after primary percutaneous coronary intervention among patients with multi-vessels coronary artery disease. European Heart Journal [Internet]. 2021 Oct 12 [cited 2024 Sep 19];42(Supplement_1):ehab724.1251. Available from: https://academic.oup.com/eurheartj/article/doi/10.1093/eurheartj/ehab724.1251/6392066](https://academic.oup.com/eurheartj/article/doi/10.1093/eurheartj/ehab724.1251/6392066) |
| 340 | Rigatelli, 2021 | Excluded: irrelevant topic | [Rigatelli G, Zuin M, Gianese F, Adami D, dell’Avvocata F, Barison S, et al. Ultrathin Biodegradable-Polymer Orsiro Drug-Eluting Stent Performance in Real Practice Challenging Settings. Cardiovascular Revascularization Medicine [Internet]. 2021 Sep [cited 2024 Sep 18];30:12–7. Available from: https://linkinghub.elsevier.com/retrieve/pii/S1553838920305765](https://linkinghub.elsevier.com/retrieve/pii/S1553838920305765) |
| 341 | Stomaci, 2021 | Excluded: wrong study design | [“52nd ANMCO Congress Abstracts E-Posters.” European Heart Journal Supplements 23, no. Supplement_C (August 25, 2021): C49–127. https://doi.org/10.1093/eurheartj/suab064.](https://doi.org/10.1093/eurheartj/suab064) |
| 342 | Franza, 2021 | Excluded: irrelevant topic | [“52nd ANMCO Congress Abstracts E-Posters.” European Heart Journal Supplements 23, no. Supplement_C (August 25, 2021): C49–127. https://doi.org/10.1093/eurheartj/suab064.](https://doi.org/10.1093/eurheartj/suab064) |
| 343 | Miura, 2021 | Excluded: irrelevant topic | [Miura T, Ueki Y, Senda K, Otagiri K, Tachibana T, Saigusa T, et al. Early vascular response of ultra-thin bioresorbable polymer sirolimus-eluting stents assessed by optical frequency domain imaging: the EVALUATION study. Cardiovasc Interv and Ther [Internet]. 2021 Jul [cited 2024 Sep 18];36(3):281–8. Available from: https://link.springer.com/10.1007/s12928-020-00689-9](https://link.springer.com/10.1007/s12928-020-00689-9) |
| 344 | Imamura, 2021 | Excluded: wrong study design | [Imamura Y, Kowatari R, Minakawa M, Fukuda I. Coronary artery bypass grafting after sternal turnover procedure and a review of the literature. J Card Surg [Internet]. 2021 Jun [cited 2024 Sep 18];36(6):2160–3. Available from: https://onlinelibrary.wiley.com/doi/10.1111/jocs.15478](https://onlinelibrary.wiley.com/doi/10.1111/jocs.15478) |
| 345 | Paradies, 2021 | Excluded: wrong publication type | [Paradies V, Waldeyer C, Laforgia PL, Clemmensen P, Smits PC. Completeness of revascularisation in acute coronary syndrome patients with multivessel disease. EuroIntervention [Internet]. 2021 Jun [cited 2024 Sep 18];17(3):193–201. Available from: https://eurointervention.pcronline.com/doi/10.4244/EIJ-D-20-00957](https://eurointervention.pcronline.com/doi/10.4244/EIJ-D-20-00957) |
| 346 | Zhao, 2021 | Excluded: wrong outcome | [Zhao X, Liu C, Zhou P, Zhaoxue S, Li J, Zhou J, et al. THROMBOSIS AND MAJOR BLEEDING RISK AFTER PRIMARY PERCUTANEOUS CORONARY INTERVENTION AMONG PATIENTS WITH MULTI-VESSELS CORONARY ARTERY DISEASE. Journal of the American College of Cardiology [Internet]. 2021 May [cited 2024 Sep 18];77(18):1036. Available from: https://linkinghub.elsevier.com/retrieve/pii/S0735109721023950](https://linkinghub.elsevier.com/retrieve/pii/S0735109721023950) |
| 347 | Gaba, 2021 | Excluded: wrong study design | [Gaba P, Gersh BJ, Ali ZA, Moses JW, Stone GW. Complete versus incomplete coronary revascularization: definitions, assessment and outcomes. Nat Rev Cardiol [Internet]. 2021 Mar [cited 2024 Sep 18];18(3):155–68. Available from: https://www.nature.com/articles/s41569-020-00457-5](https://www.nature.com/articles/s41569-020-00457-5) |
| 348 | Werner, 2021 | Excluded: wrong study design | [Werner N, Neumann FJ. Entwicklung kardiovaskuläre Medizin 2020: Ischämische Herzerkrankungen. Kardiologe [Internet]. 2021 Mar [cited 2024 Sep 18];15(2):92–100. Available from: http://link.springer.com/10.1007/s12181-021-00461-0](http://link.springer.com/10.1007/s12181-021-00461-0) |
| 349 | Tovar, 2021 | Excluded: wrong population | [Tovar Forero MN, Zanchin T, Masdjedi K, Van Zandvoort LJC, Kardys I, Zijlstra F, et al. Incidence and predictors of outcomes after a first definite coronary stent thrombosis. EuroIntervention [Internet]. 2020 Jul [cited 2024 Sep 18];16(4):e344–50. Available from: https://eurointervention.pcronline.com/doi/10.4244/EIJ-D-19-00219](https://eurointervention.pcronline.com/doi/10.4244/EIJ-D-19-00219) |
| 350 | Tang, 2020 | Excluded: wrong population | [Tang XF, Yao Y, Jia SD, Liu Y, Xu B, Yuan JQ. Clinical characteristics and prognosis in patients with premature coronary artery disease of different genders after intervention. European Heart Journal [Internet]. 2020 Nov 1 [cited 2024 Sep 18];41(Supplement_2):ehaa946.1315. Available from: https://academic.oup.com/eurheartj/article/doi/10.1093/ehjci/ehaa946.1315/6003931](https://academic.oup.com/eurheartj/article/doi/10.1093/ehjci/ehaa946.1315/6003931) |
| 351 | Polad, 2020 | Excluded: wrong population | [Polad J. One year clinical outcomes of contemporary PCI in patients with chronic coronary syndrome: experience from large scale e-ULTIMASTER registry. European Heart Journal [Internet]. 2020 Nov 1 [cited 2024 Sep 18];41(Supplement_2):ehaa946.1485. Available from: https://academic.oup.com/eurheartj/article/doi/10.1093/ehjci/ehaa946.1485/6005512](https://academic.oup.com/eurheartj/article/doi/10.1093/ehjci/ehaa946.1485/6005512) |
| 352 | De Filippo, 2020 | Excluded: wrong outcome | [De Filippo O, Gallone G, D’Ascenzo F, Peirone A, Castelli C, Leone AM, et al. Predictors of fractional flow reserve/instantaneous wave-free ratio discordance documented during functional coronary stenosis assessment: impact of tailored diagnostic cut-offs on long-term outcomes. European Heart Journal [Internet]. 2020 Nov 1 [cited 2024 Sep 18];41(Supplement_2):ehaa946.2483. Available from: https://academic.oup.com/eurheartj/article/doi/10.1093/ehjci/ehaa946.2483/6004080](https://academic.oup.com/eurheartj/article/doi/10.1093/ehjci/ehaa946.2483/6004080) |
| 353 | Matsoukis, 2020 | Excluded: wrong population | [Matsoukis I, Karanasos A, Patsa C, Anousakis-Vlachochristou N, Triantafyllou K, Kantzanou M, et al. Percutaneous coronary intervention with everolimus-eluting stents versus coronary artery bypass surgery in patients with stable angina and an isolated proximal left anterior descending artery disease. European Heart Journal [Internet]. 2020 Nov 1 [cited 2024 Sep 18];41(Supplement_2):ehaa946.2535. Available from: https://academic.oup.com/eurheartj/article/doi/10.1093/ehjci/ehaa946.2535/6004558](https://academic.oup.com/eurheartj/article/doi/10.1093/ehjci/ehaa946.2535/6004558) |
| 354 | Khan, 2020 | Excluded: wrong study design | [Khan MS, Khan AR, Khan AI, Seo M, Yasmin F, Usman MS, et al. Comparison of revascularization strategies in patients with acute coronary syndrome and multivessel coronary disease: A systematic review and network meta‐analysis. Cathet Cardio Intervent [Internet]. 2020 Oct [cited 2024 Sep 18];96(4). Available from: https://onlinelibrary.wiley.com/doi/10.1002/ccd.28855](https://onlinelibrary.wiley.com/doi/10.1002/ccd.28855) |
| 355 | Elgendy, 2020 | Excluded: wrong study design | [Elgendy IY, Mahtta D, Paniagua D. Multivessel PCI for Acute Myocardial Infarction: Where Do We Stand After The COMPLETE Trial? Curr Cardiol Rep [Internet]. 2020 Sep [cited 2024 Sep 18];22(9):97. Available from: https://link.springer.com/10.1007/s11886-020-01340-y](https://link.springer.com/10.1007/s11886-020-01340-y) |
| 356 | Bayraktaro, 2020 | Excluded: wrong outcome | [“ACVC Essentials 4 You.” European Heart Journal: Acute Cardiovascular Care 9, no. 2_suppl (August 2020): 1–196. https://doi.org/10.1177/2048872620937980.](https://doi.org/10.1177/2048872620937980) |
| 357 | Maffei, 2020 | Excluded: wrong study design | [Quagliana, A, S Grego, E Pasotti, M Araco, M Moccetti, G Pedrazzini, and T Moccetti. “HEART VALVE DISEASESP1 SEX AND GENDER DIFFERENCES IN TAVI PATIENTS: FROM CLINICAL PRESENTATION TO PROCEDURAL OUTCOMES.” European Heart Journal Supplements 22, no. Supplement_G (August 1, 2020): G57–210. https://doi.org/10.1093/eurheartj/suaa106.](https://doi.org/10.1093/eurheartj/suaa106) |
| 358 | Bainey, 2020 | Excluded: wrong population | [Bainey KR, Alemayehu W, Armstrong PW, Westerhout CM, Kaul P, Welsh RC. Long-Term Outcomes of Complete Revascularization With Percutaneous Coronary Intervention in Acute Coronary Syndromes. JACC: Cardiovascular Interventions [Internet]. 2020 Jul [cited 2024 Sep 18];13(13):1557–67. Available from: https://linkinghub.elsevier.com/retrieve/pii/S1936879820310220](https://linkinghub.elsevier.com/retrieve/pii/S1936879820310220) |
| 359 | Bellamoli, 2020 | Excluded: wrong population | [Bellamoli M, Marin F, Maritan L, Prati D, Tadiello E, Pesarini G, et al. New-onset extreme right axis deviation in acute myocardial infarction: clinical characteristics and outcomes. Journal of Electrocardiology [Internet]. 2020 May [cited 2024 Sep 18];60:60–6. Available from: https://linkinghub.elsevier.com/retrieve/pii/S0022073619309501](https://linkinghub.elsevier.com/retrieve/pii/S0022073619309501) |
| 360 | Rehman, 2020 | Excluded: irrelevant topic | [Rehman H, Patel A, McArdle M, George J, Escobar J, Sheth S, et al. CALLING FOR SUPPORT: EXTRACORPOREAL MEMBRANE OXYGENATION (ECMO) ASSISTED HIGH RISK PERCUTANEOUS CORONARY INTERVENTION OF MULTIVESSEL CORONARY ARTERY DISEASE. Journal of the American College of Cardiology [Internet]. 2020 Mar [cited 2024 Sep 18];75(11):3298. Available from: https://linkinghub.elsevier.com/retrieve/pii/S0735109720339255](https://linkinghub.elsevier.com/retrieve/pii/S0735109720339255) |
| 361 | Peteiro, 2020 | Excluded: wrong outcome | [Peteiro J, Bouzas-Mosquera A. Is there a role for ischemia detection after an acute myocardial infarction? WJC [Internet]. 2020 Jan 26 [cited 2024 Sep 18];12(1):1–6. Available from: https://www.wjgnet.com/1949-8462/full/v12/i1/1.htm](https://www.wjgnet.com/1949-8462/full/v12/i1/1.htm) |
| 362 | Alquran, 2020 | Excluded: wrong study design | [Alquran L, Patel A, Safi L, Patel A. A Rare Case of Multivessel SCAD Successfully Treated with Conservative Medical Management. Case Reports in Cardiology [Internet]. 2020 Jan 11 [cited 2024 Sep 18];2020:1–4. Available from: https://www.hindawi.com/journals/cric/2020/8468730/](https://www.hindawi.com/journals/cric/2020/8468730/) |
| 363 | Grieshaber, 2020 | Excluded: wrong publication type | [Grieshaber, P., I. Oswald, M. Albert, A. Sodah, P. Roth, A. Diegeler, D. Sedding, U. Franke, and A. Böning. “Staged Complete Hybrid Revascularization in Patients with Multivessel Disease and Acute Myocardial Infarction—A Prospective Angiographic and Clinical Study,” s-0040-1705398. Wiesbaden, 2020. https://doi.org/10.1055/s-0040-1705398.](https://doi.org/10.1055/s-0040-1705398) |
| 364 | Al-Hijji, 2020 | Excluded: wrong outcome | [Al-Hijji MA, Gulati R, Bell M, Kaplan RJ, Feind JL, Lewis BR, et al. Routine Continuous Electrocardiographic Monitoring Following Percutaneous Coronary Interventions. Circ: Cardiovascular Interventions [Internet]. 2020 Jan [cited 2024 Sep 18];13(1):e008290. Available from: https://www.ahajournals.org/doi/10.1161/CIRCINTERVENTIONS.119.008290](https://www.ahajournals.org/doi/10.1161/CIRCINTERVENTIONS.119.008290) |
| 365 | Chacko, 2020 | Excluded: wrong study design | [Chacko L, P. Howard J, Rajkumar C, Nowbar AN, Kane C, Mahdi D, et al. Effects of Percutaneous Coronary Intervention on Death and Myocardial Infarction Stratified by Stable and Unstable Coronary Artery Disease: A Meta-Analysis of Randomized Controlled Trials. Circ: Cardiovascular Quality and Outcomes [Internet]. 2020 Feb [cited 2024 Sep 18];13(2):e006363. Available from: https://www.ahajournals.org/doi/10.1161/CIRCOUTCOMES.119.006363](https://www.ahajournals.org/doi/10.1161/CIRCOUTCOMES.119.006363) |
| 366 | Gu, 2020 | Excluded: wrong study design | [Gu D, Qu J, Zhang H, Zheng Z. Revascularization for Coronary Artery Disease: Principle and Challenges. In: Wang M, editor. Coronary Artery Disease: Therapeutics and Drug Discovery [Internet]. Singapore: Springer Singapore; 2020 [cited 2024 Sep 18]. p. 75–100. (Advances in Experimental Medicine and Biology; vol. 1177). Available from: http://link.springer.com/10.1007/978-981-15-2517-9_3](http://link.springer.com/10.1007/978-981-15-2517-9_3) |
| 367 | Scarsini, 2020 | Excluded: wrong study design | [Scarsini R, Terentes-Printzios D, De Maria GL, Ribichini F, Banning A. Why, When and How Should Clinicians Use Physiology in Patients with Acute Coronary Syndromes? Interv Cardiol [Internet]. 2020 May 26 [cited 2024 Sep 18];15:e05. Available from: https://www.icrjournal.com/articleindex/icr.2019.26](https://www.icrjournal.com/articleindex/icr.2019.26) |
| 368 | Tiwari, 2020 | Excluded: irrelevant topic | [Tiwari A, Dwivedi SK, Chandra S, Chaudhary G, Sharma A, Sethi R, et al. Prevalence of single and double vessel disease in aVR ST-segment elevation (aVR-STE) and acute coronary syndrome (ACS) by coronary angiography. Indian Heart Journal [Internet]. 2019 Nov [cited 2024 Sep 18];71:S12–3. Available from: https://linkinghub.elsevier.com/retrieve/pii/S0019483219304754](https://linkinghub.elsevier.com/retrieve/pii/S0019483219304754) |
| 369 | Tang, 2019 | Excluded: irrelevant topic | [Tang XF, Yao Y, Jia SD, Liu Y, Xu B, Yuan JQ. Clinical characteristics and prognosis in patients with premature coronary artery disease of different genders after intervention. European Heart Journal [Internet]. 2020 Nov 1 [cited 2024 Sep 19];41(Supplement_2):ehaa946.1315. Available from: https://academic.oup.com/eurheartj/article/doi/10.1093/ehjci/ehaa946.1315/6003931](https://academic.oup.com/eurheartj/article/doi/10.1093/ehjci/ehaa946.1315/6003931) |
| 370 | Hao, 2019 | Excluded: wrong population | [Hao K, Takahashi J, Suda A, Sato K, Sugisawa J, Tsuchiya S, et al. P3575Clinical importance of fractional flow reserve in patients with organic coronary stenosis and vasospastic angina. European Heart Journal [Internet]. 2019 Oct 1 [cited 2024 Sep 18];40(Supplement_1):ehz745.0436. Available from: https://academic.oup.com/eurheartj/article/doi/10.1093/eurheartj/ehz745.0436/5596556](https://academic.oup.com/eurheartj/article/doi/10.1093/eurheartj/ehz745.0436/5596556) |
| 371 | Abou Jokh, 2019 | Excluded: wrong publication type | [Abou Jokh Casas C, Agra Bermejo R, Cordero A, Garcia Acuna JM, Rigueiro Veloso P, Iglesias Alvarez D, et al. P5503Long term prosnoctic benefit of complete revascularizaction in elderly non ST elevation myocardial infarction patients. European Heart Journal [Internet]. 2019 Oct 1 [cited 2024 Sep 18];40(Supplement_1):ehz746.0453. Available from: https://academic.oup.com/eurheartj/article/doi/10.1093/eurheartj/ehz746.0453/5597705](https://academic.oup.com/eurheartj/article/doi/10.1093/eurheartj/ehz746.0453/5597705) |
| 372 | Doshi, 2019 | Excluded: wrong outcome | [Doshi R, Singh A, Jauhar R, Meraj PM. Gender difference with the use of percutaneous left ventricular assist device in patients undergoing complex high-risk percutaneous coronary intervention: From pVAD Working Group. European Heart Journal: Acute Cardiovascular Care [Internet]. 2019 Jun [cited 2024 Sep 18];8(4):369–78. Available from: https://academic.oup.com/ehjacc/article/8/4/369-378/5943982](https://academic.oup.com/ehjacc/article/8/4/369-378/5943982) |
| 373 | Poudel, 2019 | Excluded: wrong drug | [“Poster Session 2.” European Journal of Preventive Cardiology 26, no. 1_suppl (June 2019): S71–127. https://doi.org/10.1177/2047487319860053.](https://doi.org/10.1177/2047487319860053) |
| 374 | Berlot, 2019 | Excluded: wrong study design | [Berlot B, De Francesco V, Harries I, Williams MGL, Mitrousi K, Bucciarelli-Ducci C. P372When a late presenter presents with a full house. European Heart Journal - Cardiovascular Imaging [Internet]. 2019 Jun 1 [cited 2024 Sep 18];20(Supplement_2):jez109.013. Available from: https://academic.oup.com/ehjcimaging/article/doi/10.1093/ehjci/jez109.013/5511149](https://academic.oup.com/ehjcimaging/article/doi/10.1093/ehjci/jez109.013/5511149) |
| 375 | Hanumanthu, 2019 | Excluded: wrong outcome | [“Abstracts.” Catheterization and Cardiovascular Interventions 93, no. S2 (May 2019). https://doi.org/10.1002/ccd.28216.](https://doi.org/10.1002/ccd.28216) |
| 376 | Borgaonkar, 2019 | Excluded: wrong study design | [“Abstracts.” Catheterization and Cardiovascular Interventions 93, no. S2 (May 2019). https://doi.org/10.1002/ccd.28216.](https://doi.org/10.1002/ccd.28216) |
| 377 | Benussi, 2019 | Excluded: wrong outcome | [Benussi B, Gatti G, Gripshi F, Biondi F, Porcari A, Ruggiero D, et al. Clinical Validation of a Coronary Surgery Technique That Minimizes Aortic Manipulation. The Annals of Thoracic Surgery [Internet]. 2019 Apr [cited 2024 Sep 18];107(4):1166–73. Available from: https://linkinghub.elsevier.com/retrieve/pii/S0003497518316412](https://linkinghub.elsevier.com/retrieve/pii/S0003497518316412) |
| 378 | Agra Bermejo, 2019 | Excluded: wrong publication type | [“Acute Cardiovascular Care 2019.” European Heart Journal: Acute Cardiovascular Care 8, no. 1_suppl (April 2019): 5–440. https://doi.org/10.1177/2048872619829424.](https://doi.org/10.1177/2048872619829424) |
| 379 | El-Hajj, 2019 | Excluded: wrong population | [El Hajj M, Hill A, Staub S, Fernandes V, Maran A. 100.51 Comparison of Rotational Versus Orbital Atherectomy for Multi-Vessel PCI in Patients With High-Risk Disease Who Are Turned Down for CABG. JACC: Cardiovascular Interventions [Internet]. 2019 Feb [cited 2024 Sep 18];12(4):S16. Available from: https://linkinghub.elsevier.com/retrieve/pii/S1936879819301165](https://linkinghub.elsevier.com/retrieve/pii/S1936879819301165) |
| 380 | Di Bacco, 2019 | Excluded: wrong study design | [Di Bacco L, Repossini A, Tespili M, Muneretto C, Bisleri G. Long-term follow-up of total arterial versus conventional and hybrid myocardial revascularization: A propensity score matched analysis. Cardiovascular Revascularization Medicine [Internet]. 2019 Jan [cited 2024 Sep 18];20(1):22–8. Available from: https://linkinghub.elsevier.com/retrieve/pii/S1553838918301350](https://linkinghub.elsevier.com/retrieve/pii/S1553838918301350) |
| 381 | Pender, 2019 | Excluded: wrong drug | [Pender P, Gibbs O, Faour A, Dang V, Hopkins A, Leung D, et al. Mechanical Circulatory Support for Semi – elective PCI in High-risk Patients with Extracorporeal Membranous Oxygenation (ECMO) Compared to Impella Heart Pump Device. Heart, Lung and Circulation [Internet]. 2019 [cited 2024 Sep 18];28:S414. Available from: https://linkinghub.elsevier.com/retrieve/pii/S1443950619312624](https://linkinghub.elsevier.com/retrieve/pii/S1443950619312624) |
| 382 | Noaman, 2019 | Excluded: wrong population | [Noaman S, Andrianopolous N, Brennan A, Reid C, Stub D, Biswas S, et al. Characteristics of Patients with Cardiogenic Shock Complicating Acute Coronary Syndrome and its Influence on Clinical Outcomes. Heart, Lung and Circulation [Internet]. 2019 [cited 2024 Sep 18];28:S388–9. Available from: https://linkinghub.elsevier.com/retrieve/pii/S1443950619311977](https://linkinghub.elsevier.com/retrieve/pii/S1443950619311977) |
| 383 | Berezhnoi, 2019 | Excluded: wrong population | [Berezhnoi K, Kokov L, Vanyukov A. Effects of complete revascularization on long-term treatment outcomes in patients with multivessel coronary artery disease over 80 years of age admitted for acute coronary syndrome. Cardiovasc Diagn Ther [Internet]. 2019 Aug [cited 2024 Sep 18];9(4):301–9. Available from: http://cdt.amegroups.com/article/view/23028/25176](http://cdt.amegroups.com/article/view/23028/25176) |
| 384 | Ridker, 2019 | Excluded: wrong drug | [“Late-Breaking Science Abstracts From the American Heart Association’s Scientific Sessions 2018 and Late-Breaking Abstracts in Resuscitation Science From the Resuscitation Science Symposium 2018.” Circulation 138, no. 25 (December 18, 2018). https://doi.org/10.1161/CIR.0000000000000636.](https://doi.org/10.1161/CIR.0000000000000636) |
| 385 | Mehta, 2018 | Excluded: wrong study design | [Mehta SR. Nonculprit Lesion Intervention. JACC: Cardiovascular Interventions [Internet]. 2018 Nov [cited 2024 Sep 18];11(22):2274–5. Available from: https://linkinghub.elsevier.com/retrieve/pii/S1936879818318995](https://linkinghub.elsevier.com/retrieve/pii/S1936879818318995) |
| 386 | Mahmud, 2018 | Excluded: wrong study design | [Mahmud E, Ben-Yehuda O. Percutaneous Coronary Intervention in Acute Coronary Syndrome. Journal of the American College of Cardiology [Internet]. 2018 Oct [cited 2024 Sep 18];72(17):2000–2. Available from: https://linkinghub.elsevier.com/retrieve/pii/S0735109718381397](https://linkinghub.elsevier.com/retrieve/pii/S0735109718381397) |
| 387 | Tamburino, 2018 | Excluded: wrong population | [Tamburino C, Briguori C, Jessurun GA, Reczuch K, Cortese B, Maillard L, et al. TCT-329 Prospective Evaluation of Drug Eluting Self Apposing Stent for the Treatment of Unprotected Left Main Coronary Artery Disease: 1-Year Results of the TRUNC Study. Journal of the American College of Cardiology [Internet]. 2018 Sep [cited 2024 Sep 18];72(13):B134–5. Available from: https://linkinghub.elsevier.com/retrieve/pii/S0735109718374813](https://linkinghub.elsevier.com/retrieve/pii/S0735109718374813) |
| 388 | Kobayashi, 2018 | Excluded: wrong outcome | [Kobayashi Y, Lønborg J, Jong A, Nishi T, De Bruyne B, Høfsten DE, et al. Prognostic Value of the Residual SYNTAX Score After Functionally Complete Revascularization in ACS. Journal of the American College of Cardiology [Internet]. 2018 Sep [cited 2024 Sep 18];72(12):1321–9. Available from: https://linkinghub.elsevier.com/retrieve/pii/S0735109718355918](https://linkinghub.elsevier.com/retrieve/pii/S0735109718355918) |
| 389 | Kanabar, 2018 | Excluded: wrong population | [Kanabar K, Mehrotra S, P. R. Ostial left main coronary artery chronic total occlusion presenting as chronic stable angina. Indian Heart Journal [Internet]. 2018 Sep [cited 2024 Sep 18];70(5):745–9. Available from: https://linkinghub.elsevier.com/retrieve/pii/S0019483217308453](https://linkinghub.elsevier.com/retrieve/pii/S0019483217308453) |
| 390 | Farouk, 2018 | Excluded: wrong outcome | Farouk, A. and Nadra, B. Prognostic value of SYNTAX score ii in patients with non-ST elevation myocardial infarction and multivessel disease undergoing percutaneous coronary intervention. J. Invasive Cardiol. 2018 |
| 391 | Khripun, 2018 | Excluded: wrong outcome | [Khripun AV, Malevannyy MV, Chesnikova AI, Kastanayan AA, Kulikovskikh YAV, Gridneva UU. P5608Outcomes of PCI with bioresorbable vascular scaffolds in patients with acute coronary syndrome. European Heart Journal [Internet]. 2018 Aug 1 [cited 2024 Sep 18];39(suppl_1). Available from: https://academic.oup.com/eurheartj/article/doi/10.1093/eurheartj/ehy566.P5608/5081695](https://academic.oup.com/eurheartj/article/doi/10.1093/eurheartj/ehy566.P5608/5081695) |
| 392 | Fortuni, 2018 | Excluded: wrong study design | [Fortuni F, Rolando M, Foglia D, Crimi G, Leonardi S, Ferlini M, et al. P5103There is no time like the present - a meta-analysis on complete versus culprit-only revascularization in acute coronary syndrome. European Heart Journal [Internet]. 2018 Aug 1 [cited 2024 Sep 18];39(suppl_1). Available from: https://academic.oup.com/eurheartj/article/doi/10.1093/eurheartj/ehy566.P5103/5082842](https://academic.oup.com/eurheartj/article/doi/10.1093/eurheartj/ehy566.P5103/5082842) |
| 393 | Meija, 2018 | Excluded: wrong outcome | [Mejia Renteria HD, Perez-Vizcayno MJ, Lee JM, Van Der Hoeven NW, De Waard GA, Nombela-Franco L, et al. P4627Assessment of the coronary microcirculation remote to an infarcted territory: insights for FFR-guided coronary revascularization of non-culprit vessels in the subacute phase of a myocardial infarction. European Heart Journal [Internet]. 2018 Aug 1 [cited 2024 Sep 18];39(suppl_1). Available from: https://academic.oup.com/eurheartj/article/doi/10.1093/eurheartj/ehy563.P4627/5081494](https://academic.oup.com/eurheartj/article/doi/10.1093/eurheartj/ehy563.P4627/5081494) |
| 394 | Bertaina, 2018 | Excluded: wrong study design | [Bertaina MB, Ferraro IF, Omede’ PO, Conrotto FC, Cavender MC, Claessen BEC, et al. P2731Complete or culprit only revascularization in patients with multivessel disease presenting with cardiogenic shock: a meta-analysis of RCT and adjusted observational results. European Heart Journal [Internet]. 2018 Aug 1 [cited 2024 Sep 18];39(suppl_1). Available from: https://academic.oup.com/eurheartj/article/doi/10.1093/eurheartj/ehy565.P2731/5083188](https://academic.oup.com/eurheartj/article/doi/10.1093/eurheartj/ehy565.P2731/5083188) |
| 395 | Moura-Ferreira, 2018 | Excluded: wrong outcome | [Moura-Ferreira S, Ladeiras-Lopes R, MBala D, Rodrigues A, Braga P, Gama V. The role of Impella in high-risk percutaneous coronary intervention. Revista Portuguesa de Cardiologia [Internet]. 2018 Jul [cited 2024 Sep 18];37(7):623.e1-623.e4. Available from: https://linkinghub.elsevier.com/retrieve/pii/S0870255116303833](https://linkinghub.elsevier.com/retrieve/pii/S0870255116303833) |
| 396 | Elorriaga Madari, 2018 | Excluded: irrelevant topic | [“Acute Cardiovascular Care 2018.” European Heart Journal: Acute Cardiovascular Care 7, no. 1_suppl (March 2018): 4–361. https://doi.org/10.1177/2048872617751067.](https://doi.org/10.1177/2048872617751067) |
| 397 | Park, 2018 | Excluded: wrong outcome | [Park KH, Jeong MH, Hong YJ, Ahn Y, Kim HK, Koh YY, et al. Effectiveness and Safety of Biolimus A9TM-Eluting stEnt in Patients with AcUTe Coronary sYndrome; A Multicenter, Observational Study (BEAUTY Study). Yonsei Med J [Internet]. 2018 [cited 2024 Sep 18];59(1):72. Available from: https://eymj.org/DOIx.php?id=10.3349/ymj.2018.59.1.72](https://eymj.org/DOIx.php?id=10.3349/ymj.2018.59.1.72) |
| 398 | Hawranek, 2018 | Excluded: wrong outcome | [Hawranek M, Desperak P, Gąsior P, Desperak A, Lekston A, Gąsior M. Early and long-term outcomes of complete revascularization with percutaneous coronary intervention in patients with multivessel coronary artery disease presenting with non-ST-segment elevation acute coronary syndromes. pwki [Internet]. 2018 [cited 2024 Sep 18];14(1):32–41. Available from: https://www.termedia.pl/doi/10.5114/aic.2018.74353](https://www.termedia.pl/doi/10.5114/aic.2018.74353) |
| 399 | Nochioka, 2017 | Excluded: wrong population | [Nochioka, Kotaro, Tor Biering-Sørensen, Kim Wadt Hansen, Rikke Sørensen, Sune Pedersen, Peter Godsk Jørgensen, Allan Iversen, et al. “Long-Term Outcomes in Patients with Rheumatologic Disorders Undergoing Percutaneous Coronary Intervention: A BAsel Stent Kosten-Effektivitäts Trial-PROspective Validation Examination (BASKET-PROVE) Sub-Study.” European Heart Journal: Acute Cardiovascular Care 6, no. 8 (December 2017): 778–86. https://doi.org/10.1177/2048872616649860.](https://doi.org/10.1177/2048872616649860) |
| 400 | Wickramarachchi, 2017 | Excluded: wrong population | [Wickramarachchi, Upul, Natasha H. Corballis, Clint A. Maart, Timothy J. Gilbert, and Simon C. Eccleshall. “24 Drug Coated Balloon-Only Angioplasty in Chronic Total Occlusions, a UK Single Centre Experience.” Heart 103, no. Suppl 7 (December 1, 2017): A11–A11. https://doi.org/10.1136/heartjnl-2017-BCIS.24.](https://doi.org/10.1136/heartjnl-2017-BCIS.24) |
| 401 | Sakhov, 2017 | Excluded: wrong drug | [Sakhov, Orazbek, Murat Kuzhukeyev, Almat Kodasbayev, Aizhan Kulanbayeva, and Saule Dairbekova. “TCT-407 Treatment of Acute Coronary Syndrome Patients with a Sirolimus-Eluting Bioresorbable Polymer-Coated Stent: 1-Year Clinical Outcomes from the e-Ultimaster Registry (on Behalf of the e-Ultimaster Investigators).” Journal of the American College of Cardiology 70, no. 18 (October 2017): B168. https://doi.org/10.1016/j.jacc.2017.09.507.](https://doi.org/10.1016/j.jacc.2017.09.507) |
| 402 | Telayna, 2017 | Excluded: wrong population | [“Best of the Best Poster Presentation Abstracts.” Catheterization and Cardiovascular Interventions 89, no. S2 (May 2017). https://doi.org/10.1002/ccd.27053.](https://doi.org/10.1002/ccd.27053) |
| 403 | Gaffar, 2017 | Excluded: wrong study design | [Gaffar, Rouan, Bettina Habib, Kristian B. Filion, Pauline Reynier, and Mark J. Eisenberg. “Optimal Timing of Complete Revascularization in Acute Coronary Syndrome: A Systematic Review and Meta‐Analysis.” Journal of the American Heart Association 6, no. 4 (April 5, 2017): e005381. https://doi.org/10.1161/JAHA.116.005381.](https://doi.org/10.1161/JAHA.116.005381) |
| 404 | Smits, 2017 | Excluded: wrong population | [Smits, Pieter C., Mohamed Abdel-Wahab, Franz-Josef Neumann, Bianca M. Boxma-de Klerk, Ketil Lunde, Carl E. Schotborgh, Zsolt Piroth, et al. “Fractional Flow Reserve–Guided Multivessel Angioplasty in Myocardial Infarction.” New England Journal of Medicine 376, no. 13 (March 30, 2017): 1234–44. https://doi.org/10.1056/NEJMoa1701067.](https://doi.org/10.1056/NEJMoa1701067) |
| 405 | Layland, 2017 | Excluded: wrong outcome | [Layland, Jamie John, Jaclyn Carberry, Vanessa T.Y. May, Matthew Lee, Hany Eteiba, Mitchell Lindsay, Mark Petrie, et al. “DECISION MAKING USING FRACTIONAL FLOW RESERVE IS ASSOCIATED WITH LOWER RATES OF INCOMPLETE REVASCULARIZATION COMPARED WITH ANGIOGRAPHIC GUIDED DECISION MAKING AMONGST PATIENTS WITH NSTEMI.” Journal of the American College of Cardiology 69, no. 11 (March 2017): 1057. https://doi.org/10.1016/S0735-1097(17)34446-7.](https://doi.org/10.1016/S0735-1097(17)34446-7) |
| 406 | Schmidt, 2017 | Excluded: wrong outcome | [Schmidt, Torrey Richard, Mark Kozak, Ian Gilchrist, and Andrew Foy. “ASSESSING INTER-RATER AGREEMENT OF EXPERIENCED INTERVENTIONAL CARDIOLOGISTS ON CULPRIT VESSEL IN PATIENTS WITH MULTIVESSEL DISEASE AND ACUTE NON-STEMI.” Journal of the American College of Cardiology 69, no. 11 (March 2017): 1155. https://doi.org/10.1016/S0735-1097(17)34544-8.](https://doi.org/10.1016/S0735-1097(17)34544-8) |
| 407 | Kim, 2017 | Excluded: wrong outcome | [Kim, Yong Hoon, Ae-Young Her, Seung-Woon Rha, Byoung Geol Choi, Minsuk Shim, Se Yeon Choi, Jae Kyeong Byun, et al. “Routine Angiographic Follow-up versus Clinical Follow-up in Patients with Multivessel Coronary Artery Diseases Following Percutaneous Coronary Intervention with Drug-Eluting Stents: A Nested Case–Control Study within a Korean Population.” Coronary Artery Disease 28, no. 4 (June 2017): 307–14. https://doi.org/10.1097/MCA.0000000000000479.](https://doi.org/10.1097/MCA.0000000000000479) |
| 408 | Noaman, 2017 | Excluded: wrong outcome | [Noaman, S., N. Andrianopoulos, A. Brennan, C. Reid, D. Stub, S. Biswas, A. Walton, et al. “Clinical Outcomes of Cardiogenic Shock Complicating Acute Coronary Syndrome Treated with Percutaneous Coronary Intervention.” Heart, Lung and Circulation 26 (2017): S72. https://doi.org/10.1016/j.hlc.2017.06.066.](https://doi.org/10.1016/j.hlc.2017.06.066) |
| 409 | Ruparelia, 2017 | Excluded: irrelevant topic | [Ruparelia, Neil, Hiroyoshi Kawamoto, Davide Capodanno, Tommaso Gori, Holger Nef, Julinda Mehilli, Maciej Lesiak, et al. “TCT-401 Bioresorbable Scaffolds And Thrombosis: Insights From The GHOST-EU (Gauging Coronary Healing with biOresorbable Scaffolding plaTforms in Europe) Registry.” Journal of the American College of Cardiology 68, no. 18 (November 2016): B163. https://doi.org/10.1016/j.jacc.2016.09.536.](https://doi.org/10.1016/j.jacc.2016.09.536) |
| 410 | Wykrzykowska, 2017 | Excluded: wrong drug | [Wykrzykowska, Joanna, Robin Kraak, Joëlle Elias, Ivo van Dongen, Sjoerd Hofma, Rene Schaaf, Karin Arkenbout, et al. TCT-430 Baseline and Procedural Characteristics in the Amsterdam Investigator-initiateD Randomized Absorb Trial. Journal of the American College of Cardiology. Vol. 68, 2016. https://doi.org/10.1016/j.jacc.2016.09.565.](https://doi.org/10.1016/j.jacc.2016.09.565) |
| 411 | Shugushev, 2017 | Excluded: wrong outcome | [“Acute Cardiovascular Care 2016.” European Heart Journal: Acute Cardiovascular Care 5, no. 1_suppl (October 2016): 4–440. https://doi.org/10.1177/2048872616663431.](https://doi.org/10.1177/2048872616663431) |
| 412 | Silenzi, 2016 | Excluded: wrong outcome | [Silenzi, Simona, Pierfrancesco Grossi, Luca Mariani, Chiara Fraccaro, Fabio Vagnarelli, and Luciano Moretti. “Effect on Clinical Restenosis of an Ultra‐Thin‐Strut Bare Metal Cobalt‐Chromium Stent Versus a Thin‐Strut Stainless Steel Stent.” Journal of Interventional Cardiology 29, no. 3 (June 2016): 300–310. https://doi.org/10.1111/joic.12300.](https://doi.org/10.1111/joic.12300) |
| 413 | Ielasi, 2016 | Excluded: wrong drug | [“Sunday 28 August 2016.” European Heart Journal 37, no. suppl 1 (August 1, 2016): 191–598. https://doi.org/10.1093/eurheartj/ehw432.](https://doi.org/10.1093/eurheartj/ehw432) |
| 414 | Yoon, 2016 | Excluded: wrong outcome | [“Sunday 28 August 2016.” European Heart Journal 37, no. suppl 1 (August 1, 2016): 191–598. https://doi.org/10.1093/eurheartj/ehw432.](https://doi.org/10.1093/eurheartj/ehw432) |
| 415 | Silenzi, 2016 | Excluded: wrong outcome | [Silenzi, Simona, Pierfrancesco Grossi, Luca Mariani, Chiara Fraccaro, Fabio Vagnarelli, and Luciano Moretti. “Effect on Clinical Restenosis of an Ultra‐Thin‐Strut Bare Metal Cobalt‐Chromium Stent Versus a Thin‐Strut Stainless Steel Stent.” Journal of Interventional Cardiology 29, no. 3 (June 2016): 300–310. https://doi.org/10.1111/joic.12300.](https://doi.org/10.1111/joic.12300) |
| 416 | Sumaya, 2016 | Excluded: wrong outcome | Sumaya, W, et al. Percutaneous revascularisation of the collateral supply vessel for patients with multivessel disease and a chronically occluded vessel. EuroIntervention. 2016 |
| 417 | Freitas, 2016 | Excluded: wrong outcome | Freitas, P et al. CABG vs. PCI in NSTEMI patients with multivessel disease: A propensity score analysis. EuroIntervention. 2016 |
| 418 | Camacho Freire, 2016 | Excluded: wrong outcome | Camacho Freire, S.J, et al. PCI in patients at high risk of bleeding with polymer-coated stent without biolimus (BioFreedom): Multicentre all-comers registry. EuroIntervention. 2016 |
| 419 | Vassilev, 2016 | Excluded: wrong publication type | Vassilev, D, et al. Occlusion of secondary side branches after stenting coronary bifurcation lesions predicts recurrence of symptoms of angina or congestive heart failure at midterm follow-up (up to 60 months) from intracoronary electrocardiogram (ECG) and myonecrosis after bifurcation stenting (COSIBRIA&CO) (ClinicalTrials.Gov Identifier:NCT01268228). EuroIntervention. 2016 |
| 420 | Palazuelos, 2016 | Excluded: irrelevant topic | Palazuelos, J, et al. Complications in unstable patients referred to complex PCI when rotational atherectomy and bivaluridin are used. EuroIntervention. 2016 |
| 421 | Chevalier, 2016 | Excluded: irrelevant topic | Chevalier, B, et al. Assessment of strut coverage of biodegradable polymer DES at one, two and three months after stent implantation by optical frequency domain imaging: Final results of DISCOVERY 1TO3 study. EuroIntervention. 2016 |
| 422 | Dani, 2016 | Excluded: irrelevant topic | Dani, S., et al. Clinical outcomes of patients treated with a novel abluminal-coated sirolimus-eluting stent: An overall analysis and a subgroup analysis from an all-comer en-ABL e-registry. EuroIntervention. 2016 |
| 423 | Zhou, 2016 | Excluded: wrong outcome | [Zhou, Li, Hui Chen, Wei-Ping Li, Hong-Li Gao, Dong-Bao Li, Hui-Qiang Zhao, Dao-Kuo Yao, and Hong-Wei Li. “Short- and Long-Term Outcomes in Patients with Connective Tissue Diseases Undergoing Percutaneous Coronary Intervention.” Chinese Medical Journal 129, no. 7 (April 5, 2016): 804–8. https://doi.org/10.4103/0366-6999.178956.](https://doi.org/10.4103/0366-6999.178956) |
| 424 | Milewski, 2016 | Excluded: irrelevant topic | [Milewski, Krzysztof, Paweł Gąsior, Stefan Samborski, Piotr P. Buszman, Aleksandra Błachut, Adam Wojtaszczyk, Adam Młodziankowski, et al. “Evaluation of Safety and Efficacy of NexGen – an Ultrathin Strut and Hybrid Cell Design Cobalt-Chromium Bare Metal Stent Implanted in a Real Life Patient Population – the Polish NexGen Registry.” Advances in Interventional Cardiology 3 (2016): 217–23. https://doi.org/10.5114/aic.2016.61642.](https://doi.org/10.5114/aic.2016.61642) |
| 425 | Kini, 2015 | Excluded: irrelevant topic | [Kini, Prayaag. “Clinical, Demographic and Angiographic Profile in Diabetic Patients Presenting with Acute Coronary Syndromes – A Tertiary Care Center Study.” Indian Heart Journal 67 (December 1, 2015): S47. https://doi.org/10.1016/j.ihj.2015.10.113.](https://doi.org/10.1016/j.ihj.2015.10.113) |
| 426 | Banerjee, 2015 | Excluded: irrelevant topic | [Banerjee, Sunip. “Provisional Observation of FFR Outcome Proves Utility in Ambiguous Vessel Abnormality.” Indian Heart Journal 67 (December 1, 2015): S67–68. https://doi.org/10.1016/j.ihj.2015.10.168.](https://doi.org/10.1016/j.ihj.2015.10.168) |
| 427 | Qamar, 2015 | Excluded: wrong population | [Qamar, Arman, and Deepak L. Bhatt. “Culprit-Only vs. Complete Revascularization During ST-Segment Elevation Myocardial Infarction.” Progress in Cardiovascular Diseases 58, no. 3 (November 2015): 260–66. https://doi.org/10.1016/j.pcad.2015.07.006.](https://doi.org/10.1016/j.pcad.2015.07.006) |
| 428 | Brener, 2015 | Excluded: wrong publication type | [“11th International Congress on Coronary Artery Disease (ICCAD), Florence, Italy November 29 - December 2, 2015: Abstracts.” Cardiology 132, no. 1 (2015): 1–312. https://doi.org/10.1159/000442375.](https://doi.org/10.1159/000442375) |
| 429 | Noad, 2015 | Excluded: irrelevant topic | [Noad, R., C. Hanratty, and S. Walsh. “7 Initial Experience of Bioabsorbable Polymer Everolimus-Eluting Stents in High Risk Patients.” Heart 101, no. Suppl 5 (September 1, 2015): A4–A4. https://doi.org/10.1136/heartjnl-2015-308621.7.](https://doi.org/10.1136/heartjnl-2015-308621.7) |
| 430 | George, 2015 | Excluded: wrong publication type | [George, Sudhakar, and Patrick A. Calvert. “Ischaemic Heart Disease – a Selected Review of Recent Developments.” Current Opinion in Cardiology 30, no. 6 (November 2015): 657–62. https://doi.org/10.1097/HCO.0000000000000230.](https://doi.org/10.1097/HCO.0000000000000230) |
| 431 | Madeira, 2015 | Excluded: irrelevant topic | [“Acute Cardiovascular Care 2015.” European Heart Journal: Acute Cardiovascular Care 4, no. 1_suppl (October 2015): 5–348. https://doi.org/10.1177/2048872615599730.](https://doi.org/10.1177/2048872615599730) |
| 432 | Muneretto, 2015 | Excluded: wrong study design | [“Sunday, 30 August 2015.” European Heart Journal 36, no. suppl 1 (August 2015): 163–508. https://doi.org/10.1093/eurheartj/ehv399.](https://doi.org/10.1093/eurheartj/ehv399) |
| 433 | Baptista, 2015 | Excluded: wrong publication type | [“Monday, 31 August 2015.” European Heart Journal 36, no. suppl 1 (August 2015): 509–847. https://doi.org/10.1093/eurheartj/ehv400.](https://doi.org/10.1093/eurheartj/ehv400) |
| 434 | Duarte Rodrigues, 2015 | Excluded: wrong outcome | [“Monday, 31 August 2015.” European Heart Journal 36, no. suppl 1 (August 2015): 509–847. https://doi.org/10.1093/eurheartj/ehv400.](https://doi.org/10.1093/eurheartj/ehv400) |
| 435 | Duarte Rodrigues, 2015 | Excluded: wrong outcome | [“Abstracts.” European Journal of Heart Failure 17, no. S1 (May 2015): 5–441. https://doi.org/10.1002/ejhf.277.](https://doi.org/10.1002/ejhf.277) |
| 436 | Ricardo Antonio Mori, 2015 | Excluded: wrong population | [“Poster Session 2 – Afternoon.” European Journal of Preventive Cardiology 22, no. 1_suppl (May 2015): S46–76. https://doi.org/10.1177/2047487315586734.](https://doi.org/10.1177/2047487315586734) |
| 437 | Shin, 2015 | Excluded: wrong outcome | Shin, D.I. et al. Comparison of the 48-month clinical outcomes between patients with non-ST-elevation myocardial infarction who had an occluded culprit artery and those with ST-elevation myocardial infarction. EuroIntervention. 2015 |
| 438 | Hildick-Smith, 2015 | Excluded: wrong outcome | Hildick-Smith, D. et al. Long-term safety and efficacy of biolimus-eluting coronary stents in an unselected patient population: Final 3-year report of the large, multicentre e-BioMatrix registry. EuroIntervention. 2015 |
| 439 | Camacho Freire, 2015 | Excluded: irrelevant topic | Camacho Freire, S.J. et al. Treatment of bifurcation lesions with a novel dedicated drug-eluting self-expanding stent (AXXESS): Single centre first experience. EuroIntervention. 2015 |
| 440 | Christ, 2015 | Excluded: irrelevant topic | Christ, G. et al. Everolimus-eluting bioabsorbable vascular scaffold and personalised platelet inhibition: No scaffold thrombosis in a single-centre registry with 223 consecutive patients. EuroIntervention. 2015 |
| 441 | Duarte-Rodrigues, 2015 | Excluded: wrong outcome | Duarte-Rodrigues, J. et al. Hybrid Coronary Revascularisation in 100 patients with multi-vessel disease: What can we expect?. EuroIntervention. 2015 |
| 442 | Polavarapu, 2015 | Excluded: wrong outcome | Polavarapu, R.S. et al. Clinical outcomes after implantation of bioabsorbable polymer coated sirolimus-eluting stent in multivessel stenting: 6-month follow-up data from the MULTIDOS study. EuroIntervention. 2015 |
| 443 | Sandoval, 2015 | Excluded: wrong study design | [Sandoval, Yader, Emmanouil S. Brilakis, Mariana Canoniero, Demetris Yannopoulos, and Santiago Garcia. “Complete Versus Incomplete Coronary Revascularization of Patients With Multivessel Coronary Artery Disease.” Current Treatment Options in Cardiovascular Medicine 17, no. 3 (March 2015): 8. https://doi.org/10.1007/s11936-015-0366-1.](https://doi.org/10.1007/s11936-015-0366-1) |
| 444 | Agarwal, 2015 | Excluded: irrelevant topic | [“Abstracts of Posters Presented at the 2015 Annual Meeting of the International Anesthesia Research Society Honolulu, Hawaii March 21–24, 2015.” Anesthesia & Analgesia 120, no. 3S_Suppl (March 2015): 1–555. https://doi.org/10.1213/01.ane.0000470325.07465.0f.](https://doi.org/10.1213/01.ane.0000470325.07465.0f) |
| 445 | Kobayashi, 2015 | Excluded: wrong outcome | [Kobayashi, Akihiro, Naoki Misumida, Yumiko Kanei, and John Fox. “CRT-110 Elevated Left Ventricular End-Diastolic Pressure Predicts a Higher Rate of In-Hospital and 30-Day Major Adverse Cardiac Events in Patients with Non-ST Elevation Myocardial Infarction.” JACC: Cardiovascular Interventions 8, no. 2_Supplement (February 2015): S11–S11. https://doi.org/10.1016/j.jcin.2014.12.040.](https://doi.org/10.1016/j.jcin.2014.12.040) |
| 446 | Misumida, 2015 | Excluded: wrong outcome | [Misumida, Naoki, Akihiro Kobayashi, Madeeha Saeed, John T. Fox, and Yumiko Kanei. “Prevalence and Outcomes of Non-ST-Segment Elevation Myocardial Infarction Resulting from Stent Thrombosis.” Cardiovascular Revascularization Medicine: Including Molecular Interventions 16, no. 4 (June 2015): 204–7. https://doi.org/10.1016/j.carrev.2015.03.002.](https://doi.org/10.1016/j.carrev.2015.03.002) |
| 447 | Pendyala, 2015 | Excluded: irrelevant topic | Pendyala, Lakshmana, Joshua Loh, Hironori Kitabata, Sa’ar Minha, Fang Chen, Rebecca Torguson, William Suddath, Lowell Satler, Augusto Pichard, and Ron Waksman. “Clinical Impact of Second-Generation Everolimus-Eluting Stents Compared with First-Generation Drug-Eluting Stents in Diabetic Patients Undergoing Multivessel Percutaneous Coronary Intervention.” *The Journal of Invasive Cardiology* 27, no. 6 (June 2015): 263–68. |
| 448 | Zhang, 2015 | Excluded: irrelevant topic | Zhang, Y. J., W. Wu, D. R. Pan, B. Xu, J. Kan, Y. X. Chen, S. Pang, et al. “Feasibility of a Novel Abluminal Groove-Filled Biodegradable Polymer Sirolimus-Eluting Stent in Patients with Complex Anatomical and Clinical Scenarios.” *Minerva Cardioangiologica* 63, no. 1 (February 2015): 1–9. |
| 449 | Shakhov, 2015 | Excluded: irrelevant topic | [Shakhov, E.B., D.V. Volkov, E.B. Petrova, E.S. Timoschenko, S.G. Erofeyeva, and A.A. Nekrasov. “Novel Technique of Endovascular Retrograde Circulatory Support in Patients with Acute Coronary Syndrome: Emphasis on Myocardium Functional State.” Sovremennye Tehnologii v Medicine 7, no. 4 (December 2015): 14–20. https://doi.org/10.17691/stm2015.7.4.02.](https://doi.org/10.17691/stm2015.7.4.02) |
| 450 | Bekler, 2015 | Excluded: irrelevant topic | [Bekler, Adem, Erhan Tenekecioglu, Gokhan Erbag, Ahmet Temiz, Burak Altun, Ahmet Barutcu, Emine Gazi, Fahri Gunes, and Mustafa Yilmaz. “Relationship between Red Cell Distribution Width and Long-Term Mortality in Patients with Non-ST Elevation Acute Coronary Syndrome.” The Anatolian Journal of Cardiology 15, no. 8 (August 21, 2015): 634–39. https://doi.org/10.5152/akd.2014.5645.](https://doi.org/10.5152/akd.2014.5645) |
| 451 | Maillard, 2015 | Excluded: irrelevant topic | [Maillard, Luc, Nicolas Barra, Jacques Billé, Marie Rose Clergeau, Patrick Joly, Marc Silvestri, Alain Tavildari, and François Vochelet. “0133: The First Human Experience with Novel Nano Surface Modified Cobra PzF Stent.” Archives of Cardiovascular Diseases Supplements, 25es Journées Européennes de la Société Française de Cardiologie – 14-17 janvier 2015 –Palais des Congrès, Paris, 7, no. 1 (January 1, 2015): 4. https://doi.org/10.1016/S1878-6480(15)71497-7.](https://doi.org/10.1016/S1878-6480(15)71497-7) |
| 452 | Geraci, 2014 | Excluded: wrong publication type | [“C1-C144.” Giornale Italiano di Cardiologia, no. 201 (2014). https://doi.org/10.1714/1642.17988.](https://doi.org/10.1714/1642.17988) |
| 453 | Kobayashi, 2014 | Excluded: irrelevant topic | [Kobayashi, A., N. Misumida, and Y. Kanei. “THE INDEPENDENCY OF ST ELEVATION IN LEAD AVR AND ST DEPRESSION AS A PREDICTOR FOR MULTI-VESSEL DISEASE IN PATIENTS WITH NON-ST ELEVATION MYOCARDIAL INFARCTION.” Canadian Journal of Cardiology 30, no. 10 (October 1, 2014): S60–61. https://doi.org/10.1016/j.cjca.2014.07.032.](https://doi.org/10.1016/j.cjca.2014.07.032) |
| 454 | Ding, 2014 | Excluded: wrong outcome | [Ding, L., M. Yu, and A. Chan. “1-YEAR TARGET VESSEL REVASCULARIZATION AFTER PERCUTANEOUS CORONARY INTERVENTION IN BC (2010-2012).” Canadian Journal of Cardiology 30, no. 10 (October 1, 2014): S89–90. https://doi.org/10.1016/j.cjca.2014.07.090.](https://doi.org/10.1016/j.cjca.2014.07.090) |
| 455 | Kobayashi, 2014 | Excluded: irrelevant topic | [Kobayashi, Akihiro, Naoki Misumida, and Yumiko Kanei. “TCT-6 Positive T-Wave Amplitude In Lead AVR As A Predictor For A Higher Rate Of In-Hospital Coronary Artery Bypass Graft In Patients With Non-ST Elevation Myocardial Infarction.” Journal of the American College of Cardiology 64, no. 11_Supplement (September 16, 2014): B2–B2. https://doi.org/10.1016/j.jacc.2014.07.030.](https://doi.org/10.1016/j.jacc.2014.07.030) |
| 456 | Gasior, 2014 | Excluded: wrong population | [Gasior, Pawel, Michal Hawranek, Mateusz Tajstra, Jacek Piegza, ński Janusz Szkodzi, Piotr Desperak, Cislak Aneta, Andrzej Lekston, Mariusz Gasior, and Lech Polonski. “TCT-220 Impact of Chronic Total Occlusion Localization on 12-Month Mortality in Patients with Non-ST Segment Elevation Myocardial Infarction Treated with Percutaneous Coronary Intervention.” Journal of the American College of Cardiology 64, no. 11_Supplement (September 16, 2014): B64–B64. https://doi.org/10.1016/j.jacc.2014.07.261.](https://doi.org/10.1016/j.jacc.2014.07.261) |
| 457 | Wanha, 2014 | Excluded: irrelevant topic | [Wanha, Wojciech M., Damian Kawecki, Beata Ochala, Tomasz Roleder, Beata Morawiec, Janusz Dola, Aleksandra Pluta, et al. “TCT-575 Comparison of Clinical Outcomes and Safety of First and Second-Generation DES in Women and Men [Katowice-Zabrze Registry on First and Second Generation DES].” Journal of the American College of Cardiology 64, no. 11_Supplement (September 16, 2014): B167–B167. https://doi.org/10.1016/j.jacc.2014.07.638.](https://doi.org/10.1016/j.jacc.2014.07.638) |
| 458 | Fernandez-Pereira, 2014 | Excluded: wrong outcome | [Fernandez, -Pereira Carlos, Ignacio Rifourcat, Juan Mieres, Carlos Haiek, Omar Santaera, Miguel Larribau, Juan Lloberas, et al. “TCT-577 Revascularization Strategies for Patients with Multiple Vessel Coronary Disease and Unprotected Left Main. Mid Term Results from a Prospective, Multicenter and Controlled Argentina Registry with a Cobalt- Chromium Rapamycin Eluting Stent, FIREBIRD 2® (ERACI 4).” Journal of the American College of Cardiology 64, no. 11_Supplement (September 16, 2014): B167–68. https://doi.org/10.1016/j.jacc.2014.07.640.](https://doi.org/10.1016/j.jacc.2014.07.640) |
| 459 | Brown, 2014 | Excluded: wrong population | [“Sunday, 31 August 2014.” European Heart Journal 35, no. suppl 1 (September 2, 2014): 173–512. https://doi.org/10.1093/eurheartj/ehu323.](https://doi.org/10.1093/eurheartj/ehu323) |
| 460 | Rodriguez, 2014 | Excluded: wrong publication type | [“Sunday, 31 August 2014.” European Heart Journal 35, no. suppl 1 (September 2, 2014): 173–512. https://doi.org/10.1093/eurheartj/ehu323.](https://doi.org/10.1093/eurheartj/ehu323) |
| 461 | Shin, 2014 | Excluded: wrong outcome | [“Tuesday, 2 September 2014.” European Heart Journal 35, no. suppl 1 (September 2, 2014): 851–1187. https://doi.org/10.1093/eurheartj/ehu325.](https://doi.org/10.1093/eurheartj/ehu325) |
| 462 | Shin, 2014 | Excluded: wrong outcome | [Shin, D I, M H Jung, I J Choi, J S Yu, S M Seo, P J Kim, K Y Chang, K B Seung, and Y Ahn. “P144Impact of an Occluded Culprit Artery on the Long-Term Prognosis of Patients with Non-ST-Elevation Myocardial Infarction. Could They Be True STEMI-Equivalents?” Cardiovascular Research 103, no. suppl 1 (July 15, 2014): S25.3-S25. https://doi.org/10.1093/cvr/cvu082.83.](https://doi.org/10.1093/cvr/cvu082.83) |
| 463 | Bagai, 2014 | Excluded: wrong study design | [Bagai, Akshay, George D. Dangas, Gregg W. Stone, and Christopher B. Granger. “Reperfusion Strategies in Acute Coronary Syndromes.” Circulation Research 114, no. 12 (June 6, 2014): 1918–28. https://doi.org/10.1161/CIRCRESAHA.114.302744.](https://doi.org/10.1161/CIRCRESAHA.114.302744) |
| 464 | Koh, 2014 | Excluded: irrelevant topic | Koh, T.H. et al. Very long-term safety and efficacy outcomes of all comers BEACON II registry: Five-year follow-up and final report of the study. EuroIntervention. 2014 |
| 465 | Shin, 2014 | Excluded: wrong outcome | [Shin, D I, M H Jung, I J Choi, J S Yu, S M Seo, P J Kim, K Y Chang, K B Seung, and Y Ahn. “P144Impact of an Occluded Culprit Artery on the Long-Term Prognosis of Patients with Non-ST-Elevation Myocardial Infarction. Could They Be True STEMI-Equivalents?” Cardiovascular Research 103, no. suppl 1 (July 15, 2014): S25.3-S25. https://doi.org/10.1093/cvr/cvu082.83.](https://doi.org/10.1093/cvr/cvu082.83) |
| 466 | Geraci, 2014 | Excluded: wrong drug | Geraci, S. et al. Single-centre experience with 170 bioresorbable scaffolds in daily clinical practice: 6-to 12-month follow-up. EuroIntervention. 2014 |
| 467 | Milasinovic, 2014 | Excluded: wrong outcome | Milasinovic, D. et al. Comparison of clinical outcomes in patients with single versus multivessel coronary disease treated with bioresorbable polymer des. EuroIntervention. 2014 |
| 468 | Polad, 2014 | Excluded: wrong population | Polad, J. Outcomes from the largest multicentre prospective registry of des with bioabsorbable polymer. EuroIntervention. 2014 |
| 469 | Milewski, 2014 | Excluded: wrong study design | [Milewski, Krzysztof, and Buszman Pawel. “TCTAP C-141 Hybrid Revascularization Procedure in Patient with Multivessel Coronary Disease Presenting with Unstable Angina.” Journal of the American College of Cardiology 63, no. 12 (April 2014): S155–56. https://doi.org/10.1016/j.jacc.2014.02.412.](https://doi.org/10.1016/j.jacc.2014.02.412) |
| 470 | Man, 2014 | Excluded: wrong outcome | [Man, Sumche, Chinar Rahmattulla, Arie C. Maan, Niek H.J.J. Van Der Putten, W. Arnold Dijk, Erik W. Van Zwet, Ernst E. Van Der Wall, Martin J. Schalij, Anton P. Gorgels, and Cees A. Swenne. “Acute Coronary Syndrome with a Totally Occluded Culprit Artery: Relation of the ST Injury Vector with ST-Elevation and Non-ST Elevation ECGs.” Journal of Electrocardiology 47, no. 2 (March 2014): 183–90. https://doi.org/10.1016/j.jelectrocard.2013.11.009.](https://doi.org/10.1016/j.jelectrocard.2013.11.009) |
| 471 | Yudi, 2014 | Excluded: wrong outcome | [Yudi, Matias, Nick Andrianopoulos, Melanie Freeman, Chin Hiew, Bryan Yan, David Tsang, Julian Yeoh, Sandeep Prabhu, Caitlin Cheshire, and Andrew Ajani. “PM197 Clinical Characteristics, Trends and Outcomes in Elderly Patients with Acute Coronary Syndromes Undergoing PCI.” Global Heart 9, no. 1 (March 2014): e103. https://doi.org/10.1016/j.gheart.2014.03.1583.](https://doi.org/10.1016/j.gheart.2014.03.1583) |
| 472 | May, 2014 | Excluded: irrelevant topic | [May, Austin N., Alastair Carlyle, Mark Pitney, Sze Yuan Ooi, and Nigel Jepson. “PT187 Bioresorbable Scaffolds in the Treatment of Chronic Total Occlusions (CTO).” Global Heart 9, no. 1 (March 2014): e204. https://doi.org/10.1016/j.gheart.2014.03.1957.](https://doi.org/10.1016/j.gheart.2014.03.1957) |
| 473 | Tung, 2014 | Excluded: wrong population | Tung, Ying-Chang, Ping-Gune Hsiao, Lung-An Hsu, Chi-Tai Kuo, and Chi-Jen Chang. “Comparison between Exclusive and Selective Drug-Eluting Stent Strategies in Treating Patients with Multivessel Coronary Artery Disease.” *Acta Cardiologica Sinica* 30, no. 3 (May 2014): 181–89. |
| 474 | Smits, 2013 | Excluded: wrong population | [Smits, Pieter C., Kaiyum Sheikjoesoef, Kees-Jan Royaards, Georgios Vlachojannis, Elvin Kedhi, Jochem Wassing, Eugene P. McFadden, Carlos Van Mieghem, and Martin Van Der Ent. “TCT-13 Final Five Year Results From The All-Comer COMPARE Trial: A Prospective, Randomized Trial of Everolimus-Eluting vs. Paclitaxel-Eluting Stents.” Journal of the American College of Cardiology 62, no. 18 (October 2013): B5. https://doi.org/10.1016/j.jacc.2013.08.741.](https://doi.org/10.1016/j.jacc.2013.08.741) |
| 475 | Meliga, 2013 | Excluded: wrong population | [Meliga, Emanuele, Giacomo G. Boccuzzi, Maria Rosa Conte, Mauro De Benedictis, Andrea Gagnor, Azeem Latib, Primiano Lombardi, et al. “TCT-190 Biolimus Eluting StenT For de Novo coRonary Artery dIsease in patiENts with Diabetes mellituS: The BESTFRIENDS Multicentre Registry.” Journal of the American College of Cardiology 62, no. 18 (October 2013): B62. https://doi.org/10.1016/j.jacc.2013.08.924.](https://doi.org/10.1016/j.jacc.2013.08.924) |
| 476 | Zimarino, 2013 | Excluded: wrong study design | [Zimarino, Marco, Nick Curzen, Vincenzo Cicchitti, and Raffaele De Caterina. “The Adequacy of Myocardial Revascularization in Patients with Multivessel Coronary Artery Disease.” International Journal of Cardiology 168, no. 3 (October 2013): 1748–57. https://doi.org/10.1016/j.ijcard.2013.05.004.](https://doi.org/10.1016/j.ijcard.2013.05.004) |
| 477 | Ganyukov, 2013 | Excluded: wrong population | Ganyukov, V. et al. In-hospital outcomes of biventricular assist devices for high-risk percutaneous coronary intervention. J. Nucl. Cardiol. 2013 |
| 478 | Duarte, 2013 | Excluded: wrong study design | [“POSTER SESSION: CLINICAL.” European Journal of Heart Failure 12, no. S1 (May 2013). https://doi.org/10.1093/eurjhf/hst009.](https://doi.org/10.1093/eurjhf/hst009) |
| 479 | Sinkov, 2013 | Excluded: irrelevant topic | Sinkov, M. et al. In-hospital outcomes of biventricular assist devices for high-risk PCI. EuroIntervention. 2013 |
| 480 | Howard, 2013 | Excluded: wrong drug | [Howard, J P, D Jones, K S Rathod, D I Bromage, N Ding, S Gallagher, A K Jain, C J Knight, A Mathur, and A Wragg. “040 THE EFFECT OF GLYCOPROTEIN IIBIIIA INHIBITORS ON MORTALITY FOLLOWING PCI FOR NSTEMI/UA.” Heart 99, no. suppl 2 (May 2013): A29–30. https://doi.org/10.1136/heartjnl-2013-304019.40.](https://doi.org/10.1136/heartjnl-2013-304019.40) |
| 481 | Hwang, 2013 | Excluded: wrong drug | [Hwang, In Kyeom, Yun Kyung Kim, Seung-Woon Rha, Ji Eun Ra, Bong Soo Seo, Ji Kyoung Lee, Jin Oh Na, et al. “Impact of Insulin Resistance on 1-Year Clinical Outcomes in Non-Diabetic Patients Undergoing Percutaneous Coronary Intervention with Drug-Eluting Stents.” Journal of Cardiology 61, no. 2 (February 2013): 113–16. https://doi.org/10.1016/j.jjcc.2012.08.022.](https://doi.org/10.1016/j.jjcc.2012.08.022) |
| 482 | Hsieh, 2013 | Excluded: wrong population | [Hsieh, Victar, and Shamir R. Mehta. “How Should We Treat Multi-Vessel Disease in STEMI Patients?” Current Treatment Options in Cardiovascular Medicine 15, no. 1 (February 2013): 129–36. https://doi.org/10.1007/s11936-012-0213-6.](https://doi.org/10.1007/s11936-012-0213-6) |
| 483 | Schwietz, 2012 | Excluded: wrong outcome | [Schwietz, Thomas, Ioakim Spyridopoulos, Steven Pfeiffer, Rafael Laskowski, Sylvia Palm, Salvatore DE Rosa, Klotsche Jens, et al. “Risk Stratification Following Complex PCI: Clinical versus Anatomical Risk Stratification Including ‘Post PCI Residual SYNTAX-Score’ as Quantification of Incomplete Revascularization.” Journal of Interventional Cardiology 26, no. 1 (February 2013): 29–37. https://doi.org/10.1111/j.1540-8183.2013.12014.x.](https://doi.org/10.1111/j.1540-8183.2013.12014.x) |
| 484 | Kovacic, 2012 | Excluded: wrong outcome | [Kovacic, Jason C., Paul Lee, Rucha Karajgikar, Usman Baber, Birju Narechania, Javed Suleman, Pedro R. Moreno, Samin K. Sharma, and Annapoorna S. Kini. “Safety of Temporary and Permanent Suspension of Antiplatelet Therapy After Drug Eluting Stent Implantation in Contemporary ‘Real‐world’ Practice.” Journal of Interventional Cardiology 25, no. 5 (October 2012): 482–92. https://doi.org/10.1111/j.1540-8183.2012.00746.x.](https://doi.org/10.1111/j.1540-8183.2012.00746.x) |
| 485 | Cano, 2012 | Excluded: wrong outcome | [“ESICM 2012 MONDAY SESSIONS 15 October, 2012.” Intensive Care Medicine 38, no. S1 (October 2012): 1–327. https://doi.org/10.1007/s00134-012-2683-0.](https://doi.org/10.1007/s00134-012-2683-0) |
| 486 | Kaur, 2012 | Excluded: wrong drug | Kaur, S. et al. Anti-coagulant/anti-platelet mediated saphenous vein graft recanalization. J. Gen. Intern. Med. 2012 |
| 487 | De Bruyne, 2012 | Excluded: wrong study design | [De Bruyne, Bernard. “Multivessel Disease: From Reasonably Incomplete to Functionally Complete Revascularization.” Circulation 125, no. 21 (May 29, 2012): 2557–59. https://doi.org/10.1161/CIRCULATIONAHA.112.106872.](https://doi.org/10.1161/CIRCULATIONAHA.112.106872) |
| 488 | Santoso, 2012 | Excluded: wrong population | Santoso, T. et al. BEACON II-A prospective, multi-centre, observational, real-world registry to assess clinical outcomes of patients after treatment with the BioMatrixâ„¢ Stent. EuroIntervention. 2012 |
| 489 | Wessely, 2012 | Excluded: irrelevant topic | Wessely, R. et al. Safe and effective treatment of saphenous vein graft with a new generation DES: 2-year clinical outcomes. EuroIntervention. 2012 |
| 490 | Pesarini, 2012 | Excluded: wrong population | [Pesarini, Gabriele, Francesco Santini, Giulia Geremia, Carlo Zivelonghi, and Flavio Ribichini. “Risk Stratification in Acute Coronary Syndromes.” Journal of Cardiovascular Translational Research 5, no. 1 (February 2012): 1–10. https://doi.org/10.1007/s12265-011-9337-4.](https://doi.org/10.1007/s12265-011-9337-4) |
| 491 | Hawranek, 2011 | Excluded: wrong outcome | [“Monday, 29 August 2011.” European Heart Journal 32, no. Supplement 1 (August 2, 2011): 313–631. https://doi.org/10.1093/eurheartj/ehr323.](https://doi.org/10.1093/eurheartj/ehr323) |
| 492 | Bauer, 2011 | Excluded: wrong outcome | [“Wednesday, 31 August 2011.” European Heart Journal 32, no. Supplement 1 (August 2, 2011): 935–1118. https://doi.org/10.1093/eurheartj/ehr325.](https://doi.org/10.1093/eurheartj/ehr325) |
| 493 | Akkaya, 2011 | Excluded: irrelevant topic | [Akkaya, Emre. “The Impact of Chronic Kidney Disease on In-Hospital Clinical Outcomes in Patients Undergoing Primary Percutaneous Angioplasty for ST-Segment Elevation Myocardial Infarction.” Turk Kardiyoloji Dernegi Arsivi-Archives of the Turkish Society of Cardiology 39, no. 4 (June 1, 2011): 276–82. https://doi.org/10.5543/tkda.2011.01467.](https://doi.org/10.5543/tkda.2011.01467) |
| 494 | Jeger, 2011 | Excluded: wrong population | Jeger, R. V., and M. E. Pfisterer. “Primary PCI in STEMI--Dilemmas and Controversies: Multivessel Disease in STEMI Patients. Complete versus Culprit Vessel Revascularization in Acute ST--Elevation Myocardial Infarction.” *Minerva Cardioangiologica* 59, no. 3 (June 2011): 225–33. |
| 495 | Wessely, 2011 | Excluded: irrelevant topic | R. Wessely. Two-year clinical outcomes after treatment of saphenous vein graft with a new generation des. EuroIntervention. 2011 |
| 496 | Aranzulia, 2011 | Excluded: wrong study design | T.C. Aranzulla. Everolimus-eluting stent implantation for Unprotected Left Main disease: A single-centre experience. EuroIntervention. 2011 |
| 497 | Tayursky, 2011 | Excluded: wrong publication type | [“Abstracts: Suppl. 1 to Vol. 12 (May 20, 2011).” Interactive CardioVascular and Thoracic Surgery 12, no. Supplement 1 (May 1, 2011): S1–177. https://doi.org/10.1510/icvts.2011.0000S1.](https://doi.org/10.1510/icvts.2011.0000S1) |
| 498 | Ganyukov, 2011 | Excluded: wrong population | [“Abstracts: Suppl. 1 to Vol. 12 (May 20, 2011).” Interactive CardioVascular and Thoracic Surgery 12, no. Supplement 1 (May 1, 2011): S1–177. https://doi.org/10.1510/icvts.2011.0000S1.](https://doi.org/10.1510/icvts.2011.0000S1) |
| 499 | Maeno, 2011 | Excluded: wrong outcome | [Maeno, Yoshio, Naoyuki Yokoyama, Yoshitaka Shiratori, Hirosada Yamamoto, Syuichi Ishikawa, Akiyoshi Miyazawa, Ken Kozuma, and Takaaki Isshiki. “IMPACT OF PRIMARY PERCUTANEOUS CORONARY INTERVENTION FOR VERY ELDERLY PATIENTS WITH NON-ST ELEVATED-ACUTE CORONARY SYNDROME.” Journal of the American College of Cardiology 57, no. 14 (April 2011): E1769. https://doi.org/10.1016/S0735-1097(11)61769-5.](https://doi.org/10.1016/S0735-1097(11)61769-5) |
| 500 | Caixeta, 2011 | Excluded: wrong population | [Caixeta, Adriano, Tullio Palmerini, Philippe Genereux, Ecaterina Cristea, Alexandra J. Lansky, Roxana Mehran, George Dangas, et al. “PROGNOSTIC UTILITY OF THE SYNTAX SCORE IN PATIENTS WITH SINGLE VS. MULTIVESSEL DISEASE UNDERGOING PERCUTANEOUS CORONARY INTERVENTION: INSIGHTS FROM THE ACUITY TRIAL.” Journal of the American College of Cardiology 57, no. 14 (April 2011): E1773. https://doi.org/10.1016/S0735-1097(11)61773-7.](https://doi.org/10.1016/S0735-1097(11)61773-7) |
| 501 | Li, 2011 | Excluded: wrong study design | [Li, Yan, Haichang Wang, Chengxiang Li, Bo Xu, Junbo Ge, Weiyi Fang, Weimin Wang, and Shubin Qiao. “AS-096 Persistent Efficacy and Safety of Firebird Sirolimus-Eluting Stent in Patients with Complex Coronary Lesions—FIREMAN Registry 30-Month Clinical Outcomes Updates.” The American Journal of Cardiology 107, no. 8 (April 2011): 21A. https://doi.org/10.1016/j.amjcard.2011.02.046.](https://doi.org/10.1016/j.amjcard.2011.02.046) |
| 502 | Rahman, 2011 | Excluded: wrong outcome | [Rahman, Afzalur, Moinuddin Ahman, Moshin Ahmed, Habib Chaudhury, Sam Husnayen, Golam Azam, Nazmul Islam, Sania Hoque, and Anwarul Hoque Chowdhury. “AS-097 Clinical Outcome of New Zotarolimus-Eluting Stent in Complex Coronary Diseases: Result from a CRIBS Trial Substudy.” The American Journal of Cardiology 107, no. 8 (April 2011): 71A. https://doi.org/10.1016/j.amjcard.2011.02.168.](https://doi.org/10.1016/j.amjcard.2011.02.168) |
| 503 | Li, 2011 | Excluded: wrong drug | [Li, Yan, Haichang Wang, Chengxiang Li, Bo Xu, Junbo Ge, Weiyi Fang, Weimin Wang, and Shubin Qiao. “AS-096 Persistent Efficacy and Safety of Firebird Sirolimus-Eluting Stent in Patients with Complex Coronary Lesions—FIREMAN Registry 30-Month Clinical Outcomes Updates.” The American Journal of Cardiology 107, no. 8 (April 2011): 21A. https://doi.org/10.1016/j.amjcard.2011.02.046.](https://doi.org/10.1016/j.amjcard.2011.02.046) |
| 504 | Chen, 2010 | Excluded: wrong outcome | [Chen, Qingwei, Yan Yang, Ying Liu, Dazhi Ke, Qing Wu, and Guiqiong Li. “Safety and Effectiveness of Percutaneous Coronary Intervention (PCI) in Elderly Patients. A 5-Year Consecutive Study of 201 Cases with PCI.” Archives of Gerontology and Geriatrics 51, no. 3 (November 2010): 312–16. https://doi.org/10.1016/j.archger.2010.01.007.](https://doi.org/10.1016/j.archger.2010.01.007) |
| 505 | Stella, 2010 | Excluded: wrong outcome | [Stella, P. R., G. Pavlakis, P. Agostoni, H. M. Nathoe, S. Hoseyni Guyomi, B. J. Hamer, T. X. Wildbergh, P. A. Doevendans, and E. Van Belle. “One-year clinical follow-up of a registry evaluating a percutaneous revascularisation strategy combining a pre-specified simple selection process with the use of a new thin-strut bare cobalt-chromium stent.” Netherlands Heart Journal 18, no. 10 (October 2010): 486–92. https://doi.org/10.1007/BF03091820.](https://doi.org/10.1007/BF03091820) |
| 506 | Margato, 2010 | Excluded: wrong drug | Carvalho, Henrique, Paulino Sousa, Renato Margato, Sofia Carvalho, Hélder Ribeiro, Catarina Ferreira, and J. Moreira. “Initial Experience with the Presillion Stent in an Unselected Population: Immediate and Six-Month Outcomes.” *Revista Portuguesa de Cardiologia : Orgão Oficial Da Sociedade Portuguesa de Cardiologia = Portuguese Journal of Cardiology : An Official Journal of the Portuguese Society of Cardiology* 29 (April 1, 2010): 559–69. |
| 507 | Tomassini, 2010 | Excluded: wrong outcome | Tomassini, F. et al. Characteristics and long-term outcomes of elderly patients (>75 years old) with acute myocardial infarction complicated by cardiogenic shock undergoing percutaneous coronary interventions: A single centre retrospective study. EuroIntervention. 2010 |
| 508 | Gyongyosi, 2010 | Excluded: wrong outcome | [“Abstracts From the World Congress of Cardiology Scientific Sessions.” Circulation 122, no. 2 (July 13, 2010). https://doi.org/10.1161/CIRCULATIONAHA.110.192773.](https://doi.org/10.1161/CIRCULATIONAHA.110.192773) |
| 509 | Yan, 2010 | Excluded: wrong drug | [“Abstracts From the World Congress of Cardiology Scientific Sessions.” Circulation 122, no. 2 (July 13, 2010). https://doi.org/10.1161/CIRCULATIONAHA.110.192773.](https://doi.org/10.1161/CIRCULATIONAHA.110.192773) |
| 510 | Bangalore, 2010 | Excluded: wrong publication type | [Bangalore, Sripal, and David P. Faxon. “Coronary Intervention in Patients With Acute Coronary Syndrome: Does Every Culprit Lesion Require Revascularization?” Current Cardiology Reports 12, no. 4 (July 2010): 330–37. https://doi.org/10.1007/s11886-010-0115-8.](https://doi.org/10.1007/s11886-010-0115-8) |
| 511 | Varani, 2010 | Excluded: wrong drug | [Varani, Elisabetta, Paolo Guastaroba, Gian Di Tanna, Francesco Saia, Marco Balducelli, Gianluca Campo, Luigi Vignali, et al. “Long-Term Clinical Outcomes and Cost-Effectiveness Analysis in Multivessel Percutaneous Coronary Interventions: Comparison of Drug-Eluting Stents, Bare-Metal Stents and a Mixed Approach in Patients at High and Low Risk of Repeat Revascularisation.” EuroIntervention 5, no. 8 (April 2010): 953–61. https://doi.org/10.4244/EIJV5I8A160.](https://doi.org/10.4244/EIJV5I8A160) |
| 512 | Rha, 2010 | Excluded: wrong population | [Rha, Seung-Woon, Lin Wang, Ji Young Park, Kanhaiya L. Poddar, Sureshkumar Ramasamy, Byoung Geol Choi, Ji Bak Kim, et al. “AS-90: The Influence of Previous Myocardial Infarction on 12-Month Clinical Outcomes in Patients with Recurrent ST-Segment Elevation Acute Myocardial Infarction Undergoing Primary Percutaneous Coronary Intervention.” The American Journal of Cardiology 105, no. 9 (April 2010): 38A. https://doi.org/10.1016/j.amjcard.2010.01.128.](https://doi.org/10.1016/j.amjcard.2010.01.128) |
| 513 | Rahman, 2010 | Excluded: irrelevant topic | [Rahman, Afzalur, Moenuddin Ahmed, Moshin Ahmed, Sadat Chaudhury, Mohammad Husnayen, Golam Azam, and Nazmul Islam. “AS-192: Clinical Outcomes of the New Zotarolimus-Eluting Stent in Different Clinical Subjects.” The American Journal of Cardiology 105, no. 9 (April 2010): 79A-80A. https://doi.org/10.1016/j.amjcard.2010.01.239.](https://doi.org/10.1016/j.amjcard.2010.01.239) |
| 514 | Lopes, 2009 | Excluded: wrong population | [“Wednesday, 2 September 2009.” European Heart Journal 30, no. Supplement 1 (September 2, 2009): 845–1038. https://doi.org/10.1093/eurheartj/ehp416.](https://doi.org/10.1093/eurheartj/ehp416) |
| 515 | Rubartelli, 2009 | Excluded: wrong outcome | [“Wednesday, 2 September 2009.” European Heart Journal 30, no. Supplement 1 (September 2, 2009): 845–1038. https://doi.org/10.1093/eurheartj/ehp416.](https://doi.org/10.1093/eurheartj/ehp416) |
| 516 | Baget, 2009 | Excluded: wrong outcome | [“The Society for Cardiovascular Angiography and Interventions’ 32nd Annual Scientific Sessions.” Catheterization and Cardiovascular Interventions 73, no. S1 (June 2009). https://doi.org/10.1002/ccd.22076.](https://doi.org/10.1002/ccd.22076) |
| 517 | Fernandez, 2009 | Excluded: wrong outcome | [Fernández, Andrés, Ricardo Restrepo, Pablo Villa, Julián Garcés, and Gustavo Montero. “Angioplastia con stent vs. cirugía de revascularización coronaria en enfermedad multivaso (ACIRE).” Rev. colomb. cardiol, 2009, 53–63. http://www.scc.org.co/REVISTASCC/v16/v16n2/body/v16n2a2.htm.](http://www.scc.org.co/REVISTASCC/v16/v16n2/body/v16n2a2.htm) |
| 518 | Quercia, 2009 | Excluded: wrong publication type | [Quercia, Joseph H., Bryan G. Kane, and Kathleen E. Kane. “Simultaneous Very Late Angiographic Stent Thrombosis of 2 Drug-Eluting Stents: A Case Report.” The American Journal of Emergency Medicine 27, no. 1 (January 2009): 131.e5-131.e8. https://doi.org/10.1016/j.ajem.2008.04.027.](https://doi.org/10.1016/j.ajem.2008.04.027) |
| 519 | Hong, 2009 | Excluded: wrong drug | [Hong, Young Joon, Myung Ho Jeong, Youngkeun Ahn, Hae Chang Jeong, Shung Chull Chae, Seung Ho Hur, Taek Jong Hong, et al. “AS-65: Effects of Statins on 1-Year Cardiac Mortality after Drug-Eluting Stent Implantation in Diabetic Acute Myocardial Infarction Patients.” American Journal of Cardiology 103, no. 9 (April 22, 2009): 31B-32B. https://doi.org/10.1016/j.amjcard.2009.01.113.](https://doi.org/10.1016/j.amjcard.2009.01.113) |
| 520 | Shiba, 2009 | Excluded: wrong population | M. Shiba. Long-term results of percutaneous coronary intervention of chronic total occlusion in the optimal medical therapy era. Am. J. Cardiol.2009 |
| 521 | Shishehbor, 2008 | Excluded: wrong study design | [Shishehbor, Mehdi H., and Deepak L. Bhatt. “Treating Patients with Non-STEMI: Stent the Culprit Artery Only or Address All Lesions?” Current Treatment Options in Cardiovascular Medicine 10, no. 1 (February 2008): 93–97. https://doi.org/10.1007/s11936-008-0010-4.](https://doi.org/10.1007/s11936-008-0010-4) |
| 522 | Javaid, 2007 | Excluded: irrelevant topic | [Javaid, Aamir, Ashesh N. Buch, Daniel H. Steinberg, Tina Pinto Slottow, Probal Roy, Augusto D. Pichard, Lowell F. Satler, et al. “Does Creatine kinase‐MB (CK‐MB) Isoenzyme Elevation Following Percutaneous Coronary Intervention with Drug‐eluting Stents Impact Late Clinical Outcome?” Catheterization and Cardiovascular Interventions 70, no. 6 (November 15, 2007): 826–31. https://doi.org/10.1002/ccd.21248.](https://doi.org/10.1002/ccd.21248) |
| 523 | Huang, 2007 | Excluded: wrong publication type | Huang, R.L. Multivessel or culprit-only stenting in patients with unstable angina or NSTEMI: Commentary. J. Clin. Outcomes Manage. 2007 |
| 524 | Ozcan, 2007 | Excluded: wrong outcome | [Ozcan, Turkay, V. Gokhan Cin, Mustafa Yurtdas, Burak Akcay, Sabri Seyis, Armagan Acele, Dilek Cicek, Ahmet Camsari, Necdet Akkus, and Oben Doven. “Angiographic and Clinical Outcome Following Sirolimus-Eluting Stent (Cypher) Implantation A Single Center Experience: A Single Center Experience.” International Heart Journal 48, no. 1 (2007): 11–23. https://doi.org/10.1536/ihj.48.11.](https://doi.org/10.1536/ihj.48.11) |
| 525 | Lemos, 2007 | Excluded: wrong outcome | [Lemos, Pedro A., Expedito E. Ribeiro, Marco A. Perin, Luiz J. Kajita, Marco A. De Magalhães, João L. A. A. Falcão, Antonio Esteves Filho, et al. “Angiographic Segment Size in Patients Referred for Coronary Intervention Is Influenced by Constitutional, Anatomical, and Clinical Features.” The International Journal of Cardiovascular Imaging 23, no. 1 (February 2007): 1–7. https://doi.org/10.1007/s10554-006-9119-2.](https://doi.org/10.1007/s10554-006-9119-2) |
| 526 | Verna, 2006 | Excluded: wrong drug | [Verna, Edoardo, Mariangela Lattanzio, Sergio Ghiringhelli, Stefano Provasoli, and Salvatore Ivan Caico. “Performing versus Deferring Coronary Angioplasty Based on Functional Evaluation of Vessel Stenosis by Pressure Measurements: A Clinical Outcome Study:” Journal of Cardiovascular Medicine 7, no. 3 (March 2006): 169–75. https://doi.org/10.2459/01.JCM.0000215270.24649.de.](https://doi.org/10.2459/01.JCM.0000215270.24649.de) |
| 527 | Her, 2006 | Excluded: wrong drug | [Her, Sung-Ho, Ki-Bae Seung, Hee-Jeong Yoon, Dong-Bin Kim, Dong-Il Shin, Jong-Min Lee, Pum-Joon Kim, et al. “Prospective, Randomized, Preliminary Clinical Trial with Low-Molecular-Weight Heparin or Unfractionated Heparin as Periprocedural Anticoagulant during Elective Percutaneous Coronary Intervention.” Korean Circulation Journal 36, no. 8 (2006): 573. https://doi.org/10.4070/kcj.2006.36.8.573.](https://doi.org/10.4070/kcj.2006.36.8.573) |
| 528 | Davidavicius, 2005 | Excluded: wrong outcome | [Davidavicius, Giedrius, Frank Van Praet, Samer Mansour, Filip Casselman, Jozef Bartunek, Ivan Degrieck, Francis Wellens, et al. “Hybrid Revascularization Strategy: A Pilot Study on the Association of Robotically Enhanced Minimally Invasive Direct Coronary Artery Bypass Surgery and Fractional Flow Reserve-Guided Percutaneous Coronary Intervention.” Circulation 112, no. 9_supplement (August 30, 2005). https://doi.org/10.1161/CIRCULATIONAHA.104.524264.](https://doi.org/10.1161/CIRCULATIONAHA.104.524264) |
| 529 | Silber, 2005 | Excluded: wrong study design | [Silber, Sigmund, Per Albertsson, Francisco F. Avilés, Paolo G. Camici, Antonio Colombo, Christian Hamm, Erik Jørgensen, et al. “Guidelines for Percutaneous Coronary Interventions.” European Heart Journal 26, no. 8 (April 1, 2005): 804–47. https://doi.org/10.1093/eurheartj/ehi138.](https://doi.org/10.1093/eurheartj/ehi138) |
| 530 | Chen, 2005 | Excluded: wrong population | [Chen, Lin Y., Ryan J. Lennon, J. Aaron Grantham, Peter B. Berger, Verghese Mathew, Mandeep Singh, David R. Holmes, and Charanjit S. Rihal. “In-Hospital and Long-Term Outcomes of Multivessel Percutaneous Coronary Revascularization after Acute Myocardial Infarction.” The American Journal of Cardiology 95, no. 3 (February 2005): 349–54. https://doi.org/10.1016/j.amjcard.2004.09.032.](https://doi.org/10.1016/j.amjcard.2004.09.032) |
| 531 | Ochala, 2004 | Excluded: wrong outcome | Ochala, Andrzej, Grzegorz A. Smolka, Wojciech Wojakowski, Dariusz Dudek, Artur Dziewierz, Zbigniew Krolikowski, Zbigniew Gasior, and Michal Tendera. “The Function of the Left Ventricle after Complete Multivessel One-Stage Percutaneous Coronary Intervention in Patients with Acute Myocardial Infarction.” *The Journal of Invasive Cardiology* 16, no. 12 (December 2004): 699–702. |
| 532 | Mathew, 2004 | Excluded: wrong drug | [Mathew, Verghese, Bernard J. Gersh, Brent A. Williams, Warren K. Laskey, James T. Willerson, R. Thomas Tilbury, Barry R. Davis, and David R. Holmes. “Outcomes in Patients With Diabetes Mellitus Undergoing Percutaneous Coronary Intervention in the Current Era: A Report From the Prevention of REStenosis with Tranilast and Its Outcomes (PRESTO) Trial.” Circulation 109, no. 4 (February 3, 2004): 476–80. https://doi.org/10.1161/01.CIR.0000109693.64957.20.](https://doi.org/10.1161/01.CIR.0000109693.64957.20) |
| 533 | SzyguÅ‚a-Jurkiewicz, 2004 | Excluded: foreign language | Szyguła-Jurkiewicz, Bozena, Krzysztof Wilczek, Bartosz Szafron, Roman Przybylski, Bartosz Chudzik, Marcin Osuch, Marian Zebala, and Lech Poloński. “[In hospital observation of patients with acute coronary syndrome without ST elevation and multivessels coronary artery disease treated with early invasive strategy. Comparison of results of percutaneous coronary intervention and coronary artery by-pass grafting].” *Polskie Archiwum Medycyny Wewnetrznej* 112, no. 2 (August 2004): 911–18. |
| 534 | Morrison, 2003 | Excluded: wrong publication type | Morrison, D. A., and J. Sacks. “Balancing Benefit against Risk in the Choice of Therapy for Coronary Artery Disease. Lesson from Prospective, Randomized, Clinical Trials of Percutaneous Coronary Intervention and Coronary Artery Bypass Graft Surgery.” *Minerva Cardioangiologica* 51, no. 5 (October 2003): 585–97. |
| 535 | Watanabe, 2003 | Excluded: wrong population | [Watanabe, G., H. Takemura, S. Tomita, T. Misaki, and K. Kotoh. “Multiple Minimally Invasive Direct CABG for the Complete Revascularization: The Figure L Approach.” The Thoracic and Cardiovascular Surgeon 51, no. 01 (February 14, 2003): 28–32. https://doi.org/10.1055/s-2003-37271.](https://doi.org/10.1055/s-2003-37271) |
| 536 | Airoldi, 2002 | Excluded: wrong outcome | Airoldi, Flavio, Carlo Di Mario, Patrizia Presbitero, Luigi Maiello, Addolorata Carcagnì, Alessandro Bortone, Alberto Cremonesi, et al. “Elective Stenting in Small Coronary Arteries: Results of the Italian Prospective Multicenter Registry MICROSCOPE.” *Italian Heart Journal: Official Journal of the Italian Federation of Cardiology* 3, no. 7 (July 2002): 406–11. |
| 537 | Vlassov, 2001 | Excluded: irrelevant topic | Vlassov, G. P., C. S. Deyneka, N. O. Travine, V. H. Timerbaev, and A. S. Ermolov. “Acute Myocardial Infarction: OPCAB Is an Alternative Approach for Treatment.” *The Heart Surgery Forum* 4, no. 2 (2001): 147–50; discussion 150-151. |
| 538 | Choussat, 2001 | Excluded: wrong outcome | [Choussat, Rémi, Catherine Klersy, Alexander J.R Black, Irene Bossi, Jean-Pierre Laurent, Christian Jordan, Giulio Guagliumi, Jean Fajadet, and Jean Marco. “Long-Term (≥8 Years) Outcome after Palmaz-Schatz Stent Implantation.” The American Journal of Cardiology 88, no. 1 (July 2001): 10–16. https://doi.org/10.1016/S0002-9149(01)01577-6.](https://doi.org/10.1016/S0002-9149(01)01577-6) |
| 539 | Chauhan, 2001 | Excluded: irrelevant topic | [Chauhan, Manish S, Richard E Kuntz, Kalon K.L Ho, David J Cohen, Jeffrey J Popma, Joseph P Carrozza, Donald S Baim, and Donald E Cutlip. “Coronary Artery Stenting in the Aged.” Journal of the American College of Cardiology 37, no. 3 (March 2001): 856–62. https://doi.org/10.1016/S0735-1097(00)01170-0.](https://doi.org/10.1016/S0735-1097(00)01170-0) |
| 540 | Mathew, 2000 | Excluded: wrong population | [Mathew, Verghese, Peter B Berger, Ryan J Lennon, Bernard J Gersh, and David R Holmes. “Comparison of Percutaneous Interventions for Unstable Angina Pectoris in Patients with and without Previous Coronary Artery Bypass Grafting.” The American Journal of Cardiology 86, no. 9 (November 2000): 931–37. https://doi.org/10.1016/S0002-9149(00)01125-5.](https://doi.org/10.1016/S0002-9149(00)01125-5) |
| 541 | Akinci, 1999 | Excluded: wrong outcome | [Wendler, Olaf, Benno Hennen, Stefanos Demertzis, Torsten Markwirth, Dietmar Tscholl, Henning Lausberg, Qi Huang, Lennard Friedrich Dübener, Frank Langer, and Hans-Joachim Schäfers. “Complete Arterial Revascularization in Multivessel Coronary Artery Disease With 2 Conduits (Skeletonized Grafts and T Grafts).” Circulation 102, no. suppl_3 (November 7, 2000): Iii–79. https://doi.org/10.1161/circ.102.suppl_3.III-79.](https://doi.org/10.1161/circ.102.suppl_3.III-79) |
| 542 | Bersin, 1999 | Excluded: irrelevant topic | [Bersin, Robert M., John C. Cedarholm, Glen J. Kowalchuk, and Peter J. Fitzgerald. “Long-Term Clinical Follow-up of Patients Treated with the Coronary Rotablator: A Single-Center Experience.” Catheterization and Cardiovascular Interventions 46, no. 4 (April 1999): 399–405. https://doi.org/10.1002/(SICI)1522-726X(199904)46:4<399::AID-CCD3>3.0.CO;2-N.](https://doi.org/10.1002/(SICI)1522-726X(199904)46:4%3c399::AID-CCD3%3e3.0.CO;2-N) |
| 543 | Piana, 1999 | Excluded: wrong outcome | [Piana, Robert N, Waqar H Ahmed, Bernard Chaitman, Peter Ganz, Scott Kinlay, John Strony, Burt Adelman, and John A Bittl. “Effect of Transient Abrupt Vessel Closure during Otherwise Successful Angioplasty for Unstable Angina on Clinical Outcome at Six Months.” Journal of the American College of Cardiology 33, no. 1 (January 1999): 73–78. https://doi.org/10.1016/S0735-1097(98)00526-9.](https://doi.org/10.1016/S0735-1097(98)00526-9) |
| 544 | Metzger, 1994 | Excluded: wrong outcome | [Metzger, J. P., X. Tabone, J. L. Georges, C. Gueniche, J. P. Detienne, C. Le Feuvre, and A. Vacheron. “Coronary Angioplasty in Patients 75 Years and Older; Comparison with Coronary Bypass Surgery.” European Heart Journal 15, no. 2 (February 1994): 213–17. https://doi.org/10.1093/oxfordjournals.eurheartj.a060478.](https://doi.org/10.1093/oxfordjournals.eurheartj.a060478) |
| 545 | Voudris, 1993 | Excluded: wrong outcome | V. Voudris et al. Coronary angioplasty in the elderly: Immediate and long-term results. ANGIOLOGY. 1993 |
| 546 | Specchia, 1993 | Excluded: wrong publication type | Specchia, G., S. De Servi, M. L. Laudisa, P. Valentini, and F. Marsico. “[Coronary angioplasty in unstable angina].” *Cardiologia (Rome, Italy)* 38, no. 12 Suppl 1 (December 1993): 113–17. |
| 547 | Vernon, 1993 | Excluded: irrelevant topic | [Vernon Anderson, H. “Restenosis after Coronary Angioplasty.” Disease-a-Month 39, no. 9 (September 1993): 617–70. https://doi.org/10.1016/0011-5029(93)90002-K.](https://doi.org/10.1016/0011-5029(93)90002-K) |
| 548 | Wasserman, 1992 | Excluded: wrong publication type | Wasserman, H.S. Multivessel coronary angioplasty. J. CARDIOVASC. TECHNOL. 1992 |
| 549 | Colle, 1990 | Excluded: wrong outcome | [Colle, J. P., N. Delarche, F. Chague, F. Casteight, A. Choussat, and P. Besse. “Clinical Characteristics Affecting Success or Failure of PTCA in Patients with Multiple Vessel Disease and Poor Candidates for Surgery.” Clinical Cardiology 13, no. 11 (November 1990): 773–80. https://doi.org/10.1002/clc.4960131105.](https://doi.org/10.1002/clc.4960131105) |
| 550 | Hugenholtz, 1986 | Excluded: wrong outcome | Hugenholtz, P., P. Serruys, K. Laird-Meeter, and P. Fioretti. “Rational Management Following Diagnosis of Myocardial Ischemia.” *The Canadian Journal of Cardiology* Suppl A (July 1986): 242A-247A. |
| 551 | Waters, 1986 | Excluded: wrong outcome | [Waters, David D., Ann Walling, Denis Roy, and Pierre Théroux. “Previous Coronary Artery Bypass Grafting as an Adverse Prognostic Factor in Unstable Angina Pectoris.” The American Journal of Cardiology 58, no. 6 (September 1986): 465–69. https://doi.org/10.1016/0002-9149(86)90016-0.](https://doi.org/10.1016/0002-9149(86)90016-0) |
| 552 | Cowley, 1985 | Excluded: wrong outcome | [Cowley, M J, S M Mullin, S F Kelsey, K M Kent, A R Gruentzig, K M Detre, and E R Passamani. “Sex Differences in Early and Long-Term Results of Coronary Angioplasty in the NHLBI PTCA Registry.” Circulation 71, no. 1 (January 1985): 90–97. https://doi.org/10.1161/01.CIR.71.1.90.](https://doi.org/10.1161/01.CIR.71.1.90) |
| 553 | Kitamura, 1983 | Excluded: wrong outcome | Kitamura, S., A. Yamazaki, H. Tanoi, S. Umeda, K. Kawano, and Y. Sezai. “[Survival and late results following surgical treatment of coronary artery disease].” *Nihon Geka Gakkai Zasshi* 84, no. 9 (September 1983): 829–32. |
| 554 | Frick, 1982 | Excluded: wrong outcome | [Frick, M. H., P-T. Harjola, M. Valle, A. Järvinen, and P. Hekali. “Twelve Years of Coronary Bypass Surgery in Helsinki.” Acta Medica Scandinavica 212, no. S668 (1982): 7–12. https://doi.org/10.1111/j.0954-6820.1982.tb08516.x.](https://doi.org/10.1111/j.0954-6820.1982.tb08516.x) |
| 555 | Brener, 2002 | Excluded: wrong population | [Brener, Sorin J, Sabina A Murphy, C. Michael Gibson, Peter M DiBattiste, Laura A Demopoulos, and Christopher P Cannon. “Efficacy and Safety of Multivessel Percutaneous Revascularization and Tirofiban Therapy in Patients with Acute Coronary Syndromes.” The American Journal of Cardiology 90, no. 6 (September 15, 2002): 631–33. https://doi.org/10.1016/S0002-9149(02)02569-9.](https://doi.org/10.1016/S0002-9149(02)02569-9) |
| 556 | Kim, 2011 | Excluded: wrong outcome | [Kim, Min Chul, Myung Ho Jeong, Youngkeun Ahn, Jong Hyun Kim, Shung Chull Chae, Young Jo Kim, Seung Ho Hur, et al. “What Is Optimal Revascularization Strategy in Patients with Multivessel Coronary Artery Disease in Non-ST-Elevation Myocardial Infarction? Multivessel or Culprit-Only Revascularization.” International Journal of Cardiology 153, no. 2 (December 1, 2011): 148–53. https://doi.org/10.1016/j.ijcard.2010.08.044.](https://doi.org/10.1016/j.ijcard.2010.08.044) |
| 557 | Trabattoni, 2019 | Excluded: wrong outcome | Trabattoni, Daniela and Fabbiocchi, Franco and Montorsi, Piero and Galli, Stefano and Ravagnani, Paolo and Calligaris, Giuseppe and Teruzzi, Giovanni and Grancini, Luca and Troiano, Sarah and Ferrari, Cristina and Bartorelli, Antonio L. 2019. “A Long-Term Single-Center Registry of 6893 Patients Undergoing Elective Percutaneous Coronary Intervention With the Xience Everolimus-Eluting Stent.” *The Journal of invasive cardiology*. 31 (5): 146-151 |
| 558 | Garot, 2023 | Excluded: wrong outcome | [Garot P, Brunel P, Dibie A, Morelle JF, Abdellaoui M, Levy R, et al. Comparison of outcomes in patients with or without ARC-HBR criteria undergoing PCI with polymer-free biolimus coated stents: The BioFreedom France study. Catheter Cardiovasc Interv. 2023 Jan;101(1):60–71.](http://paperpile.com/b/79k7lV/5EYh) |
| 559 | Serota, 1991 | Excluded: wrong outcome | Serota, H., Deligonul, U., Lee, W.-H., Aguirre, F., Kern, M. J., Taussig, S. A., & Vandormael, M. G. (1991). Predictors of cardiac survival after percutaneous transluminal coronary angioplasty in patients with severe left ventricular dysfunction. *The American Journal of Cardiology*, *67*(5), 367–372. https://doi.org/10.1016/0002-9149(91)90043-K |
| 560 | Gaffar, 2017 | Excluded: wrong study design | [Gaffar, Rouan, Bettina Habib, Kristian B. Filion, Pauline Reynier, and Mark J. Eisenberg. “Optimal Timing of Complete Revascularization in Acute Coronary Syndrome: A Systematic Review and Meta‐Analysis.” Journal of the American Heart Association 6, no. 4 (April 5, 2017): e005381. https://doi.org/10.1161/JAHA.116.005381.](https://doi.org/10.1161/JAHA.116.005381) |
| 561 | Morrison, 2003 | Excluded: wrong publication type | Morrison, D. A., and J. Sacks. “Balancing Benefit against Risk in the Choice of Therapy for Coronary Artery Disease. Lesson from Prospective, Randomized, Clinical Trials of Percutaneous Coronary Intervention and Coronary Artery Bypass Graft Surgery.” *Minerva Cardioangiologica* 51, no. 5 (October 2003): 585–97. |
| 562 | Vaitkus, 1995 | Excluded: wrong study design dan publication type | [Vaitkus, P. T. (1995). The continuing evolution of percutaneous transluminal coronary angioplasty in the treatment of coronary artery disease. Coronary Artery Disease, 6(5), 429–439. https://doi.org/10.1097/00019501-199505000-00011](https://doi.org/10.1097/00019501-199505000-00011) |
