# Supplementary material for: A riddle of culprit only vs multivessel or immediate vs staged revascularization in patients with non-ST elevation acute coronary syndrome: A meta-analysis
Source: PLoS One. 2025 Mar 18;20(3):e0310695. doi: 10.1371/journal.pone.0310695 (PMC11918328; doi:10.1371/journal.pone.0310695)
Supplement: S3 table — (DOCX) [file pone.0310695.s003.docx]

# S4. Risk of bias assessment for observational studies (Newcastle-Ottawa Scale)

| *Study* | Selection | | | | Comparability | Outcome | | | Total (8/8) | Conclusion |
| --- | --- | --- | --- | --- | --- | --- | --- | --- | --- | --- |
|  | A | B | C | D | E | F | G | H |  |  |
| *Cohort studies* |  | | | | | | | | |  |
| *Elkady et al., 2021* | ★ | ★ | ★ | о | ★ | ★ | ★ | ★ | 7 | Good |
| *Alici et al., 2021* | ★ | ★ | ★ | о | о | ★ | ★ | ★ | 6 | Fair |
| *Baumann et al., 2022* | ★ | ★ | ★ | о | о | ★ | ★ | ★ | 6 | Fair |
| *Zapata et al., 2009* | ★ | ★ | ★ | о | о | ★ | ★ | ★ | 6 | Fair |
| *Kim Yong Hoon et al, 2020* | ★ | ★ | ★ | ★ | ★ | ★ | ★ | ★ | 8 | Good |
| *Mariani et al, 2001* | ★ | ★ | ★ | о | о | ★ | ★ | ★ | 6 | Fair |
| *Onuma et al., 2013* | ★ | ★ | ★ | ★ | о | ★ | ★ | ★ | 7 | Fair |
| *Pandit et al., 2022* | ★ | ★ | ★ | о | о | ★ | ★ | ★ | 6 | Fair |
| *Rathod et al., 2018* | ★ | ★ | ★ | ★ | ★ | ★ | ★ | ★ | 8 | Good |
| *Sadaka et al., 2019* | ★ | ★ | ★ | ★ | ★ | ★ | ★ | ★ | 8 | Good |
| *Small, 1988* | ★ | ★ | ★ | ★ | ★ | ★ | ★ | ★ | 8 | Good |
| *Brener, 2008* | ★ | ★ | ★ | ★ | о | ★ | ★ | ★ | 7 | Fair |
| *Yu, 2016* | ★ | ★ | ★ | ★ | о | ★ | ★ | о | 6 | Fair |
| *Pustjens, 2022* | ★ | ★ | ★ | ★ | ★ | ★ | ★ | ★ | 8 | Good |
| *Shishehbor, 2007* | **★** | **★** | **★** | **★** | **★** | **★** | **★** | **★** | **8** | Good |
| *Palmer, 2004* | **★** | **★** | **★** | **★** | **★** | **★** | **★** | **★** | **8** | Good |
| *Bauer, 2013* | ★ | **★** | **★** | **★** | **★** | **★** | **★** | **★** | **8** | Good |
| *Quadri, 2017* | **★** | **★** | о | о | **★** | **★** | **★** | **★** | **6** | Fair |
| *Correia, 2018* | **★** | **★** | **★** | **★** | **★** | **★** | **★** | **★** | **8** | Good |
|  |  |  |  |  |  |  |  |  |  |  |
| *Cross-sectional studies* |  |  |  |  |  |  |  |  | **Total (7/7)** |  |
| *Jarakovic, 2023* | ★ | ★ | о | о | о | ★ | ★ |  | 4 | Fair |
| *Omer, 2021* | ★ | ★ | ★ | ★ | о | ★ | ★ |  | 6 | Fair |
| *Wang, 2016* | ★ | ★ | ★ | ★ | ★ | ★ | ★ |  | 7 | Good |
| *Lee, 2011* | ★ | ★ | ★ | ★ | ★ | ★ | ★ |  | 7 | Good |
