# Supplementary material for: A riddle of culprit only vs multivessel or immediate vs staged revascularization in patients with non-ST elevation acute coronary syndrome: A meta-analysis
Source: PLoS One. 2025 Mar 18;20(3):e0310695. doi: 10.1371/journal.pone.0310695 (PMC11918328; doi:10.1371/journal.pone.0310695)
Supplement: S4 table — (DOCX) [file pone.0310695.s004.docx]

# S4. Risk of bias assessment for trial study (Cochrane Risk of Bias Tools for Trial)

| **Study** | **Risk of bias arising from the randomization process** | **Risk of bias due to deviations from the intended interventions** | **Risk of bias due to missing outcome data** | **Risk of bias in measurement of the outcome** | **Risk of bias in selection of the reported result** | **Overall risk of bias** |
| --- | --- | --- | --- | --- | --- | --- |
| Sardella, 2016 | 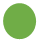 | 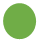 | 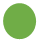 | 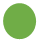 | 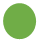 | Low |
| Hsieh, 2018 | 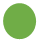 | 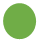 | 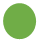 | 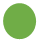 | 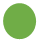 | Low |
| Hassanin, 2015 | 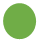 | 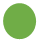 | 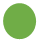 | 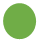 | 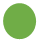 | Low |
