# Supplementary material for: A riddle of culprit only vs multivessel or immediate vs staged revascularization in patients with non-ST elevation acute coronary syndrome: A meta-analysis
Source: PLoS One. 2025 Mar 18;20(3):e0310695. doi: 10.1371/journal.pone.0310695 (PMC11918328; doi:10.1371/journal.pone.0310695)

# Funnel plot

1. CO vs MV all-cause mortality HR


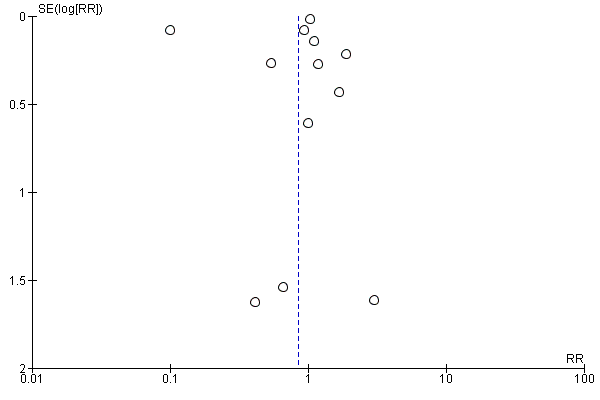


2. SS vs MS all-cause mortality


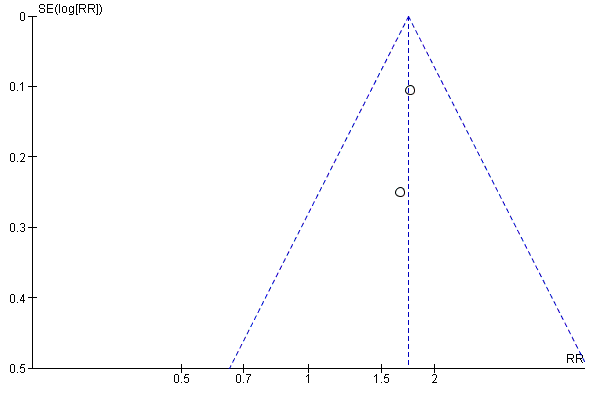


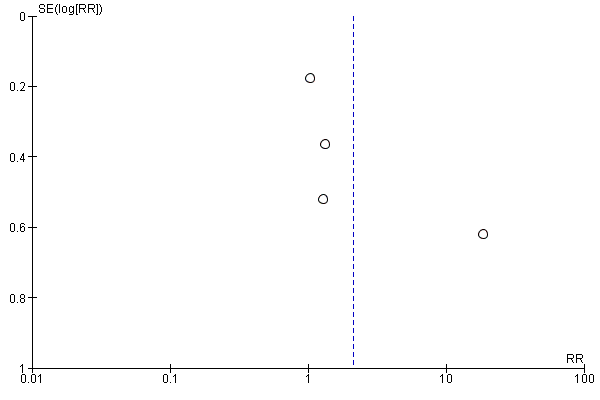
3. CO vs MV cardiac mortality

4. SS vs MS cardiac mortality


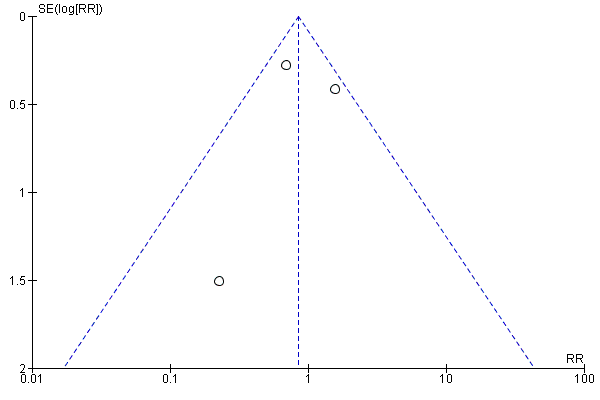


5. CO vs MV in-hospital mortality


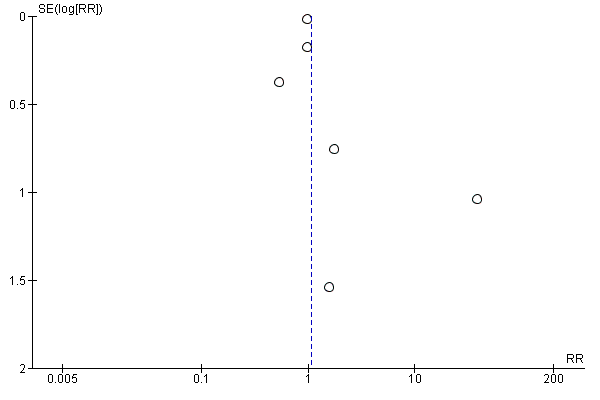


6. SS vs MS in-hospital mortality


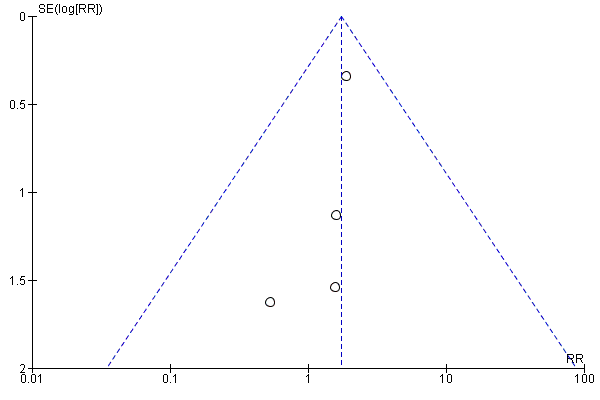


7. CO vs MV MACE


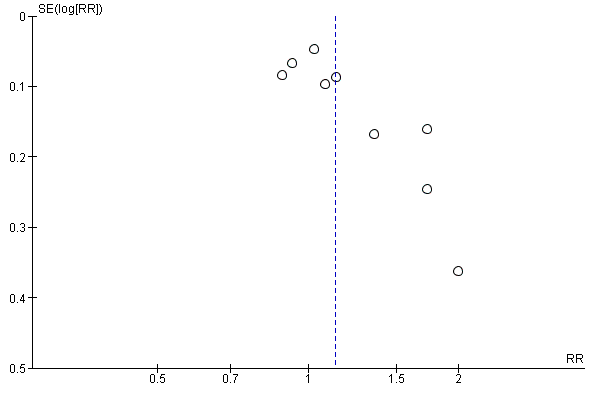


8. CO vs MV non-fatal MI


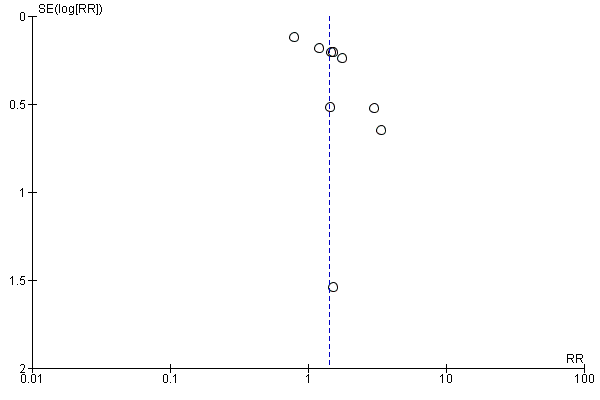


9. SS vs MS non-fatal MI


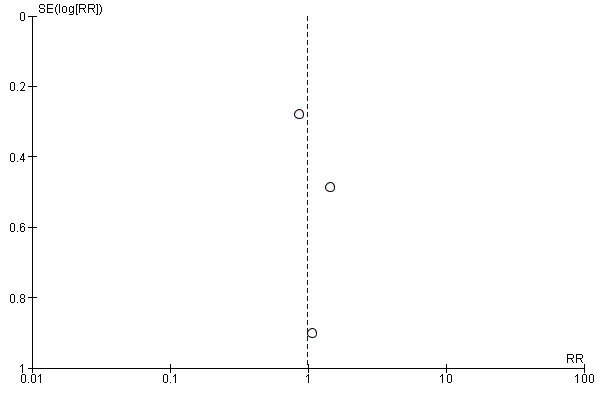


10. CO vs MV all repeat revascularization


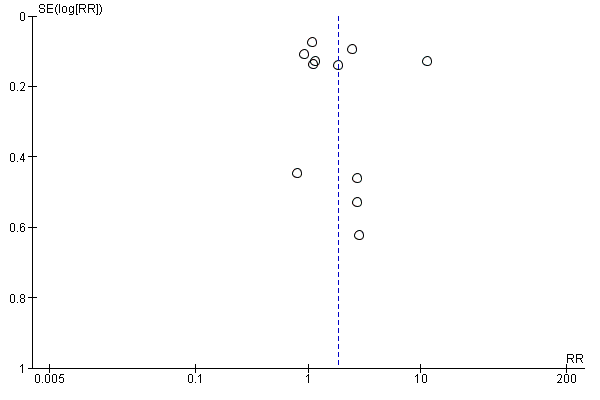


11. SV vs MV all repeat revascularization


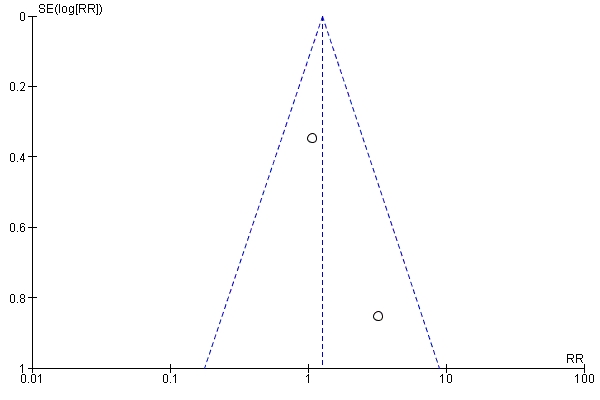


12. CO vs MV unplanned PCI


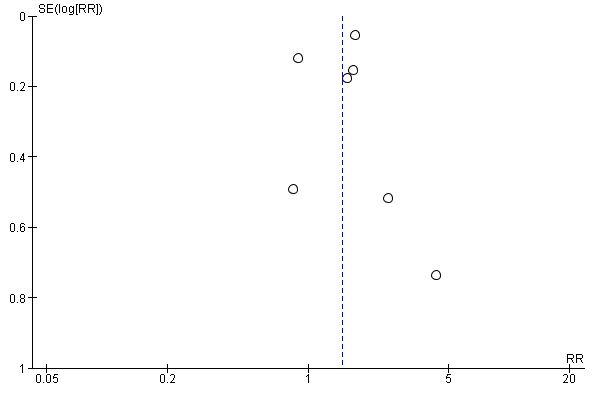


13. CO vs MV unplanned CABG


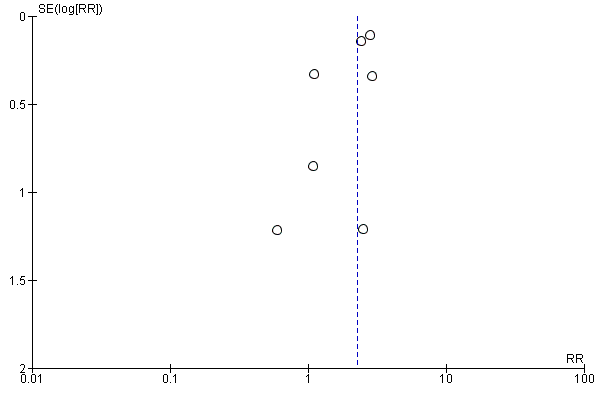


14. CO vs MV TLR repeat


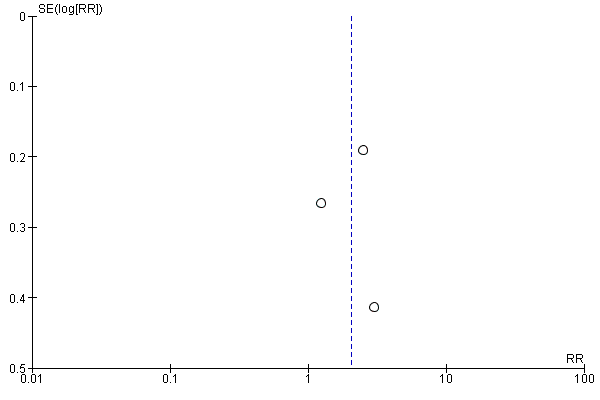


15. CO vs MV TVR repeat


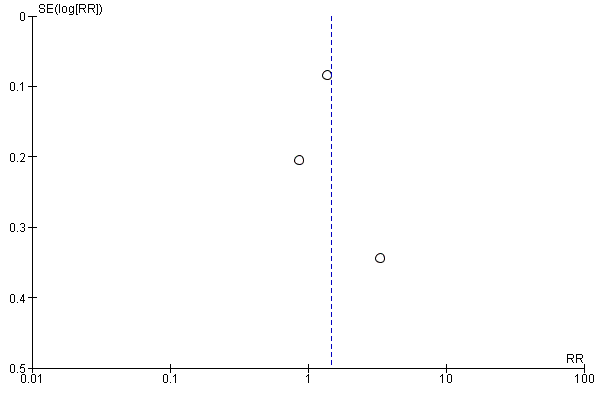


16. SV vs MV TVR repeat


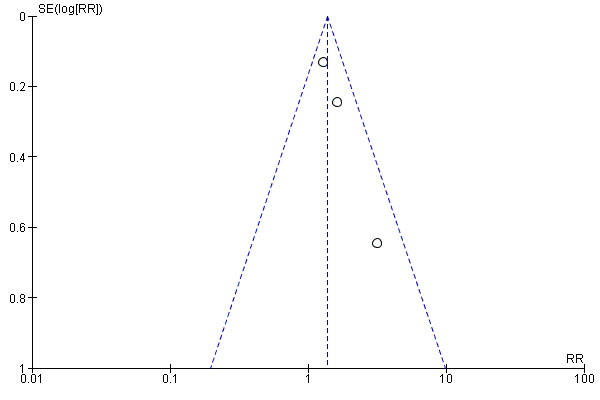


17. CO vs MV non TVR repeat


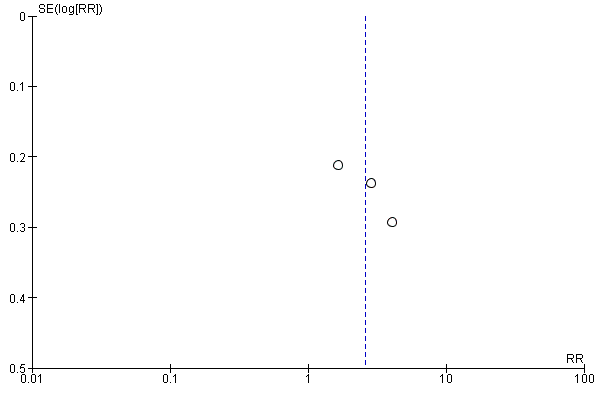

Supplement: S6 — (DOCX) [file pone.0310695.s006.docx]
